# Discovery of KB-0742, a Potent, Selective, Orally Bioavailable Small Molecule Inhibitor of CDK9 for MYC-Dependent Cancers

**DOI:** 10.1021/acs.jmedchem.3c01233

**Published:** 2023-11-15

**Authors:** David B. Freeman, Tamara D. Hopkins, Peter J. Mikochik, Joseph P. Vacca, Hua Gao, Adel Naylor-Olsen, Sonali Rudra, Huixu Li, Marius S. Pop, Rosa A. Villagomez, Christina Lee, Heng Li, Minyun Zhou, Douglas C. Saffran, Nathalie Rioux, Tressa R. Hood, Melinda A. L. Day, Michael R. McKeown, Charles Y. Lin, Norbert Bischofberger, B. Wesley Trotter

**Affiliations:** † Kronos Bio, Inc., 301 Binney Street, 2nd Floor East, Cambridge, Massachusetts 02142, United States; ‡ Certara Strategic Consulting, 100 Overlook Center, Suite 101, Princeton, New Jersey 08540, United States; § Naylor Olsen Consulting, LLC, 3369 Saddle Wood Court, Lansdale, Pennsylvania 19446, United States; ∥ TCG Lifesciences Private Limited, Block BN, Plot 7, Salt-lake Electronics Complex, Sector V, Kolkata 700091, West Bengal, India; ⊥ WuXi AppTec (Tianjin) Co., Ltd., 168 NanHai Road, 10th Avenue, TEDA, Tianjin 300457, P. R. China; △ Kronos Bio, Inc., 1300 So. El Camino Real Suite 400, San Mateo, California 94402, United States

## Abstract

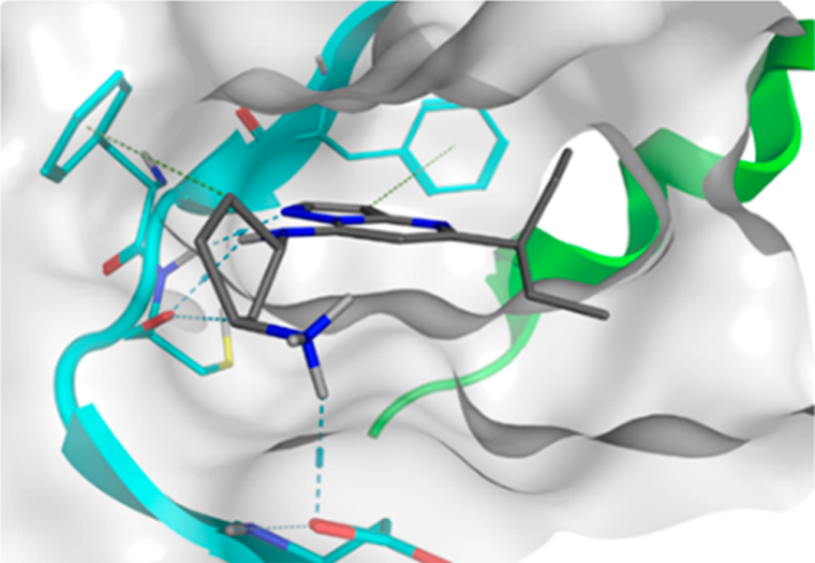

Transcriptional deregulation is a hallmark of many cancers and is exemplified by genomic amplifications of the MYC family of oncogenes, which occur in at least 20% of all solid tumors in adults. Targeting of transcriptional cofactors and the transcriptional cyclin-dependent kinase (CDK9) has emerged as a therapeutic strategy to interdict deregulated transcriptional activity including oncogenic MYC. Here, we report the structural optimization of a small molecule microarray hit, prioritizing maintenance of CDK9 selectivity while improving on-target potency and overall physicochemical and pharmacokinetic (PK) properties. This led to the discovery of the potent, selective, orally bioavailable CDK9 inhibitor **28** (**KB-0742**). Compound **28** exhibits *in vivo* antitumor activity in mouse xenograft models and a projected human PK profile anticipated to enable efficacious oral dosing. Notably, **28** is currently being investigated in a phase 1/2 dose escalation and expansion clinical trial in patients with relapsed or refractory solid tumors.

## Introduction

Cyclin-dependent kinases (CDKs) are a family of serine/threonine kinases responsible for cell cycle progression (CDK1, 2, 4, 6) and transcriptional regulation (CDK7, 8, 9, 12, 13).^1^ As both cell cycle control and transcription are recurrently altered in cancer, the essential regulatory roles of CDKs in these processes make them attractive targets for pharmacological intervention.^2,3^ Initial therapeutic strategies sought to target several CDKs with pan-CDK inhibitors such as alvocidib and seliciclib.^4^ These inhibitors exhibited mixed clinical success due to challenges associated with their polypharmacology, including toxicity related to cell cycle CDK inhibition. As an alternative approach, isoform-selective CDK inhibitors have potential to be safer and more effective, as evidenced by CDK4/6 inhibitors such as abemaciclib, palbociclib, and ribociclib in the treatment of estrogen receptor-positive/human epidermal growth factor receptor 2-negative (ER+/HER2−) metastatic breast cancer.^5,6^ Despite these successes with CDK inhibition as targeted cancer therapy, there remains significant unmet need in other tumor types with evident transcriptional deregulation, such as triple-negative breast cancer (TNBC), which is highly MYC dependent.^7,8^

Cyclin-dependent kinase 9 (CDK9) has emerged as an attractive CDK target owing to its role in potentiating oncogenic transcription programs.^9−13^ CDK9 is a transcription-regulating CDK that acts as a subunit of the positive transcription elongation factor b (P-TEFb).^14^ P-TEFb is recruited to the genome by transcription factors and other components of the transcription machinery, where it is sequestered in an inactive ribonucleotide protein complex (7SK snRNP).^15^ Its activation is facilitated through multiple molecular mechanisms, including recruitment of specific transcription factors such as MYC, transcription elongation-promoting complexes such as those including bromodomain-containing protein 4 (BRD4) and the super elongation complex (SEC), and by splicing factors and co-transcriptional RNA processing events.^16−19^ Activated CDK9 within the P-TEFb complex phosphorylates multiple transcription substrates—most notably the serine 2 (Ser2) residue of the carboxy-terminal domain (CTD) heptad repeats of RNA polymerase II (RNAP II). Phosphorylation of RNAP II Ser2 is an evolutionarily conserved and rate-limiting requirement for productive transcription elongation and mRNA processing.^20−22^ Although CDK9 is an essential gene and broadly required for global transcription, multiple studies have implicated highly transcribed, short half-life genes such as *MYC* and *MCL1* as being uniquely sensitive to CDK9 inhibition.^17,18,23^

The *MYC* family of protooncogenes (*MYC*, *MYCN*, and *MYCL*) includes the most commonly amplified genes in cancer and is associated with greater tumor aggressiveness across tumor types.^25,26^ These genes encode basic helix–loop–helix (bHLH) transcription factors involved in the regulation of cell growth, proliferation, and apoptosis. MYC forms a heterodimer with MAX, another bHLH transcription factor, and together they bind E-box consensus sequences (CACGTG) to effect target gene expression.^27^ Transcriptional initiation begins with MYC engaging E-box sites and recruiting the RNAP II complex, which is subsequently paused for further complex assembly.^18,28^ CDK9 releases this paused state by phosphorylating the RNAP II Ser2 and allowing transcriptional elongation to proceed, making CDK9 an attractive target to attenuate unchecked transcriptional addiction to deregulated MYC. There are several CDK9 inhibitors, each with varying CDK selectivity profiles, currently being evaluated in the clinic, including atuveciclib, VIP152, AZD4573, PRT2527, and GFH009, that aim to attenuate transcriptional deregulation.^29,30^ Interestingly, all of these compounds rely on intravenous (i.v.) administration.

Using our small molecule microarray (SMM) platform, we screened a library of small molecules for binding to transcriptional complexes in a cell lysate context. This led to identification of a CDK9 inhibitor hit molecule, **1** (KI-Arv-03), that exhibited excellent kinome and CDK isoform selectivity.^31^ Herein we report a medicinal chemistry effort to optimize this hit, prioritizing maintenance of CDK9 selectivity while improving the on-target potency and overall physicochemical and pharmacokinetic (PK) properties. This allowed us to advance the initial SMM hit to the potent, selective, orally bioavailable CDK9 inhibitor **28** (KB-0742). Compound **28** exhibits *in vivo* antitumor activity in multiple mouse xenograft models, including aggressive MYC-driven TNBC, a toxicology profile supporting clinical dosing, and projected human PK expected to enable efficacious oral dosing. Preclinical studies have advanced **28** to the clinic, where it is currently being investigated in a phase 1/2 dose escalation and cohort expansion study in participants with relapsed or refractory solid tumors or non-Hodgkin lymphoma (NCT04718675).

## Results and Discussion

**Figure 1:**
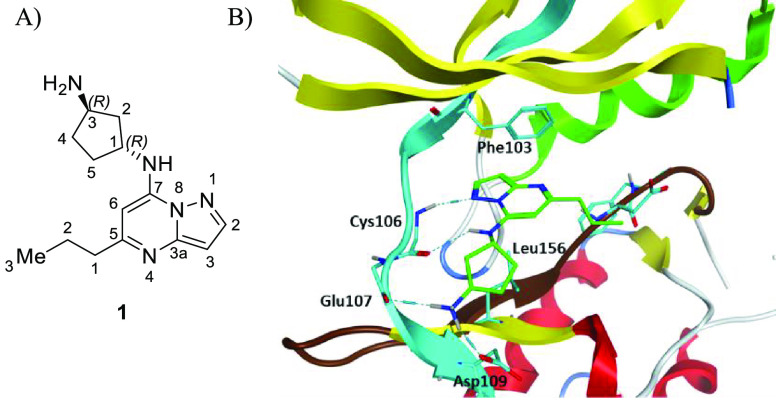
(A) Structure of **1** and (B) predicted docking in the CDK9/cyclinT1 ATP-competitive binding site from crystal structure PDB: 3MY1.

Previous work has described the identification of compound **1** via SMM screening of cell lysates and the discovery that this compound inhibited CDK9 with high isoform selectivity.^32^ Additional kinetic studies demonstrated ATP competition, suggesting that compound **1** binds in the catalytic cleft of CDK9. We therefore leveraged a structure-based drug design approach to further enhance the binding and physicochemical characteristics of the initial hit toward the ATP-competitive binding site. Docking of **1** into the ATP-binding site of CDK9/cyclin T1 from crystal structure PDB:3MY1 suggested binding in the catalytic cleft in a type I fashion (Figure 1).^33^

The compound is positioned with the amino-pyrazolopyrimidine core poised in a H-bond acceptor/donor orientation that interacts with hinge residue Cys106 (NH and CO). This fixed hinge-binding of the core structure then positioned the terminal primary amine in the proximity of solvent-exposed residues Glu107 and Asp109 for a H-bond donation or ionic interaction with the respective backbone carbonyl and Asp109 side chain. The 5-propyl moiety of the pyrazolopyrimidine points to a hydrophobic patch formed by the Leu156 side chain.

We sought to exploit this docking model to identify essential interactions that would impact potency and selectivity. Beginning with a minimal pharmacophore structure–activity relationship (SAR) campaign, we hypothesized that modification of three key regions of compound **1**, namely the C-3 (R_3_), C-5 (R_1_), and C-7 (R_2_) positions, would provide directional guidance for these early SAR efforts (Table 1 and Table 2). Using the hydrophobic isopropyl group as a standardized tool unit, we investigated the spatial requirements of the R_2_ group within the exit vector of the ATP-competitive binding site, specifically focusing on ligand interactions with Asp109 and Glu107 residues (Table 1). Shrinking the amino-cyclopentane ring of **1** to cyclobutane (compound **2**) resulted in a minimal decrease in potency against CDK9/cyclin T1. Amino-azetidine analogue **3** lost 5-fold potency. Removing an H-bond donor by methylating the azetidine (compound **4**) eliminated a Glu107 interaction and introduced hydrophobicity in the solvent-exposed exit vector, having a detrimental effect on potency. Oxetane moieties, as with **5**, exhibited potency similar to that of the azetidine (**3**) and further reinforced that the introduction of the methyl, rather than H-bond donor omission, drove the observed potency loss. Extension off the cyclobutane ring with a hydroxyl group as shown in **6** delivered an IC_50_ = 183 nM biochemical response, reinstating some lost potency as compared to that with cyclobutane only. Alkylation of the hydroxyl group had minimal effect, as illustrated by ethereal compound **7**.

**Table 1:**
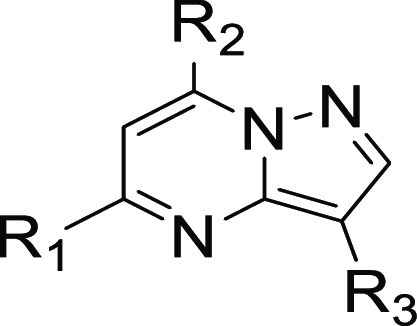

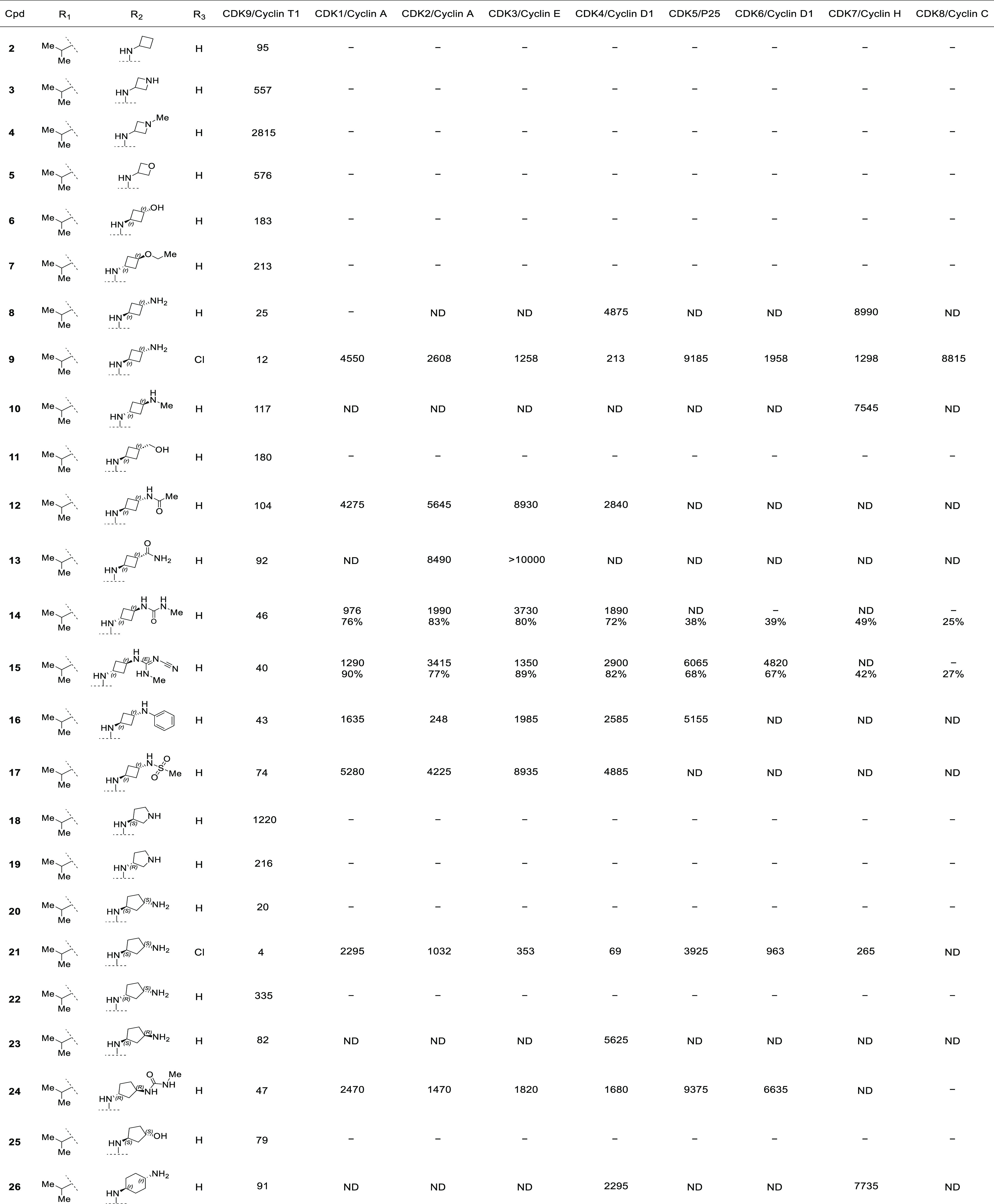
Structure and Activity^a^,^b^ against CDK Enzymes 1–9 of Compounds **2**–**26**

a Geometric means of two IC_50_ determinations per compound as shown in nM at 10 μM [ATP].

b Percent inhibition shown as geometric mean of two single-dose 10 μM compounds at CDK9/cyclin T1 *K*_m_ of 10 μM [ATP]. ND: IC_50_ not determined from collected data; − : no data collected.

**Table 2:**
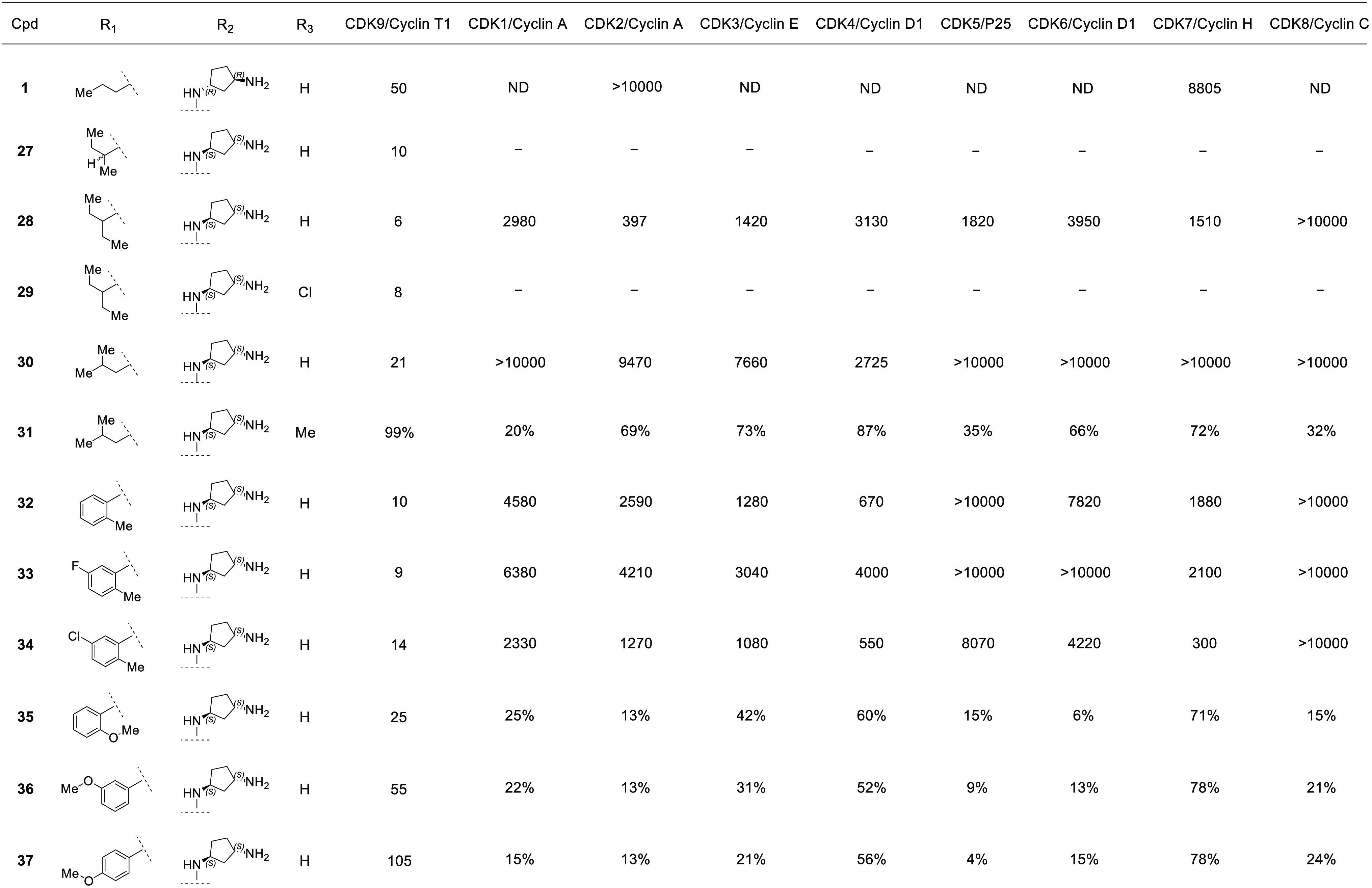
Structure and Activity^a^,^b^ against CDK Enzymes 1–9 of Compounds **1** and **27**–**37**

a Geometric means of two IC_50_ determinations per compound as shown in nM at 10 μM [ATP].

b Percent inhibition shown as geometric mean of two single-dose 10 μM compound at CDK9/cyclin T1 K_m_ of 10 μM [ATP]. ND: IC_50_ not determined from collected data. ‘-‘: no data collected.

Installation of a pendant amine, as in **8**, instead of a hydroxyl (**6**) enhanced potency by approximately 7-fold; we speculate that **8** allows for a more optimal interaction with Asp109 and Glu107 residues. Analogue **8** was then submitted to a CDK panel and displayed activity on CDK4 and CDK7, with 195-fold and 360-fold selectivity for CDK9, respectively. An analogue with a chlorine atom at the 3-position of the pyrazolopyrimidine (**9**) was more potent on CDK9 (12 nM) but lost selectivity, particularly on CDK4, where the fold selectivity dropped from 195-fold to 18-fold. Monomethylation analogue **10** lost potency as compared to **8**, demonstrating the need for two hydrogen atoms capable of H-bonding in the space between Asp109 and Glu107.

Changing the distance and electronic requirements of the ability to hydrogen bond with Asp109 and Glu107 residues within R_2_ had mixed effects. Extending the HBD/HBA group further toward the solvent with a hydroxy-methylene unit, as in **11**, approximated its non-methylene analogue **6**. The acetamide **12** and amide **13** saw little change relative to the monomethylated amine form **10**, suggesting that electronically manipulating the HBA ability on the nitrogen alone has little effect on potency. This idea was further enforced with methyl sulfonamide analogue **17**, which showed only a minor improvement of potency and a similar selectivity profile. However, the methyl urea **14** saw an increase in potency to 46 nM and a ≥21-fold selectivity toward the reference CDK panel. We suggest that both urea N–H groups can productively H-bond with Asp109. A similar observation can be seen with cyano-guanidine **15**, which improved the selectivity to ≥32-fold over other CDKs tested. Aniline **16** was equally potent to both cyano-guanidine and urea moieties but was less selective against CDK2.

We next investigated 5-membered rings, beginning with 3-aminopyrrolidines **18** and **19**. Interestingly, the (*R*)-enantiomer was approximately 6-fold more potent than the (*S*)-enantiomer. Switching back to the diaminocyclopentane series, as similarly seen in the initial SMM hit (**1**), we surprisingly found the (*S,S*)-configuration (**20**) to be exceptionally potent, rivaling that of diaminocyclobutane compound **8**. That same analogue with a chlorine atom attached to the 3-position of the pyrazolopyrimidine core (**21**) was even more potent at 4 nM against CDK9 but lost selectivity, particularly against CDKs 3, 4, and 7. Neither the (*R,S*)- nor (*S,R*)-diastereomers (**22** and **23**, respectively) were as potent as **20** against CDK9. The methylurea variation of the 5-membered-ring series (**24**) was similarly potent as the 4-membered-ring comparator (**14**), with a ≥31-fold selectivity toward the reference CDK panel. Hydroxylated aminocyclopentane **25** was more potent than its cyclobutane counterpart **6** but failed to show enhanced improvements over the diaminocyclobutane (**8**) or diaminocyclopentane (**20**) analogues. The diaminocyclohexane (**26**) demonstrated a decreased potency as compared to its cyclobutane (**8**) and cyclopentane (**20**) comparators, implying a ring size boundary for analogue binding. Based on our results, we believe that any R_2_ substitution that interacts with both Asp109 and Glu107 improves the hinge binding of the amino-pyrazolopyrimidine core and leads to increased biochemical potency. R_2_ substituents that bind only one residue (especially Asp109) tend to demonstrate decreased biochemical potency, presumably due to a weaker or more transient interaction of the amino-pyrazolopyrimidine core with hinge residue Cys106.

After assessing the tested R_2_ variations, we found the (*S,S*)-diaminocyclopentane series to be optimal. We next evaluated different R_1_ groups attached to the 5-position of the pyrazolopyrimidine core (Table 2). Based on our model, these variations would point to a hydrophobic patch within the CDK9/cyclin T1 formed by the Leu156 side chain. We embraced our minimal pharmacophore approach and incrementally enlarged the isopropyl group of **20**. A single methyl group extension to make the 2-butyl compound **27** as a mixture of diastereomers at the pseudo-benzylic carbon was tested and displayed enhanced potency,with a biochemical IC_50_ = 10 nM. An additional methyl to make symmetric 3-pentyl **28** delivered a 6 nM compound. A subsequent CDK selectivity panel demonstrated that this compound possessed a ≥66-fold selectivity toward CDK9 compared to others tested. The 3-chloro variant (**29**) did not show significantly improved activity against CDK9. Further alkyl variants, including 2-methylpropyl (compounds **30** and **31**), failed to show improvement beyond **28**.

**Figure 2:**
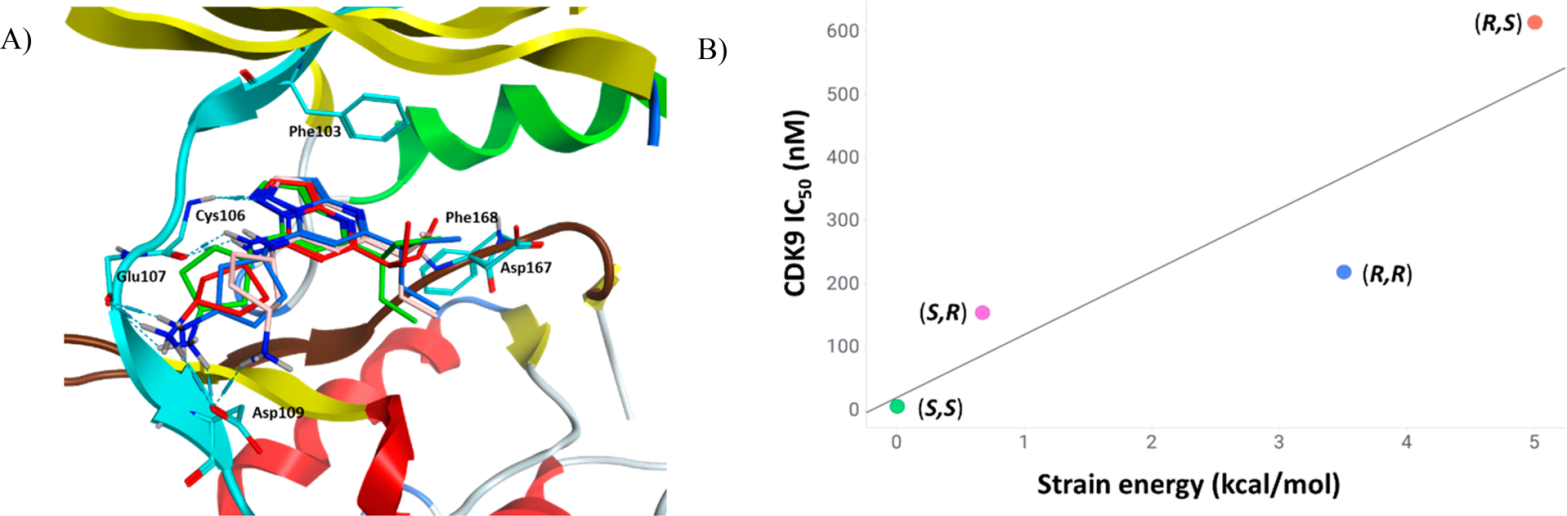
Computational model highlighting the associated strain energy of diamino-cyclopentane diastereomers within the ATP-competitive binding site. (a) Overlay of diamino-cyclopentane diastereomers **28**, **38**, **39**, and **40** within the ATP-competitive binding site of CDK9/cyclin T1. (b) Predicted strain energy (kcal/mol) plotted against the IC_50_ of each diastereomer.

We next evaluated aryl R_1_ groups and found *ortho-*substitution, especially with smaller groups such as methyl (**32**), to maintain potency and overall selectivity comparable to that of **28**. This substitution trend is further exemplified with *ortho-*, *meta-*, and *para-*anisole analogues **35**, **36**, and **37**, respectively. Interestingly, fluorinated tolyl compound **33** had a biochemical IC_50_ of 9 nM against CDK9 and an excellent selectivity profile, with ≥233-fold selectivity toward CDK9 over other tested CDKs. Switching to chlorine, as in compound **34**, maintained CDK9 potency compared to its fluorine counterpart **33** but significantly lost selectivity toward CDKs 4 and 7. Given its biochemical potency on CDK9 and selectivity within the tested CDK panel, **28** was chosen for further evaluation. The stereochemical requirement of the diamino-cyclopentane was assessed as illustrated in Table 3, showcasing the (*S,S*) configuration as the most potent configuration. Each isomer was then subjected to selectivity profiling. Table 4 illustrates the exquisite selectivity profile of compound **28** within the tested CDKs (selectivity profiling of compounds **38**, **39**, and **40** can be found on page S32 of the Supporting Information). We docked the four isomers into the ATP-competitive binding pocket of CDK9 and found the associated strain energy, respective to each isomer, to scale almost linearly according to potency, with the (*S,S*) configuration being the most energetically favored (Figure 2).

**Table 3:**
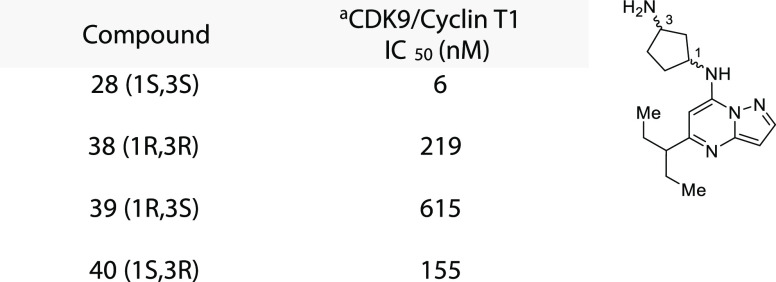
Diamino-cyclopentane Stereochemical Comparison against CDK9/Cyclin T1

a Enzymatic assay conducted at 10 μM ATP concentration (CDK9/cyclin T1 at *K*_m_).

**Table 4:** CDK Selectivity Profile of Compound **28**

**Enzyme**^a^	**[ATP] (μM)**	**IC**_**50**_**(nM)**
CDK9/Cyclin T1	10	6
CDK1/Cyclin A	10	2980
CDK2/Cyclin A	10	397
CDK3/Cyclin E	100	1420
CDK4/Cyclin D1	100	3130
CDK5/P25	10	1820
CDK6/Cyclin D1	100	3950
CDK7/Cyclin H	50	1510
CDK8/Cyclin C	10	>10000
CDK12/Cyclin K	30	589
CDK13/Cyclin K	5	372
CDK14 (PFTK1)/Cyclin Y	15	6250
CDK16/Cyclin Y	10	1580
CDK17/Cyclin Y (PCTK2)	20	2150
CDK18/Cyclin Y	20	1060
CDK19/Cyclin C	20	>10000

a Enzymatic assay conducted at *K*_m_ (μM) ATP concentration.

**Figure 3:**
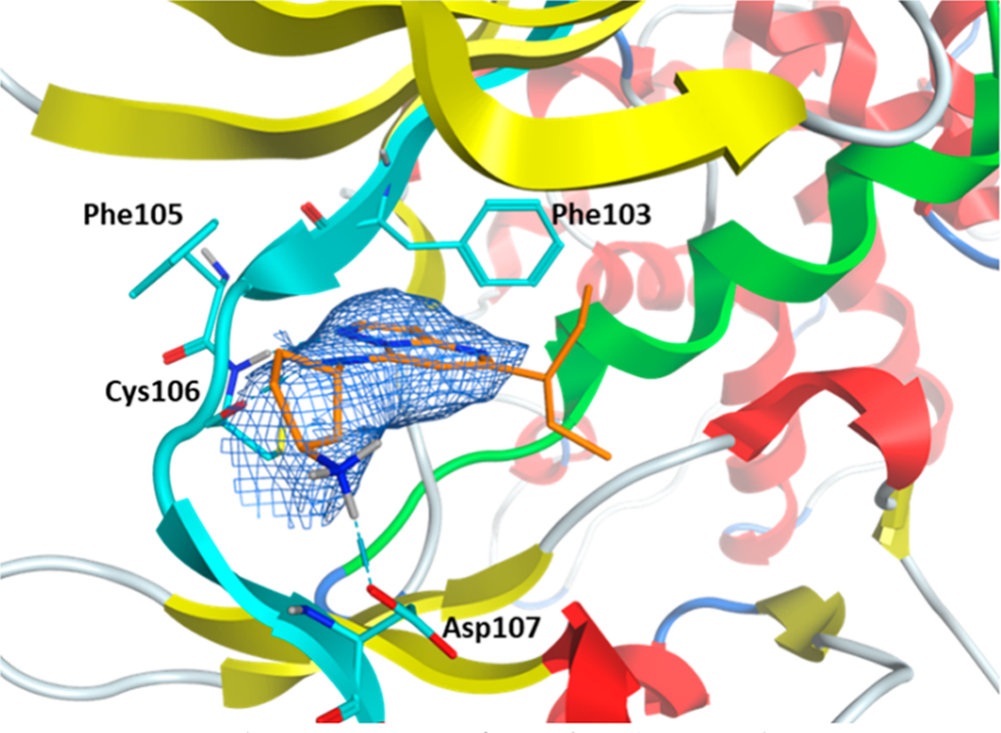
Crystal structure of **28** bound to the complex of CDK9 (cyan) and cyclin T1 (gray) (PDB: 8K5R). The ligand carbon atoms are shown in orange.

Co-crystallization of compound **28** with CDK9/cyclin T1 confirmed ligand binding as predicted in our model, albeit at 3.8 Å resolution (Figure 3). Regardless of the low resolution, the co-crystal structure provided a partial density to confirm our predicted binding mode of compound **28** (Figure 2). In brief, the amino-pyrazolopyrimidine core of **28** aligns to hinge Cys106 of the ATP-competitive binding pocket as postulated; the terminal amine of the diamino-cyclopentane moiety interacts with Asp109 in a HBD manner; and the 3-pentyl group points to a hydrophobic patch formed by the Leu156 side chain.

Compound **28** was further profiled in a kinase panel of 631 kinases (375 wild-type, 232 mutant, and 24 atypical kinases). It was found to retain selectivity as compared to SMM hit **1**, displaying enhanced potency toward CDK9/cyclin T1 with greater than 66-fold selectivity over all CDKs profiled and greater than 100-fold selectivity against cell-cycle CDKs 1, 3, 4, 5, and 6 (Supporting Information, pages S11–S32).^32^

The cellular activity of **28** was evaluated *in vitro* using TNBC cell culture models utilizing an assay that leverages fluorescently labeled antibodies and high-content fluorescence imaging to measure cell proliferation, apoptosis, and cell-cycle arrest in a multiplexed format. Compound **28** was found to inhibit cellular growth, displaying cytostatic (GI_50_ values ranging from 530 nM to 1 μM) and cytotoxic (IC_50_ values ranging from 600 nM to 1.2 μM) effects among the TNBC cell lines tested (Table 5). Notably, **28** potently induced apoptosis in 4 of 5 tested lines.

**Table 5:** Summary of Compound **28**’s Activity in TNBC Cell Lines^a^

	**Cell count**^b^		**Apoptosis**^c^		
**Cell line**	**GI**_**50**_**(μM)**	**IC**_**50**_**(μM)**	**5-Fold Induction (μM)**	**Emax**	d**G1/S cell cycle block (μM)**
BT-20	0.60	0.97	7.73	6.22	0.95
BT-549	0.80	0.94	0.74	7.99	1.42
MDA-MB-231	0.80	0.88	0.64	32.4	1.78
MT-3	0.53	0.60	0.67	15.1	1.16
Hs 578T	1.06	1.23	0.78	7.24	2.13

a BT-20, BT-549, MDA-MB-231, MT-3, and Hs 578T were treated with compound **28** in a serial dilution over 10 concentrations with a maximum of 0.1% DMSO over 72 h.

b Cell proliferation was measured by the fluorescence intensity of an incorporated nuclear dye.

c Apoptosis was measured by the fluorescence intensity of a fluorescently labeled antibody to activated caspase 3. The output is shown as a fold increase of apoptotic signal over vehicle background normalized to the relative cell count in each well. The concentration of test compound that caused a 5-fold induction in the caspase-3 signal is reported, indicating a significant apoptosis induction.

d Cell cycle arrest was measured by labeling of mitotic cells with a fluorescently labeled antibody to phosphorylated histone H3.

In addition, the physicochemical and ADME properties of **28** were evaluated (Table 6). Compound **28** possesses a molecular weight, logD, and solubility parameters within the acceptable ranges for drug-like oral compounds. The compound performed well in a Caco-2 assay at 2, 10, and 50 μM, demonstrating permeability with an efflux ratio ranging from 1.48 to 1.85. Inhibition assays against a panel of five cytochrome P450 enzymes (CYP3A4, CYP2D6, CYP2C9, CYP2C19, and CYP1A2) indicated no inhibition of CYP3A4, CYP2D6, CYP2C9, and CYP2C19 isoforms (IC_50_ > 30 μM) and weak activity against CYP1A2 (IC_50_ = 15.3 μM). There was no observed IC_50_ shift in time-dependent inhibition (TDI) of the six tested cytochrome P450 isoforms (CYP1A2, CYP2B6, CYP2C8, CYP2C9, CYP2C19, CYP2D6, and CYP3A4) in the presence or absence of NADPH at 0 or 30 min. Compound **28** demonstrated low intrinsic clearance (Cl_int_) in mouse, rat, dog, and human microsomes and low to moderate clearance in hepatocytes, resulting in *t*_1/2_ ≥ 2 h across these assays. Compound **28** exhibited a high free fraction in mouse and human plasma protein binding assays and was stable in plasma of all tested species beyond 360 min.

**Table 6:** Physical and *In Vitro* ADME Properties of Compound **28**

**Physical Properties**	
Molecular weight (g/mol)	287
Kinetic solubility, pH = 7.4 (μM)^a^	191
LogD, pH = 7.4^a^	0.9
TPSA (Å^2^)	68


**In Vitro ADME**^a^	
Caco-2 *P*_app_ A-B (10^–6^ cm/s)^b^	95.7–84.2
Caco-2 *P*_app_ B-A/A-B^b^	1.48–1.85
CYP3A4/2D6/2C9/2C19/1A2 IC_50_ (μM)	>30/>30/>30/>30/15.3
PPB m/r/d/h (% bound)	48/41/72/38
Plasma stability *t*_1/2_ m/r/d/h (min)	>360/2803/>360/>360
Microsome stability m/r/d/h (% remaining 180 min)	87.4/81.3/68.8/92.5
Microsome Cl_int_ m/r/d/h (mL/min/kg)^c^	7.0/5.5/5.5/0.93
Microsome stability *t*_1/2_ m/r/d/h (min)	775/510/283/1341
Hepatocyte Cl_int_ m/r/d/h (mL/min/kg)^d^	44/62/38/1.6
Hepatocyte stability *t*_1/2_ m/r/d/h (min)	374/118/195/2339

a Values are the geometric means of at least two replicates.

b Compounds were incubated at 2, 10, and 50 μM in cultured Caco-2 cells.

c Plasma protein binding were determined at 0.1, 1, and 10 μM. Data shown is the average across the tested concentrations.

d Liver microsome intrinsic clearance. Intrinsic clearance measured from fresh hepatocytes and cryopreserved human hepatocytes. PPB: plasma protein binding; Cl_int_: intrinsic clearance; m/r/d/h: mouse/rat/dog/human.

**Figure 4:**
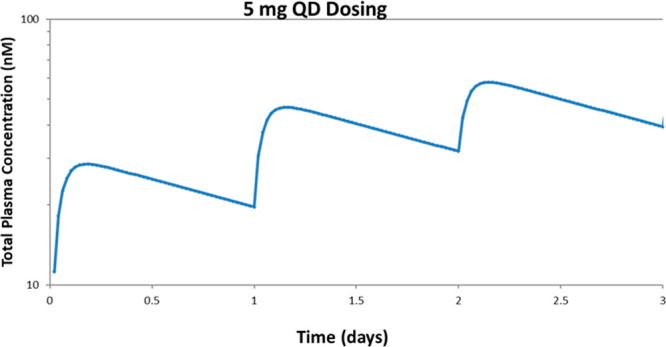
Simulated human pharmacokinetic (PK) profile for oral dosing of compound **28** at 5 mg daily with a clearance of 2 mL/min/kg and a volume distribution projection of 6 L/kg, giving a mean projected drug half-life of 35 h. Assumptions include PK linearity, one compartment, and 75% bioavailability.

Consistent with its favorable physicochemical and *in vitro* ADME properties, compound **28** exhibited good overall PK profiles in mouse, rat, and dog (Table 7). Plasma half-lives (*T*_1/2_) following intravenous dosing were 1.2 h in mouse, 2.4 h in rat, and 4.7 h in dog. The plasma clearance was low to moderate relative to hepatic blood flow in all preclinical species. Apparent steady state volumes of distribution (*V*_d,ss_) varied slightly across species, with values ranging from 3.9 L/kg in mouse to 7.3 L/kg in rat. The compound showed oral bioavailability following p.o. administration of a solution formulation in mouse (33%) and rat (84.5%), and high oral bioavailability in dog (>100%). A combination of *in vitro*–*in vivo* extrapolation and allometric methods was utilized for the projection of human PK parameters for **28**, with and without plasma protein binding corrections.^34−36^ Based on a 70 kg body weight, the mean predicted human plasma clearance (Cl) and *V*_d,ss_ are 2.0 mL/min/kg and 6.0 L/kg, respectively, together yielding an estimated terminal half-life of approximately 35 h, assuming one-compartment pharmacokinetics (half-life = [ln 2] × *V*_ss_/Cl). The predicted human oral bioavailability, derived from nonclinical species, is 75%. The projected long plasma half-life should enable **28** to achieve sustained exposures while avoiding high peak (*C*_max_) concentrations (Figure 4) as we explore dose escalation to a pharmacologically active dose in the clinic.

**Table 7:** Pharmacokinetic Parameters of Compound **28** in Mouse, Rat, and Dog

**Species**	***T***_**1/2**_**(i.v., h)**	**Cl**_**int**_**(i.v.,**mL/min/kg)	***V*_d,ss_(i.v.,**L/kg)	**C**_**max**_**(p.o.,** ng/mL)	**AUC (p.o.,** ng·h/mL)	***F*** **(%)**
Mouse^a^	1.2	23.4	3.9	501	1700	33
Rat^b^	2.4	51.7	7.3	411	3591	84.5
Dog^c^	4.7	13.4	4.5	494	4007	>100

a Vehicle used in intravenous and oral administration studies: EtOH, PEG400, HPβCD, 1/2/7 (v/v/v) at 1.0 mg/kg **28** for i.v. and 20 mg/kg **28** for p.o.

b Vehicle used in intravenous and oral administration studies: 2.0 mg/kg **28** at pH 3.0 in 50 mM citrate buffer for i.v. and 10 mg/kg **28** at pH 3.0 in 50 mM citrate buffer for p.o.

c Vehicle used in intravenous and oral administration studies: 0.50 mg/kg **28** in saline for i.v. and 2.5 mg/kg **28** in saline for p.o. *T*_1/2_: half-life; Cl_int_: intrinsic clearance; *V*_d,ss_: volume of distribution; *F*: bioavailability

**Figure 5:**
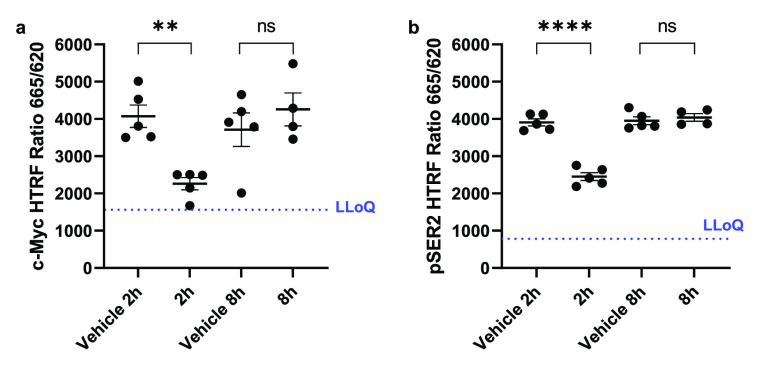
Compound **28** decreased the levels of MYC (a) and pSER2 (b) in TNBC PDX CTG-1017 subcutaneous tumors. Tumor-bearing Athymic Nude-Foxn1^nu^ mice were collected at 2 and 8 h post-terminal dose of either vehicle or 60 mg/kg **28** p.o. using an intermittent dosing schedule of 3-days on, 4-days off for up to 4 weekly cycles. Tumor lysates were prepared, and levels of MYC (a) and pSER2 (b) were determined using their respective HTRF assays. The plasma concentration of **28** was 846 ng/mL (2.9 μM) and 73 ng/mL (0.25 μM) at 2 and 8 h post-dose, respectively. Data are presented as mean ± SEM, *n* = 5. *P < 0.05; **P < 0.005; ***P < 0.0005; ****P < 0.0001. hr: hour; HTRF: homogeneous time-resolved fluorescence; LLoQ: lower limit of quantification; ns: not significant.

Next, we sought to assess the pharmacodynamic (PD) effects and efficacy of **28** on *in vivo* tumor growth in MYC-amplified TNBC patient-derived xenograft (PDX) models. To assess the on-target PD of **28**, we examined MYC levels and phosphorylation of RNAP II Ser 2 (pSER2) as downstream indicators of CDK9 activity in xenograft tumor samples. Both MYC and pSER2 were significantly reduced by 2 h post-terminal dose of **28** while plasma concentration of **28** was still high (Figure 5), consistent with CDK9 inhibition. By 8 h post-administration, both MYC and pSER2 levels were no longer significantly different from vehicle. This observation combined with the low plasma levels of compound **28** suggests target engagement with downstream effects on MYC and pSER2.

**Figure 6:**
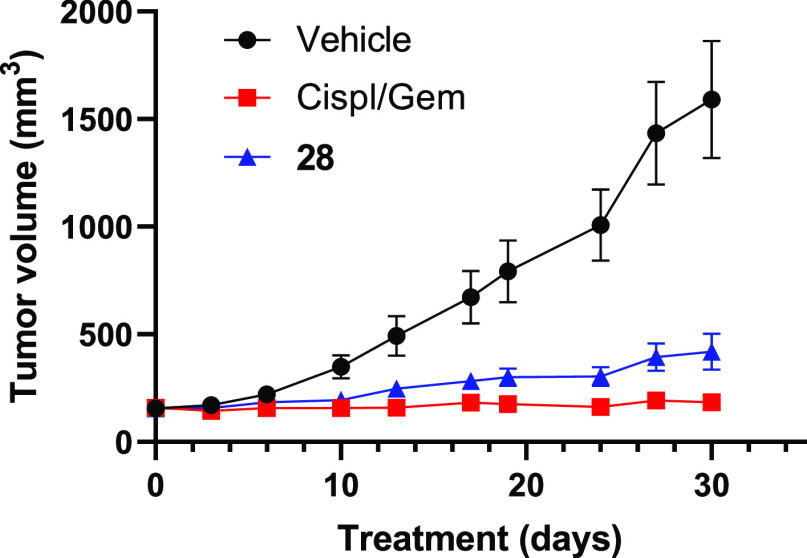
*In vivo* tumor growth inhibition for compound **28** in the TNBC model CTG-1017. Athymic Nude-Foxn1^nu^ mice bearing established subcutaneous PDX tumors (mean starting tumor volume of 157 mm^3^) were treated with vehicle (saline), **28**, or SOC chemotherapeutics (cisplatin and gemcitabine). Vehicle and **28** at 60 mg/kg were administered orally using an intermittent dosing schedule of 3-days on, 4-days off for up to 4 weekly cycles (day 30 TGI = 82%, *p* = 0.0001 vs vehicle). SOC were administered as follows: cisplatin 5 mg/kg i.p. Q7D×3 + gemcitabine 100 mg/kg i.p. Q7D×3. The corresponding mean body weight over time graph can be found in the Supporting Information.

We evaluated *in vivo* anti-tumor activity of **28** in human *MYC*-amplified PDX models, focusing on TNBC cell lines CTG-1017 (Figure 6), CTG-0869, and CTG-0437 (Supporting Information, page S34). Athymic Nude-Foxn1^nu^ mice bearing established subcutaneous PDX tumors were treated with vehicle, standard of care (SOC) chemotherapeutics, or **28**. Tumor growth inhibition by **28** was observed in all models, with tumor growth inhibition by **28** being comparable to SOC for both CTG-1017 and CTG-0437 models, highlighting that the observed on-target PD effects correspond with significant antitumor effects *in vivo*.

## Conclusion

From discovery hit **1**, we report the optimization of an SMM screening hit using a structure-based drug design approach that led to the discovery of clinical candidate **28** (**KB-0742**), a potent, selective, orally bioavailable CDK9 inhibitor. Compound **28** has pharmacological and physicochemical properties and exhibits *in vivo* antitumor activity in TNBC mouse xenograft models. *In vitro*, **28** reduced MYC and pSER2 in tumor lysates in a concentration-dependent manner and substantially inhibited *in vivo* tumor growth at the same dose that reduced levels of these PD markers. Further, **28** has a PK profile that projects sustained human exposure conducive to efficacious oral dosing in the clinic. These findings have advanced **28** to the clinic in an ongoing phase 1/2 dose escalation and expansion trial in patients with relapsed or refractory solid tumors or non-Hodgkin’s lymphoma (NCT04718675).

## Experimental Section

### Synthetic Chemistry

**Figure 7:**
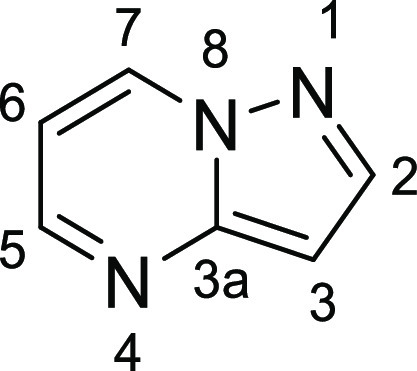
Numbering convention used for pyrazolo[1,5-*a*]pyrimidine core.

**Scheme 1:**
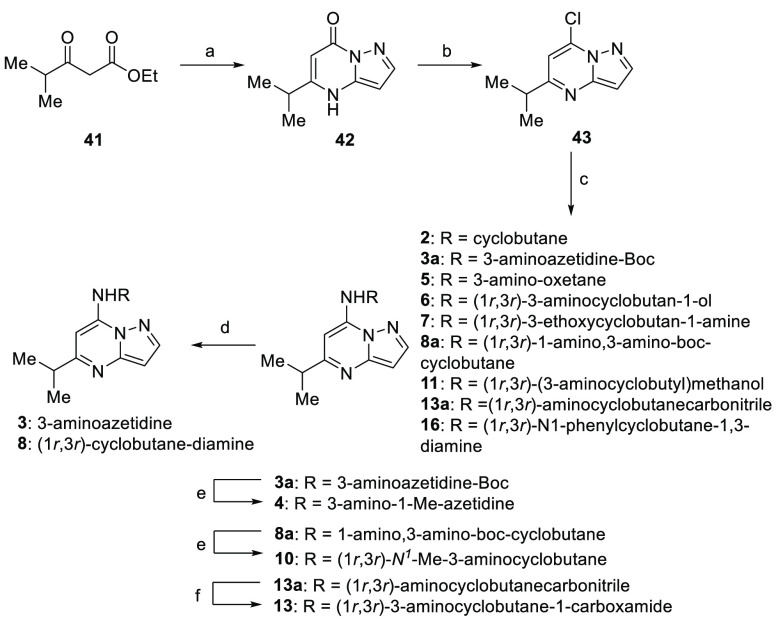
Synthesis of Four-Membered-Ring Minimal Pharmacophore Derivatives through Amination of Chloro-pyrazolo[1,5-*a*]pyrimidine **43** Reagents and conditions: (a) 1*H*-pyrazol-5-amine, AcOH, reflux; (b) POCl_3_, pyridine, DMAP, MeCN, reflux; (c) RNH_2_, K_2_CO_3_, MeCN, reflux; (d) HCl, 1,4-dioxane, 0 °C; (e) LiAlH_4_, THF, 0–80 °C; (f) Ghaffar–Parkins catalyst, H_2_O, THF, 70 °C.

**Scheme 2:**
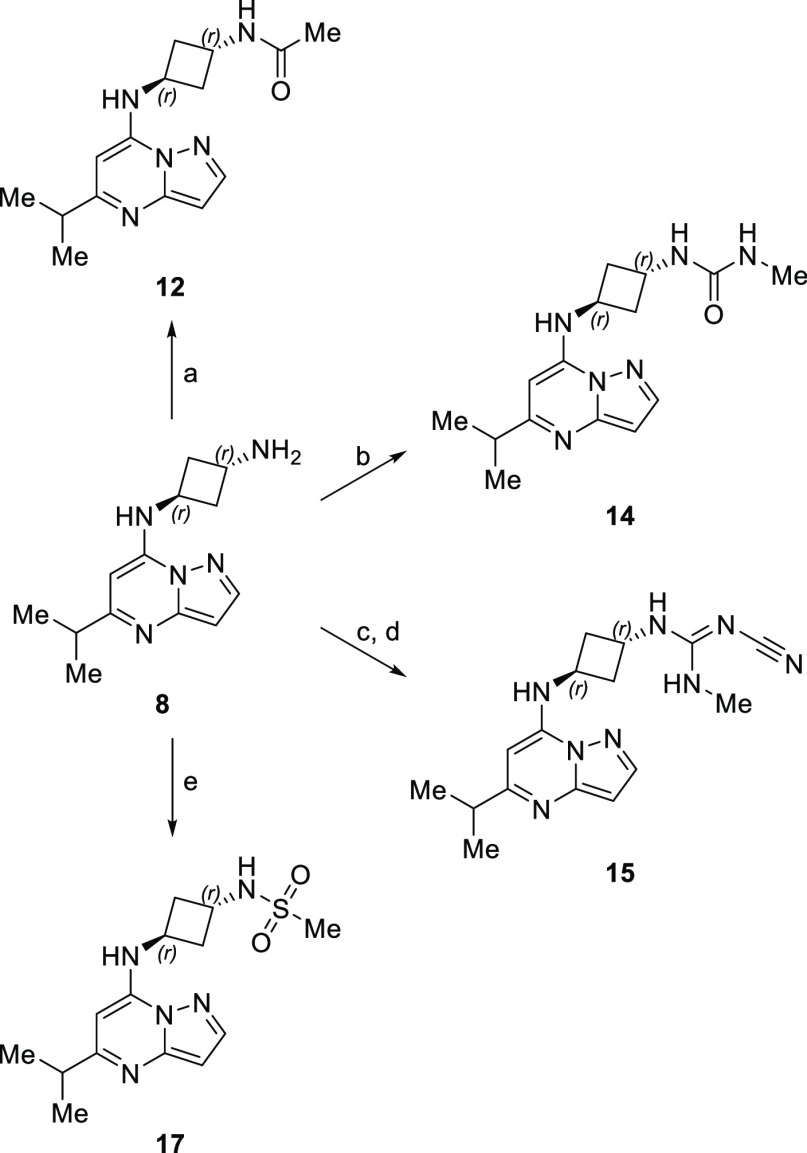
Modular Elaboration of Aminocyclobutane **8** Reagents and conditions: (a) AC_2_O, pyridine; (b) *S*-methyl methylaminomethanethioate, 1,4-dioxane, H_2_O, 65 °C; (c) diphenyl *N*-cyanocarbonimidate, *i*PrOH, 70 °C; (d) MeNH_2_, *i*PrOH, 70 °C; (e) MeSO_2_Cl, NaHCO_3_, DIPEA, CH_2_Cl_2_.

**Scheme 3:**
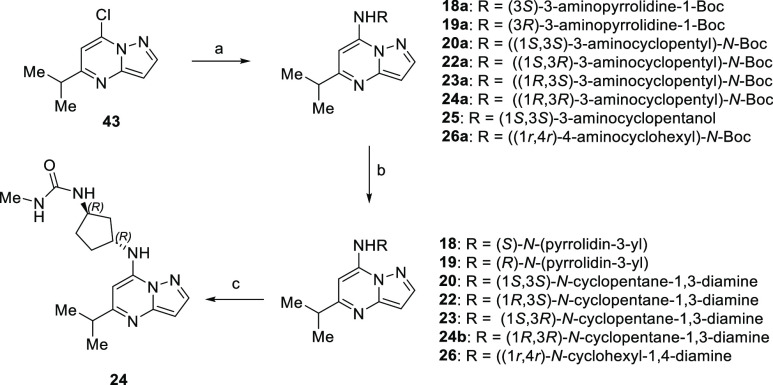
Synthesis of Five- and Six-Membered-Ring Minimal Pharmacophore Derivatives through Amination of Chloro-pyrazolo[1,5-*a*]pyrimidine **43** Reagents and conditions: (a) RNH_2_, K_2_CO_3_, MeCN, reflux; (b) HCl, 1,4-dioxane, 0 °C; (c) *S*-methyl *N*-methylcarbamothioate, 1,4-dioxane, H_2_O, 65 °C.

**Scheme 4:**
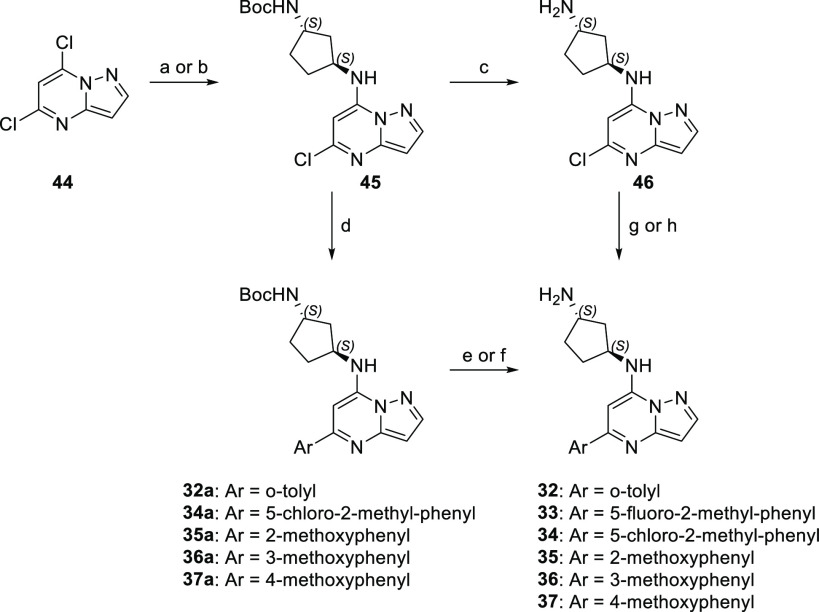
Suzuki–Miyaura Coupling Route to 5-Aryl-Substituted Pyrazolo[1,5-*a*]pyrimidine Analogues Reagents and conditions: (a) *N*^1^-Boc-((1*S*,3*S*)-3-aminocyclopentyl), TEA, MeCN, 50 °C; (b) *N*^1^-Boc-(1*S*,3*S*)-3-aminocyclopentyl), K_2_CO_3_, MeCN, reflux; (c) HCl, EtOAc; (d) ArB(OH)_2_, Pd_2_(dba)_3_, XantPhos, K_2_CO_3_, 1,4-dioxane, 100 °C; (e) 4 N HCl, 1,4-dioxane; (f) TFA, CH_2_Cl_2_ (g) ArB(OH)_2_, Pd(dppf)Cl_2_, K_2_CO_3_, 1,4-dioxane, H_2_O; (h) ArB(OH)_2_, Pd(PPh_3_)_4_, K_3_PO_4_, DMF.

**Scheme 5:**
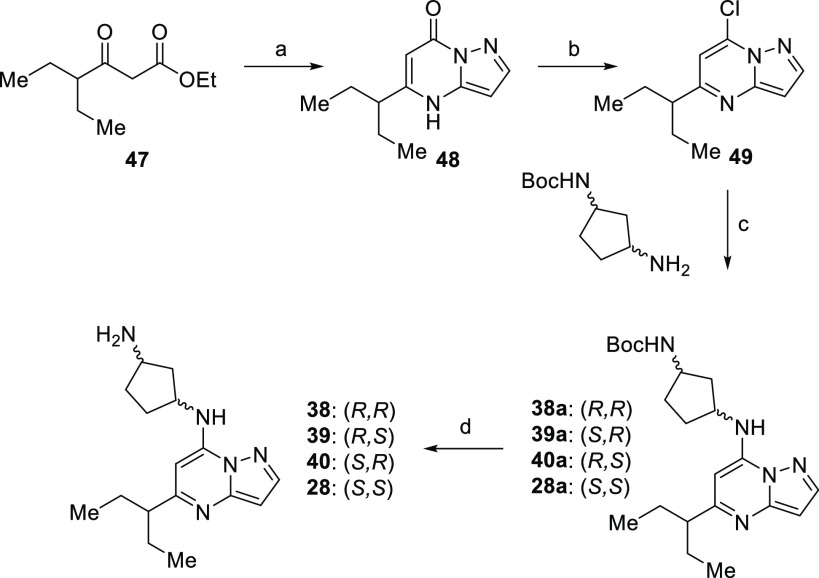
Synthesis of 1,3-Diaminocyclopentane Isomers via Chloro-pyrazolo[1,5-*a*]pyrimidine **49** Reagents and conditions: (a) 1*H*-pyrazol-5-amine, AcOH, reflux; (b) POCl_3_, pyridine, DMAP, MeCN, reflux; (c) K_2_CO_3_, MeCN, reflux; (d) HCl, 1,4-dioxane, 0 °C.

The syntheses of compounds **2**–**40** is described in Schemes 1–5 below and Schemes S3–S6 of the Supporting Information. The general approach focuses on modifications at three key carbon centers. The C-3 position (Figure 7) was functionalized through oxidative halogenation and potential further elaboration using Suzuki coupling chemistry. As a means to conserve the amino-pyrazolopyrimidine hinge binding motif, nucleophilic aromatic substitution of substituted primary amines with 5,7-dichloro-pyrazolo[1,5-*a*]pyrimidine gave C-7 substituted 5-chloro-pyrazolopyrimidines. Installation of the aryl R_1_ groups on the C-5 position also employed a Suzuki coupling of aryl boronic acids with 5-chloro-pyrazolo[1,5-*a*]pyrimidine intermediates. Alternatively, when hydrophobic moieties at the C-5 position were alkyl, a Masamune reaction accessed requisite β-ketoesters for a subsequent condensation of amino-pyrazole and deoxychlorination to deliver the 7-chloro-pyrazolo[1,5-*a*]pyrimidine intermediates. These compounds were then subjected to nucleophilic aromatic substitution with primary amines. Depending on the nature of each compound, deprotection and further amidation would deliver final targets.

Compounds **2–8**, **10**, **11**, **13**, and **16** (Scheme 1) commenced from a condensation of amino-pyrazole with ethyl 4-methyl-3-oxopentanoate (**41**) (see Supporting Information for synthesis) to create intermediate pyrazolo[1,5-*a*]pyrimidine-7(4*H*)-one (**42**). Subsequent deoxychlorination staged intermediate (**43**) for a parallel medicinal chemistry effort focused on a four-membered-ring series installed via nucleophilic aromatic substitution. Compounds **2**, **5–7**, **11**, and **16** were delivered directly. Unmasking of compounds **3** and **8** occurred after the Boc-deprotection of their precursor intermediates **3a** and **8a**, respectively. Reduction of **3a** and **8a** with LiAlH_4_ gave methylated analogues **4** and **10**. **13a** was converted to the carboxamide (**13**) using the Ghaffar–Parkins catalyst.

Compound **8** was then leveraged to access elaborated pendant amines (Scheme 2). Direct acylation of **8** delivered the amide **12**. Methyl-urea compound **14** was created by treating **8** with *S*-methyl methylaminoethanethioate. Construction of tool compound **15** arose via exposure of **8** with diphenyl *N*-cyanocarbonimidate to build the intermediate phenyl-isourea (not shown), which was subsequently treated with methyl amine. Direct treatment of **8** with methylsulfonyl chloride gave sulfonamide **17**.

The synthesis of five- and six-membered-ring compounds (Scheme 3) utilized a similar approach starting with chloro-pyrazolo[1,5-*a*]pyrimidine intermediate **43** to construct compounds **19**–**26**. Nucleophilic aromatic substitution delivered **25** and Boc-protected intermediates **18a–20a**, **22a–24a**, and **26a** from their corresponding primary amine building blocks. Subsequent deprotection with hydrochloric acid in dioxane provided the targeted analogues **18–20**, **22**, **23**, **24b**, and **26** in a rapid fashion. Furthermore, creation of methyl urea compound **24** arose from treatment of **24b** with *S*-methyl *N*-methylcarbamothioate.

An alternative synthetic strategy was employed (Scheme 4) when the proposed analogues contained an aryl group at position 5 of the pyrazolo[1,5-*a*]pyrimidine core (compounds **32**–**37**). Exploration of various aryl SAR trends started with a nucleophilic aromatic substitution of commercially available 5,7-dichloro-pyrazolo[1,5-*a*]pyrimidine (**44**) material with *tert*-butyl ((1*S,*3*S*)-3-aminocyclopentyl)carbamate to give compound **45**. Subsequent Suzuki–Miyaura coupling with a targeted grouping of aryl boronic acids gave penultimate compounds **32a** and **34a–37a**. Deprotection then accessed final targets **32** and **34–37**. In the case of 5-fluoro-2-methylphenyl substitution (**33**), the deprotection occurred prior to the coupling of the corresponding aryl boronic acid with intermediate **46**.

Stereoisomers **28** and **38–40** (Scheme 5) were made starting from a condensation of amino-pyrazole with ethyl 4-ethyl-3-oxohexanoate (**47**) to create intermediate pyrazolo[1,5-*a*]pyrimidin-7-(4*H*)-one (**48**) similar to what was described in Scheme 1. Deoxychlorination of **48** gave compound **49**, a compound positioned for subsequent nucleophilic aromatic substitution by the four possible stereoisomers of *N*-Boc-diamino-cyclopentane. Deprotection of intermediate amine-protected analogues **28a** and **38a–40a** delivered targeted stereoisomers **28** and **38–40**.

### General Chemistry

All reactions were carried out under an inert gas atmosphere (nitrogen or argon). Commercial reagents and anhydrous solvents were used without further purification. Removal of volatile organic solvents was performed by rotary evaporation, and residual trace solvents were removed via a high-vacuum manifold. Flash chromatography was performed on Combiflash Next Gen 100 from Teledyne ISCO using Agela Claricep 4–40g Si C-series silica gel 230–400 mesh cartridges. All reported yields are isolated yields. Preparative reversed-phase high-pressure liquid chromatography (prep-HPLC) was performed using an Agilent 1200 series instrument equipped with a Waters XBridge C18 OBD Prep Column (10 μm, 150 mm × 50 mm i.d.), eluting with a binary solvent of H_2_O and acetonitrile (ACN) using gradient elution (15–45% acetonitrile in a 10 mM ammonium bicarbonate solution in water, 8 min gradient), and detected via UV 220 nm. All assayed compounds were of ≥95% purity as determined by HPLC. UV detection at 220 nm was performed using an Agilent 1200 series with diode array detector (DAD) equipped with one of the following three columns: Phenomenex Gemini NX-C18 (3 μm, 100 mm × 4.6 mm i.d.), Agilent Zorbax Extend-C18 (5 μm, 150 mm × 4.6 mm i.d.), or Phenomenex Luna Omega Polar C18 (3 μm, 100 mm × 4.6 mm i.d.). A general method was employed: mobile phase, A = ACN, B = 10 mM NH_4_OAc in H_2_O; gradient, 5–95% A (0.0–18 min); flow rate = 1.0 mL/min; inj. vol. = 2.0 μL. Low-resolution liquid chromatography mass spectra (LCMS) were determined via a low-resolution electrospray ionization (ESI) source using any one of the following instruments and column combinations: Agilent 1260 Infinity II UPLC attached with Agilent 6110B single quad mass detector using a YMC Triart C18 column (3 μm, 33 mm × 2.1 mm); Waters Acquity UPLC attached with Waters ZQ SQ mass detector using a YMC Triart C18 column (3 μm, 33 mm × 2.1 mm); or Agilent 1260 Infinity II UPLC attached with Agilent 6110B single quad mass detector using a Phenomenex Kinetex C18 column (5 μm, 50 mm × 2.1 mm i.d.). High-resolution mass spectra (HRMS) were obtained on a Shimadzu Prominence HPLC coupled with an Applied Biosystems API2000/2000 Trap mass detector configured with an ESI source using a Waters Xbridge C18/Agilent Zorbax C18 column (5 μm, 50 mm × 4.6 mm i.d.). ^1^H NMR spectra were obtained on either a Bruker 400 MHz Ultra shield or Bruker 400 MHz Ascend spectrometer equipped with an Avance Neo, Avance III, or Avance III-HD console. Chemical shifts (δ) are reported in parts per million (ppm) relative to residual undeuterated solvent as an internal reference. The following abbreviations were used to label peak multiplicities: *s* = singlet, *d* = doublet, *t* = triplet, *q* = quartet, *quin* = quintet; *dd* = doublet of doublets, *dt* = doublet of triplets, *dtd* = doublet of triplet of doublets, *m* = multiplet, *br* = broad.

#### 5-Isopropyl-4*H*-pyrazolo[1,5-*a*]pyrimidin-7-one (**42**)

A mixture of ethyl 4-methyl-3-oxo-pentanoate (900 mg, 5.69 mmol) and 1*H*-pyrazol-5-amine (473 mg, 5.69 mmol) in glacial acetic acid (10 mL) was heated under reflux for 3 h. The solvent was evaporated *in vacuo*, and the residue was treated with ethyl acetate and filtered to give compound **42** (650 mg, 3.67 mmol, 64.5% yield) as a yellow solid. LCMS (ESI) *m*/*z*: 177.8 [M+H]^+^.

#### 7-Chloro-5-isopropylpyrazolo[1,5-*a*]pyrimidine (**43**)

A stirred solution of **42** (100 mg, 0.560 mmol) in POCl_3_ (1.06 mL, 11.3 mmol) was heated to reflux for 2 h. The reaction mixture was cooled to rt, excess reagent was removed *in vacuo*, and the residue was treated with ice–water. The product was extracted from an aqueous mixture by DCM. The organic layer was separated, dried over anhyd. Na_2_SO_4_, and purified by Combiflash column chromatography (silica gel, 230–400 mesh), eluting with 10% ethyl acetate in hexanes to give compound **43** (65 mg, 0.332 mmol, 58.9% yield) as a light-yellow liquid. LCMS (ESI) *m*/*z*: 195.9 [M+H]^+^.

#### *tert*-Butyl ((1*R*,4*R*)-4-((5-Isopropylpyrazolo[1,5-*a*]pyrimidin-7-yl)amino)cyclohexyl)carbamate (**26a**)

A stirred solution of **43** (75 mg, 0.380 mmol), *tert*-butyl ((1*R*,4*R*)-4-aminocyclohexyl)carbamate (98.6 mg, 0.460 mmol), and K_2_CO_3_ (106 mg, 0.770 mmol) in MeCN (10 mL) was heated to reflux for 16 h. The reaction mixture was cooled and filtered. The filtrate was concentrated *in vacuo*, and the residue was purified by Combiflash column chromatography, eluting with 25% ethyl acetate in hexanes to give compound **26a** (100 mg, 0.268 mmol, 69.9% yield) as a white solid. LCMS (ESI) *m*/*z*: 374.2 [M+H]^+^.

#### (1*R*,4*R*)-*N*1-[4-[(5-Isopropylpyrazolo[1,5-*a*]pyrimidin-4-ium-7-yl)amino]cyclohexyl]ammonium dichloride (**26**)

To compound **26a** (80 mg, 0.210 mmol) was added 4 M HCl in dioxane (2 mL, 0.210 mmol), and the mixture was stirred at rt for 2 h. The reaction mixture was evaporated *in vacuo* to give compound **26** (64.8 mg, 0.18 mmol, 83.9% yield) as an off-white solid. ^1^H NMR (400 MHz, DMSO-*d*_6_) δ ppm 8.24 (1 H, s), 6.53 (2 H, d, *J* = 16.3 Hz), 4.06–3.86 (1 H, m), 3.25–2.84 (2 H, m), 2.11–1.86 (4 H, m), 1.70 (2 H, q, *J* = 12.0 Hz), 1.54 (2 H, d, *J* = 11.9 Hz), 1.31 (6 H, d, *J* = 6.8 Hz). LCMS (ESI) *m*/*z*: 274.2 [M+H]^+^.

#### *tert*-Butyl *N*-[(1*R*,3*S*)-3-[(5-Isopropylpyrazolo[1,5-*a*]pyrimidin-7-yl)amino]cyclopentyl]carbamate (**23a**)

A stirred solution of compound **43** (75 mg, 0.380 mmol), *tert*-butyl *N*-[(1*R*,3*S*)-3-aminocyclopentyl]carbamate (92.1 mg, 0.460 mmol), and K_2_CO_3_ (159 mg, 1.15 mmol) in MeCN (10 mL) was heated to reflux for 16 h. The reaction mixture was filtered, concentrated *in vacuo*, and purified by Combiflash column chromatography (silica gel, 230–400 mesh), eluting with 25% ethyl acetate in hexanes to give compound **23a** (95 mg, 0.264 mmol, 68.9% yield) as a clear sticky liquid. LCMS (ESI) *m*/*z*: 359.9 [M+H]^+^.

#### (1*S*,3*R*)-*N*3-(5-Isopropylpyrazolo[1,5-*a*]pyrimidin-7-yl)cyclopentane-1,3-diamine (**23**)

To **23a** (75 mg, 0.210 mmol) was added 4 M HCl in dioxane (2 mL, 0.210 mmol) at 0 °C and stirred at rt for 2 h. The reaction mixture was evaporated *in vacuo*, and the residue was dissolved in MeOH and passed through a PL-HCO3MP SPE 200 mg/6 mL cartridge, eluting with MeOH, to give compound **23** (30.8 mg, 0.116 mmol, 55.5% yield) as a light-yellow gum. ^1^H NMR (400 MHz, DMSO-*d*_6_) δ ppm 7.97 (1 H, s), 6.28 (1 H, s), 6.04 (1 H, s), 4.21–4.05 (1 H, m), 3.43–3.35 (1 H, m), 2.96–2.84 (1 H, m), 2.27–2.10 (1 H, m), 2.10–1.92 (1H, m), 1.89–1.70 (2 H, m), 1.59–1.41 (2 H, m), 1.20 (6 H, d, *J* = 6.8 Hz). LCMS (ESI) *m*/*z*: 259.8 [M+H]^+^.

#### *tert*-Butyl *N*-[(1*S*,3*R*)-3-[(5-Isopropylpyrazolo[1,5-*a*]pyrimidin-7-yl)amino]cyclopentyl]carbamate (**22a**)

A stirred solution of compound **43** (75 mg, 0.380 mmol), *tert*-butyl *N*-[(1*S*,3*R*)-3-aminocyclopentyl]carbamate (92.1 mg, 0.460 mmol), and K_2_CO_3_ (158 mg, 1.15 mmol) in MeCN (10 mL) was heated to reflux for 16 h. The reaction mixture was filtered, concentrated *in vacuo*, and purified by Combiflash column chromatography (silica gel, 230–400 mesh), eluting with 25% ethyl acetate in hexanes to give compound **22a** (95 mg,0.264 mmol, 68.9% yield) as a clear sticky liquid. LCMS (ESI) *m*/*z*: 360.0 [M+H]^+^.

#### (1*R*,3*S*)-*N*3-(5-Isopropylpyrazolo[1,5-*a*]pyrimidin-7-yl)cyclopentane-1,3-diamine (**22**)

To compound **22a** (80 mg, 0.220 mmol) was added 4 M HCl in dioxane (2.mL, 0.220 mmol) at 0 °C and stirred at rt for 2 h. The reaction mixture was evaporated *in vacuo*, and the residue was dissolved in MeOH and passed through a PL-HCO3 MP SPE 200 mg/6 mL cartridge, eluting with MeOH to give compound **22** (26.03 mg,0.098 mmol, 43.97% yield) as a light-yellow gum. ^1^H NMR (400 MHz, DMSO-*d*_6_) δ ppm 7.97 (1 H, d, *J* = 1.4 Hz), 6.28 (1 H, d, *J* = 2.2 Hz), 6.04 (1 H, s), 4.16–4.08 (1 H, m), 3.37 (1 H, t, *J* = 5.5 Hz), 2.96–2.84 (1 H, m), 2.25–2.13 (1 H, m), 2.08–1.92 (1 H, m), 1.90–1.72 (2 H, m), 1.58–1.40 (2 H, m), 1.21 (6 H, d, *J* = 6.8 Hz). LCMS (ESI) *m/z:* 259.8 [M+H]^+^.

#### (1*S*,3*S*)-*N*3-(5*S*-butylpyrazolo[1,5-*a*]pyrimidin-7-yl)cyclopentane-1,3-diamine (**27**)

To compound **27e** (105 mg, 0.280 mmol; see Supporting Information for synthesis) was added 4 M HCl in dioxane (2 mL, 0.280 mmol), and the mixture was stirred at rt for 2 h. The reaction mixture was evaporated *in vacuo*, and the residue was dissolved in MeOH and passed through PL-HCO3 MP SPE 200 mg/6 mL cartridge, eluting with MeOH to give compound **27** (20.6 mg,0.075 mmol, 26.7% yield) as a light-yellow gum. ^1^H NMR (400 MHz, DMSO-*d*_6_) δ ppm 7.99 (1 H, d, *J* = 2.0 Hz), 7.44 (1 H, d, *J* = 6.9 Hz), 6.30 (1 H, d, *J* = 2.0 Hz), 6.00 (1 H, s), 4.24 (1 H, q, *J* = 7.2 Hz), 3.50–3.39 (1 H, m), 2.76–2.59 (1 H, m), 2.48–2.26 (2 H, m), 2.28–2.14 (1 H, m), 1.99–1.83 (2 H, m), 1.84–1.61 (3 H, m), 1.62–1.47 (1 H, m), 1.41–1.25 (1 H, m), 1.21 (3 H, d, *J* = 6.9 Hz), 0.82 (3 H, t, *J* = 7.3 Hz). LCMS (ESI) *m*/*z*: 273.8 [M+H]^+^.

#### *tert*-Butyl ((1*r*,3*r*)-3-((5-Isopropylpyrazolo[1,5-*a*]pyrimidin-7-yl)amino)cyclobutyl)carbamate (**8a**)

A stirred solution of compound **43** (75 mg, 0.380 mmol), *tert*-butyl ((1*r*,3*r*)-3-aminocyclobutyl)carbamate (85.7 mg, 0.460 mmol), and K_2_CO_3_ (158 mg, 1.15 mmol) in MeCN (10 mL) was heated to reflux for 16 h. The reaction mixture was filtered and concentrated *in vacuo*, and the residue was purified by Combiflash column chromatography (silica gel, 230–400 mesh), eluting with 25% ethyl acetate in hexanes to give compound **8a** (90 mg,0.261 mmol, 68.0% yield) as a white solid. LCMS (ESI) *m*/*z*: 346.1 [M+H]^+^.

#### (1*r*,3*r*)-*N*1-(5-Isopropylpyrazolo[1,5-*a*]pyrimidin-7-yl)cyclobutane-1,3-diamine (**8**)

To compound **8a** (75.mg, 0.220 mmol) was added 4 M HCl in dioxane (2.mL, 0.220 mmol) at 0 °C and stirred at rt for 2 h. The reaction mixture was monitored by TLC (100% ethyl acetate, product *R*_f_ = 0.1, and SM *R*_f_ = 0.8). The reaction mixture was evaporated *in vacuo*, and the residue was dissolved in MeOH and passed through by PL-HCO3 MP SPE 200 mg/6 mL cartridge, eluting with MeOH, to give compound **8** (32.01 mg,0.125 mmol, 57.62% yield) as a light-yellow gum. ^1^H NMR (400 MHz, DMSO-*d*_6_) δ 8.00 (1 H, d, *J* = 2.2 Hz), 6.30 (1 H, d, *J* = 1.7 Hz), 5.82 (1 H, s), 4.32–4.24 (1 H, m), 3.57–3.42 (1 H, m), 2.95–2.83 (1 H, m), 2.43–2.31 (2 H, m), 2.19–2.10 (2 H, m), 1.20 (6 H, d, *J* = 6.8 Hz). LCMS (ESI) *m*/*z*: 246.4 [M+H]^+^.

#### *tert*-Butyl *N*-[(1*S*,3*S*)-3-[(5-isopropylpyrazolo[1,5-*a*]pyrimidin-7-yl)amino]cyclopentyl]carbamate (**20a**)

A stirred solution of compound **43** (75 mg, 0.380 mmol), *tert*-butyl ((1*S*,3*S*)-3-aminocyclopentyl)carbamate (92.13 mg, 0.460 mmol), and K_2_CO_3_ (159 mg, 1.15 mmol) in MeCN (10 mL) was heated to reflux for 16 h. The reaction mixture was filtered, concentrated *in vacuo*, and purified by Combiflash column chromatography (silica gel, 230–400 mesh), eluting with 25% ethyl acetate in hexanes to give compound **20a** (100 mg,0.278 mmol, 72.6% yield) as a white solid. LCMS (ESI) *m*/*z*: 360.1 [M+H]^+^.

#### (1*S*,3*S*)-*N*3-(5-Isopropylpyrazolo[1,5-*a*]pyrimidin-7-yl)cyclopentane-1,3-diamine (**20**)

To compound **20a** (80 mg, 0.220 mmol) was added 4 M HCl in dioxane (2.mL, 0.220 mmol) at 0 °C, and the mixture was stirred at rt for 2 h. The reaction mixture was evaporated *in vacuo*, and the residue was dissolved in MeOH and passed through a PL-HCO3 MP SPE 200 mg/6 mL cartridge, eluting with MeOH to give compound **20** (31.02 mg, 0.116 mmol, 52.14% yield) as a light-yellow gum. ^1^H NMR (400 MHz, DMSO-*d*_6_) δ ppm 7.98 (1 H, s), 6.32–6.26 (1 H, m), 6.03 (1 H, s), 4.43–4.07 (1 H, m), 3.52–3.34 (1 H, m), 2.95–2.86 (1 H, m), 2.29–2.11 (1 H, m), 2.08–1.75 (3 H, m), 1.71–1.56 (1 H, m), 1.41–1.28 (1 H, m), 1.21 (6 H, d, *J* = 6.9 Hz). LCMS (ESI) *m*/*z*: 260.2 [M+H]^+^.

#### *N*-Cyclobutyl-5-isopropylpyrazolo[1,5-*a*]pyrimidin-7-amine (**2**)

A stirred solution of compound **43** (100 mg, 0.480 mmol), cyclobutanamine (40.7 mg, 0.570 mmol), and K_2_CO_3_ (197 mg, 1.43 mmol) in MeCN (10 mL) was heated to reflux for 16 h. The reaction mixture was concentrated under reduced pressure, and then water (50 mL) was added and extracted with ethyl acetate (20 mL × 2). The combined organic layers were dried under anhyd. Na_2_SO_4_ and concentrated *in vacuo*. The crude product was purified by Combiflash column chromatography (silica, 230–400 mesh), eluting with 20% ethyl acetate in hexanes to give compound **2** (50 mg, 0.213 mmol, 44.8% yield) as an off-white solid. ^1^H NMR (400 MHz, DMSO-*d*_6_) δ ppm 8.00 (1 H, d, *J* = 2.0 Hz), 7.87 (1 H, d, *J* = 7.3 Hz), 6.30 (1 H, d, *J* = 2.2 Hz), 5.98 (1 H, s), 4.29–4.14 (1 H, m), 2.96–2.87 (1 H, m), 2.43–2.31 (2 H, m), 2.28–2.13 (2 H, m), 1.86–1.63 (2 H, m), 1.22 (6 H, d, *J* = 6.9 Hz). LCMS (ESI) *m*/*z*: 231.0 [M+H]^+^.

#### (1*S*,3*S*)-*N*3-(5-Isobutylpyrazolo[1,5-*a*]pyrimidin-7-yl)cyclopentane-1,3-diamine (**30**)

To compound **30e** (120 mg, 0.320 mmol; see Supporting Information for synthesis) was added 4 M HCl in dioxane (2 mL, 0.320 mmol) at 0 °C and stirred at rt for 2 h. The reaction mixture was evaporated *in vacuo*. The residue was dissolved in MeOH and passed through PL-HCO3MP SPE 500 mg/6 mL cartridge, eluting with MeOH to give compound **30** (69.1 mg,0.250 mmol, 77.9% yield) as a light-brown sticky gum. ^1^H NMR (400 MHz, CD_3_OD) δ ppm 7.96 (1 H, d, *J* = 2.0 Hz), 6.30 (1 H, d, *J* = 2.0 Hz), 6.03 (1 H, s), 4.39–4.27 (1 H, m), 3.75–3.63 (1 H, m), 2.58 (2 H, d, *J* = 7.3 Hz), 2.46–2.32 (1 H, m), 2.32–2.18 (1 H, m), 2.20–1.99 (3 H, m), 1.89–1.74 (1 H, m), 1.68–1.54 (1 H, m), 0.97 (6 H, d, *J* = 6.6 Hz). LCMS (ESI) *m*/*z*: 273.7 [M+H]^+^.

#### (1*S*,3*S*)-*N*3-(3-Chloro-5-isopropyl-pyrazolo[1,5-*a*]pyrimidin-7-yl)cyclopentane-1,3-diamine (**21**)

To **21a** (85 mg, 0.220 mmol; see Supporting Information for synthesis) was added 4 M HCl in dioxane (2 mL, 0.220 mmol) at 0 °C and stirred at rt for 2 h. The reaction mixture was evaporated *in vacuo*. The residue was dissolved in MeOH and passed through PL-HCO3 MP SPE 500 mg/6 mL cartridge, eluting with MeOH to give compound **21** (27.0 mg, 0.0907 mmol, 42.0% yield) as an off-white sticky solid. ^1^H NMR (400 MHz, CD_3_OD) δ ppm 7.95 (1 H, s), 6.09 (1 H, s), 4.39–4.27 (1 H, m), 3.62–3.48 (1 H, m), 3.08–2.94 (1 H, m), 2.43–2.30 (1 H, m), 2.23–2.10 (1 H, m), 2.08–1.89 (2 H, m), 1.81–1.67 (1 H, m), 1.57–1.43 (1 H, m), 1.33 (6 H, d, *J* = 6.9 Hz). LCMS (ESI) *m*/*z*: 293.8 [M+H]^+^.

#### (1*r*,3*r*)-*N*1-(3-Chloro-5-isopropyl-pyrazolo[1,5-*a*]pyrimidin-7-yl)cyclobutane-1,3-diamine (**9**)

To compound **9a** (65 mg, 0.170 mmol; see Supporting Information for synthesis) was added 4 M HCl in dioxane (2 mL, 0.170 mmol) at 0 °C and stirred at rt for 2 h. The reaction mixture was evaporated *in vacuo*. The residue was dissolved in MeOH and passed through PL-HCO3 MP SPE 500 mg/6 mL cartridge, eluting with MeOH to give compound **9** (26.1 mg,0.093 mmol, 54.2% yield) as an off-white sticky solid. ^1^H NMR (400 MHz, CD_3_OD) δ ppm 7.97 (1 H, s), 5.89 (1 H, s), 4.44–4.32 (1 H, m), 3.77–3.66 (1 H, m), 3.08–2.93 (1 H, m), 2.55–2.43 (2 H, m), 2.41–2.29 (2 H, m), 1.31 (6 H, d, *J* = 6.9 Hz). LCMS (ESI) *m*/*z*: 279.9 [M+H]^+^.

#### 5-Isopropyl-*N*-(oxetan-3-yl)pyrazolo[1,5-*a*]pyrimidin-7-amine (**5**)

A stirred solution of compound **43** (100 mg, 0.480 mmol), oxetan-3-amine (41.8 mg, 0.570 mmol), and K_2_CO_3_ (197 mg, 1.43 mmol) in MeCN (10 mL) was heated to reflux for 16 h. The reaction mixture was concentrated under reduced pressure, and then water (50 mL) was added and extracted with ethyl acetate (20 mL × 2). The combined organic layers were dried under anhyd. Na_2_SO_4_ and concentrated *in vacuo*. The crude product was purified by Combiflash column chromatography (silica, 230–400 mesh), eluting with 20% ethyl acetate in hexanes to give compound **5** (90 mg, 0.384 mmol, 80.6% yield) as an off-white solid. ^1^H NMR (400 MHz, DMSO-*d*_6_) δ ppm 8.44 (1 H, d, *J* = 6.2 Hz), 8.05 (1 H, d, *J* = 1.7 Hz), 6.34 (1 H, d, *J* = 1.8 Hz), 5.90 (1 H, s), 5.00–4.88 (1 H, m), 4.86 (2 H, t, *J* = 6.9 Hz), 4.74 (2 H, t, *J* = 6.1 Hz), 2.98–2.83 (1 H, m), 1.22 (6 H, d, *J* = 6.8 Hz). LCMS (ESI) *m*/*z*: 233.0 [M+H]^+^.

#### *tert*-Butyl (3*S*)-3-[(5-isopropylpyrazolo[1,5-*a*]pyrimidin-7-yl)amino]pyrrolidine-1-carboxylate (**18a**)

A stirred solution of compound **43** (200 mg, 1.02 mmol), *tert*-butyl (3*S*)-3-aminopyrrolidine-1-carboxylate (228 mg, 1.23 mmol), and K_2_CO_3_ (423 mg, 3.07 mmol) in MeCN (20 mL) was heated to reflux for 16 h. The reaction mixture was concentrated under reduced pressure, and then water (50 mL) was added and extracted with ethyl acetate (20 mL × 2). The combined organic layers were dried under anhyd. Na_2_SO_4_ and concentrated *in vacuo*. The crude product was purified by Combiflash column chromatography (silica, 230–400 mesh), eluting with 20% ethyl acetate in hexanes to give compound **18a** (240 mg, 0.695 mmol, 68% yield) as a pale-yellow sticky compound. LCMS (ESI) *m*/*z*: 345.8 [M+H]^+^.

#### 5-Isopropyl-*N*-[(3*S*)-pyrrolidin-3-yl]pyrazolo[1,5-*a*]pyrimidin-7-amine Hydrochloride (**18**)

To compound **18a** (250 mg, 0.690 mmol) was added 4 M HCl in dioxane (3 mL, 0.690 mmol), and the mixture was stirred at rt for 2 h. The reaction mixture was evaporated *in vacuo*, and the resulting solid was triturated with pentane (3 × 5 mL) and decanted. The solid obtained was dried *in vacuo* to give compound **18** (160 mg, 0.565 mmol, 81.6% yield) as a pale-yellow solid. ^1^H NMR (400 MHz, DMSO-*d*_6_) δ ppm 9.80–9.44 (1 H, m), 9.35–8.98 (1 H, m), 8.30 (1 H, s), 6.57 (2 H, s), 4.94–4.66 (1 H, m), 3.61–3.37 (4 H, m), 3.30–3.25 (1 H, m), 3.20–3.12 (1 H, m), 2.42–2.28 (1 H, m), 2.23–2.07 (1 H, m), 1.35 (6 H, d, *J* = 6.9 Hz). LCMS (ESI) *m*/*z*: 246.4 [M+H]^+^.

#### *tert*-Butyl (3*R*)-3-[(5-isopropylpyrazolo[1,5-*a*]pyrimidin-7-yl)amino]pyrrolidine-1-carboxylate (**19a**)

A stirred solution of compound **43** (200 mg, 1.02 mmol), *tert*-butyl (3*R*)-3-aminopyrrolidine-1-carboxylate (228 mg, 1.23 mmol), and K_2_CO_3_ (423 mg, 3.07 mmol) in MeCN (20 mL) was heated to reflux for 16 h. The reaction mixture was concentrated under reduced pressure, and then water (50 mL) was added and extracted with ethyl acetate (20 mL × 2). The combined organic layers were dried under anhyd. Na_2_SO_4_ and concentrated *in vacuo*. The crude mixture was purified by Combiflash column chromatography (silica, 230–400 mesh), eluting with 20% ethyl acetate in hexanes to give compound **19a** (250 mg, 0.724 mmol, 70.8% yield) as a pale-yellow sticky liquid. LCMS (ESI) *m*/*z*: 346.4 [M+H]^+^.

#### *tert*-Butyl (3*R*)-3-[(5-isopropylpyrazolo[1,5-*a*]pyrimidin-7-yl)amino]pyrrolidine-1-carboxylate (**19**)

To compound **19a** (248 mg, 0.720 mmol) was added 4 M HCl in dioxane (3 mL, 12 mmol), and the mixture was stirred at rt for 2 h. The reaction mixture was evaporated *in vacuo*, and the resulting solid was triturated with pentane (3 × 5 mL) and decanted. The remaining solid was dried *in vacuo* to give compound **19** (150 mg, 0.530 mmol, 73.87% yield) as a pale-yellow solid. ^1^H NMR (400 MHz, DMSO-*d*_6_) δ ppm 8.29 (1 H, s), 6.59–6.51 (2 H, m), 4.91–4.72 (1 H, m), 3.62–3.53 (1 H, m), 3.49–3.37 (2 H, m), 3.34–3.23 (1 H, m), 3.17–3.05 (1 H, m), 2.46–2.32 (1 H, m), 2.22–2.08 (1 H, m), 1.33 (6 H, d, *J* = 6.9 Hz). LCMS (ESI) *m*/*z*: 245.9 [M+H]^+^.

#### *tert*-Butyl 3-[(5-Isopropylpyrazolo[1,5-*a*]pyrimidin-7-yl)amino]azetidine-1-carboxylate (**3a**)

A stirred solution of compound **43** (200 mg, 1.02 mmol), *tert*-butyl 3-aminoazetidine-1-carboxylate (211 mg, 1.23 mmol), and K_2_CO_3_ (423 mg, 3.07 mmol) in MeCN (20 mL) was heated to reflux for 16 h. The reaction mixture was concentrated under reduced pressure, and then water (50 mL) was added and extracted with ethyl acetate (30 mL × 2). The combined organic layers were dried under anhyd. Na_2_SO_4_ and concentrated *in vacuo*. The crude material was purified by Combiflash column chromatography (silica, 230–400 mesh), eluting with 20% ethyl acetate in hexanes to give compound **3a** (240 mg, 0.724 mmol, 70.8% yield) as a pale-yellow sticky compound. LCMS (ESI) *m*/*z*: 332.3 [M+H]^+^.

#### *N*-(Azetidin-3-yl)-5-isopropylpyrazolo[1,5-*a*]pyrimidin-7-amine (**3**)

To compound **3a** (240 mg, 0.690 mmol) was added 4 M HCl in dioxane (3 mL, 0.690 mmol), and the mixture was stirred at rt for 2 h. The reaction mixture was evaporated *in vacuo* and triturated with pentane (3 × 5 mL). The crude solid was purified by prep-HPLC followed by lyophilization to give compound **3** (55 mg, 0.236 mmol, 34.2% yield) as an off-white solid. ^1^H NMR (400 MHz, DMSO-*d*_6_) δ ppm 1.26 (6 H, d, *J* = 6.9 Hz), 2.90–2.97 (1 H, m), 3.64–3.73 (2 H, m), 3.80 (2 H, t, *J* = 7.6 Hz), 4.57 (1 H, q, *J* = 7.0 Hz), 5.91 (1 H, s), 6.30 (1 H, s), 7.5–7.61 (1 H, m), 7.98 (1 H, s). LCMS (ESI) *m*/*z*: 231.9 [M+H]^+^.

#### 5-Isopropyl-*N*-(1-methylazetidin-3-yl)pyrazolo[1,5-*a*]pyrimidin-7-amine (**4**)

A solution of compound **3a** (180 mg, 0.540 mmol) in THF (15 mL) was cooled to 0 °C. Then LiAlH_4_ (61.8 mg, 1.63 mmol) was added portion-wise. After the complete addition, the reaction mixture was stirred at 80 °C for 4 h. The reaction mixture was quenched carefully with an aq. Na_2_SO_4_ solution and extracted with ethyl acetate (3 × 40 mL). The combined organic layers were dried under anhyd. Na_2_SO_4_ and concentrated *in vacuo* to give the crude product. This crude product was purified by prep-HPLC followed by lyophilization to give compound **4** (50 mg, 0.201 mmol, 37.0% yield) as a white, sticky solid. ^1^H NMR (400 MHz, DMSO-*d*_6_) δ ppm 1.22 (6 H, d, *J* = 6.9 Hz), 2.27 (3 H, s), 2.84–2.97 (1 H, m), 3.11 (2 H, t, *J* = 6.9 Hz), 3.69 (2 H, t, *J* = 6.7 Hz), 4.22–4.35 (1 H, m), 5.98 (1 H, s), 6.32 (1 H, s), 8.02–8.05 (2 H, m). LCMS (ESI) *m*/*z*: 246.1 [M+H]^+^.

#### (1*r*,3*r*)-3-[(5-Isopropylpyrazolo[1,5-*a*]pyrimidin-7-yl)amino]cyclobutanol (**6**)

A stirred solution of compound **43** (100 mg, 0.510 mmol), (1*r*,3*r*)-3-aminocyclobutan-1-ol hydrochloride (75.8 mg, 0.610 mmol), and K_2_CO_3_ (212 mg, 1.53 mmol) in MeCN (10 mL) was heated to reflux for 16 h. The reaction mixture was concentrated under reduced pressure, and then water (50 mL) was added and extracted with ethyl acetate (30 mL × 2). The combined organic layers were dried under anhyd. Na_2_SO_4_ and concentrated *in vacuo*. The crude material was purified by Combiflash column chromatography (silica, 230–400 mesh), eluting with 20% ethyl acetate in hexanes to give the crude product. The crude product was re-purified by prep-HPLC followed by lyophilization to give compound **6** (35 mg, 0.142 mmol, 27.8% yield) as a white solid. ^1^H NMR (400 MHz, DMSO-*d*_6_) δ ppm 1.25–1.28 (6 H, m), 2.31–2.38 (2 H, m), 2.45–2.47 (2 H, m), 2.91–2.93 (1 H, m), 4.23–4.33 (1 H, m), 4.37–4.45 (1 H, m), 4.77 (1 H, d, *J* = 5.2 Hz), 5.82 (1 H, s), 6.26–6.32 (1 H, m), 7.31–7.34 (1 H, m), 7.95–7.98 (1 H, m). LCMS (ESI) *m*/*z*: 247.2 [M+H]^+^.

#### (1*S*,3*S*)-3-[(5-Isopropylpyrazolo[1,5-*a*]pyrimidin-7-yl)amino]cyclopentanol (**25**)

A stirred solution of compound **43** (100 mg, 0.510 mmol), (1*S*,3*S*)-3-aminocyclopentanol hydrochloride (84.4 mg, 0.610 mmol), and K_2_CO_3_ (212 mg, 1.53 mmol) in MeCN (10 mL) was heated at 80 °C for 16 h. The reaction mixture was concentrated under reduced pressure, and then water (50 mL) was added and extracted with ethyl acetate (30 mL × 2). The combined organic layers were dried under anhyd. Na_2_SO_4_ and concentrated *in vacuo*. The crude material was purified by Combiflash column chromatography (silica, 230–400 mesh), eluting with 20% ethyl acetate in hexanes to give compound **25** (50 mg, 0.188 mmol, 36.7% yield) as a colorless sticky liquid. ^1^H NMR (400 MHz, DMSO-*d*_6_) δ ppm 1.24 (6 H, d, *J* = 6.8 Hz), 1.48–1.56 (1 H, m), 1.59–1.69 (1 H, m), 1.84–2.04 (3 H, m), 2.15–2.26 (1 H, m), 2.87–2.99 (1 H, m), 4.19–4.30 (2 H, m), 4.57–4.62 (1 H, m), 6.03 (1 H, s), 6.29–6.32 (1 H, m), 7.50 (1 H, d, *J* = 7.9 Hz), 7.99–8.00 (1 H, m). LCMS (ESI) *m*/*z*: 261.3 [M+H]^+^.

#### (1*r*,3*r*)-*N*1-(5-Isopropylpyrazolo[1,5-*a*]pyrimidin-7-yl)-*N*3-methylcyclobutane-1,3-diamine (**10**)

A solution of compound **8a** (100 mg, 0.290 mmol) in THF (10 mL) was cooled to 0 °C, and LiAlH_4_ (32.9 mg, 0.870 mmol) was added portion-wise. After complete addition, the reaction mixture was stirred at 80 °C for 16 h. Then the reaction mixture was quenched carefully with cold aq. Na_2_SO_4_ solution and extracted with ethyl acetate (3 x× 40 mL). The combined organic layers were dried over anhyd. Na_2_SO_4_ and concentrated *in vacuo* to give a crude material. This crude material was purified by prep-HPLC followed by lyophilization to give compound **10** (30 mg, 0.115 mmol, 39.9% yield) as an off-white solid. ^1^H NMR (400 MHz, DMSO-*d*_6_) δ ppm 1.26 (6 H, d, *J* = 6.9 Hz), 1.89–1.95 (1 H, br), 2.21–2.28 (5 H, m), 2.29–2.40 (2 H, m), 2.91–2.93 (1 H, m), 3.27–3.36 (1 H, m), 4.28 (1 H, q, *J* = 6.7 Hz), 5.83 (1 H, s), 6.29 (1 H, s), 7.34 (1 H, br), 7.97 (1 H, s). LCMS (ESI) *m*/*z*: 260.0 [M+H]^+^.

#### (1*r*,3*r*)-*N*-[3-[(5-Isopropylpyrazolo[1,5-*a*]pyrimidin-7-yl)amino]cyclobutyl]methanesulfonamide (**17**)

To a stirred solution of compound **8** (200 mg, 0.630 mmol) in DCM (5 mL) was added NaHCO_3_ (264 mg, 3.14 mmol), stirred for 30 min, extracted with DCM, and then washed with water and brine to give the free amine. To the free amine in DCM (5 mL) were added DIPEA (0.16 mL, 0.940 mmol) and methanesulfonyl chloride (0.07 mL, 0.940 mmol) at 0 °C, and the mixture was stirred for 6 h. The reaction mixture was washed with water and brine and dried over anhyd. Na_2_SO_4_. The crude product was purified by prep HPLC to give compound **17** (73.6 mg, 0.22 mmol, 36.1% yield) as a white solid. ^1^H NMR (400 MHz, CDCl_3_) δ ppm 1.30 (6 H, d, *J* = 6.9 Hz), 2.51–2.68 (4 H, m), 2.89–3.05 (4 H, m), 4.24–4.30 (2 H, m), 4.87 (1 H, d, *J* = 7.4 Hz), 5.64 (1 H, s), 6.40 (1 H, d, *J* = 5.4 Hz), 6.44 (1 H, s), 7.95 (1 H, s). LCMS (ESI) *m*/*z*: 324.0 [M+H]^+^.

#### (1*r*,3*r*)-[3-[(5-Isopropylpyrazolo[1,5-*a*]pyrimidin-7-yl)amino]cyclobutyl]methanol (**11**)

A stirred solution of compound **43** (100 mg, 0.510 mmol), (1*r*,3*r*)- (3-aminocyclobutyl)methanol hydrochloride (84.4 mg, 0.610 mmol), and K_2_CO_3_ (212 mg, 1.53 mmol) in MeCN (10 mL) was heated to reflux for 16 h. The reaction mixture was concentrated under reduced pressure, and then water (25 mL) was added and extracted with ethyl acetate (30 mL × 2). The combined organic layers were dried under anhyd. Na_2_SO_4_ and concentrated *in vacuo*. The crude material was purified by Combiflash column chromatography (silica, 230–400 mesh), eluting with 20% ethyl acetate in hexanes to give compound **11** (90 mg, 0.34 mmol, 66.4% yield) as a sticky liquid. ^1^H NMR (400 MHz, DMSO-*d*_6_) δ ppm 1.26 (6 H, d, *J* = 6.9 Hz), 2.24–2.34 (4 H, m), 2.41–2.43 (1 H, m), 2.91–2.93 (1 H, m), 3.56 (2 H, t, *J* = 5.7 Hz), 4.20–4.24 (1 H, m), 4.28 (1 H, br), 5.85 (1 H, s), 6.29 (1 H, s), 7.32 (1 H, br), 7.97 (1 H, s). LCMS (ESI) *m*/*z*: 260.8 [M+H]^+^.

#### (1*r*,3*r*)-*N*-[3-[(5-Isopropylpyrazolo[1,5-*a*]pyrimidin-7-yl)amino]cyclobutyl]acetamide (**12**)

To a stirred solution of compound **8** (100 mg, 0.410 mmol) in pyridine (6 mL) was added acetic anhydride (0.05 mL, 0.490 mmol), and the mixture was stirred overnight. The reaction mixture was concentrated, partitioned between ethyl acetate and water, and extracted. The organic layers were washed with water and brine, dried over anhyd. Na_2_SO_4_, and evaporated *in vacuo* to give the crude product. The crude product was purified by prep-HPLC to give compound **12** (5 mg, 0.017 mmol, 4.2% yield) as an off-white solid. ^1^H NMR (400 MHz, CDCl_3_) δ ppm 7.94 (1 H, d, *J* = 2.0 Hz), 6.41 (1 H, d, *J* = 2.0 Hz), 5.63 (1 H, s), 4.54 (1 H, t, *J* = 6.8 Hz), 4.38–4.13 (1 H, m), 3.14–2.81 (1 H, m), 2.66–2.43 (3 H, m), 2.00 (3 H, s), 1.30 (6 H, d, *J* = 6.9 Hz). LCMS (ESI) *m*/*z*: 288.0 [M+H]^+^.

#### (1*r*,3*r*)-3-[(5-Isopropylpyrazolo[1,5-*a*]pyrimidin-7-yl)amino]cyclobutanecarbonitrile (**13a**)

A stirred solution of compound **43** (300 mg, 1.53 mmol), (1*r*,3*r*)-3-aminocyclobutanecarbonitrile hydrochloride (244 mg, 1.84 mmol), and K_2_CO_3_ (635 mg, 4.6 mmol) in MeCN (30 mL) was heated at reflux temperature for 16 h. The reaction mixture was concentrated *in vacuo*, and then water (50 mL) was added and extracted with ethyl acetate (20 mL × 2). The combined organic layers were dried under anhyd. Na_2_SO_4_ and concentrated *in vacuo*. The crude material was purified by Combiflash column chromatography (silica, 230–400 mesh), eluting with 20% ethyl acetate in hexanes to give compound **13a** (300 mg,1.175 mmol, 76.6% yield) as an off-white solid. LCMS (ESI) *m*/*z*: 256.1 [M+H]^+^.

#### (1*r*,3*r*)-3-[(5-Isopropylpyrazolo[1,5-*a*]pyrimidin-7-yl)amino]cyclobutanecarboxamide (**13**)

To a stirred solution of compound **13a** (70 mg, 0.270 mmol) in THF (2 mL) and water (0.01 mL, 0.550 mmol) was added hydrido(dimethylphosphinous acid-kP)[hydrogen bis(dimethylphosphinito-kP)]platinum(II) (Ghaffar–Parkins catalyst) (11.8 mg, 0.030 mmol) and stirred at 70 °C for 12 h. The reaction mixture was then diluted with water (20 mL) and extracted with ethyl acetate (25 mL). The combined ethyl acetate layers were washed with a water layer, dried over anhyd. Na_2_SO_4_, and concentrated *in vacuo*. The crude material was purified by Combiflash column chromatography (silica, 230–400 mesh), eluting with 10% MeOH–DCM to give compound **13** (35 mg, 0.127 mmol, 46.3% yield) as a white solid. ^1^H NMR (400 MHz, DMSO-*d*_6_) δ ppm 1.22 (6 H, d, *J* = 6.9 Hz), 2.36–2.48 (2 H, m), 2.52–2.54 (2 H, m), 2.86–3.01 (2 H, m), 4.22–4.36 (1 H, m), 5.85 (1 H, s), 6.29–6.35 (1 H, m), 6.81–6.87 (1 H, m), 7.29 (1 H, s), 7.94–8.05 (2 H, m). LCMS (ESI) *m*/*z*: 273.8 [M+H]^+^.

#### (1*r*,3*r*)-*N*1-(5-Isopropylpyrazolo[1,5-*a*]pyrimidin-7-yl)-*N*3-phenylcyclobutane-1,3-diamine (**16**)

A stirred solution of compound **43** (90 mg, 0.460 mmol), (1*r*,3*r*)-*N*1-phenylcyclobutane-1,3-diamine hydrochloride (110 mg, 0.550 mmol), and K_2_CO_3_ (190 mg, 1.38 mmol) in MeCN (9 mL) was heated to reflux for 16 h. The reaction mixture was concentrated under reduced pressure, and then water (50 mL) was added and extracted with ethyl acetate (30 mL × 2). The combined organic layers were dried under anhyd. Na_2_SO_4_ and concentrated *in vacuo*. The crude product was purified by Combiflash column chromatography (silica, 230–400 mesh), eluting with 30% ethyl acetate in hexanes to give compound **16** (50 mg, 0.153 mmol, 33.4% yield) as a pale-yellow solid. ^1^H NMR (400 MHz, DMSO-*d*_6_) δ ppm 1.22 (6 H, d, *J* = 6.9 Hz), 2.28–2.39 (2 H, m), 2.55–2.66 (2 H, m), 2.86–2.98 (1 H, m), 3.97 (1 H, d, *J* = 5.9 Hz), 4.27–4.37 (1 H, m), 5.87 (1 H, s), 5.98 (1 H, d, *J* = 5.8 Hz), 6.33 (1 H, s), 6.47–6.59 (3 H, m), 7.08 (2 H, t, *J* = 7.7 Hz), 8.00–8.09 (2 H, m). LCMS (ESI) *m*/*z*: 322.0 [M+H]^+^.

#### (1*r*,3*r*)-*N*-(3-Ethoxycyclobutyl)-5-isopropylpyrazolo[1,5-*a*]pyrimidin-7-amine (**7**)

A stirred solution of compound **43** (100 mg, 0.510 mmol), (1*r*,3*r*)-3-ethoxycyclobutanamine hydrochloride (93 mg, 0.610 mmol), and K_2_CO_3_ (212 mg, 1.53 mmol) in MeCN (10 mL) was heated to reflux for 16 h. The reaction mixture was then filtered and concentrated under reduced pressure. The crude product was purified by prep-HPLC to give compound **7** (36.5 mg, 0.133 mmol, 25.9% yield) as a colorless, sticky gum. ^1^H NMR (400 MHz, CDCl_3_) δ ppm 1.21–1.26 (3 H, m), 1.30 (6 H, d, *J* = 6.8 Hz), 2.34–2.41 (2 H, m), 2.49–2.61 (2 H, m), 2.91–3.04 (1 H, m), 3.44 (2 H, q, *J* = 7.0 Hz), 4.20–4.31 (2 H, m), 5.69 (1 H, s), 6.31 (1 H, d, *J* = 5.7 Hz), 6.42 (1 H, s), 7.94 (1 H, s). LCMS (ESI) *m*/*z*: 274.8 [M+H]^+^.

#### *tert*-Butyl *N*-[(1*R*,3*R*)-3-[(5-Isopropylpyrazolo[1,5-*a*]pyrimidin-7-yl)amino]cyclopentyl]carbamate (**24a**)

A stirred solution of compound **43** (0.35 g, 1.79 mmol), *tert*-butyl *N*-[(1*R*,3*R*)-3-aminocyclopentyl]carbamate (0.4 g, 2 mmol), and K_2_CO_3_ (0.78 g, 5.64 mmol) in MeCN (17.5 mL) was heated to reflux for 16 h. The reaction mixture was concentrated under reduced pressure, and then water (50 mL) was added and extracted with ethyl acetate (30 mL × 2). The combined organic layers were dried under anhyd. Na_2_SO_4_ and concentrated *in vacuo*. This crude material was purified by Combiflash column chromatography (silica, 230–400 mesh), eluting with 30% ethyl acetate in hexanes to give compound **24a** (450 mg, 1.25 mmol, 70.0% yield) as a white solid. LCMS (ESI) *m*/*z*: 360.0 [M+H]^+^.

#### (1*R*,3*R*)-*N*3-(5-Isopropylpyrazolo[1,5-*a*]pyrimidin-7-yl)cyclopentane-1,3-diamine (**24b**)

To a stirred solution of compound **24a** (0.45 g, 1.25 mmol) in dioxane (0.5 mL) at 0 °C was added 4 M HCl in dioxane (20 mL, 1.25 mmol) and stirred at 0 °C for 30 min. The reaction mixture was then stirred at rt for 6 h. After completion of the reaction, the reaction mixture was concentrated *in vacuo*, and the residue was diluted with CH_2_Cl_2_ (50 mL). The organic layer was washed with sat. NaHCO_3_ (10 mL) solution and brine (10 mL). The organic layer was then dried over anhyd. Na_2_SO_4_ and concentrated *in vacuo* to give crude compound **24b** (200 mg, 0.771 mmol, 61.6% yield) as a gum. LCMS (ESI) *m*/*z*: 259.9 [M+H]^+^.

#### 1-[(1*R*,3*R*)-3-[(5-Isopropylpyrazolo[1,5-*a*]pyrimidin-7-yl)amino]cyclopentyl]-3-methylurea (**24**)

In a sealed tube, a stirred solution of compound **24b** (170 mg, 0.660 mmol) in 1,4-dioxane (2 mL), water (1 mL), and *S*-methyl *N*-methylcarbamothioate (220 mg, 2.09 mmol) was heated at 65 °C for 12 h. The progress of the reaction was monitored by LCMS. After completion of the reaction, the solvent was evaporated under reduced pressure, and the crude material was purified by prep-HPLC to give compound **24** (70 mg, 0.220 mmol, 33.6% yield) as a white solid. ^1^H NMR (400 MHz, DMSO-*d*_6_) δ ppm 7.98 (1 H, d, *J* = 2.08 Hz), 7.54 (1 H, d, *J* = 7.6 Hz), 6.28 (1 H, d, *J* = 2.18 Hz), 5.98 (1 H, s), 5.94 (1 H, d, *J* = 7.24 Hz) 5.58–5.55 (1 H, m), 4.18–4.11 (1 H, m), 4.07–4.01 (1 H, m), 2.95–2.84 (1 H, m), 2.51 (3 H, d, *J* = 4.6 Hz), 2.19–2.08 (1 H, m), 2.03–1.93 (2 H, m), 1.85–1.73 (1 H, m), 1.75–1.61 (1 H, m), 1.43–1.29 (1 H, m), 1.21 (6 H, d, *J* = 6.8 Hz). – δ ppm 7.97 (1 H, d, *J* = 2.08 Hz), 6.31 (1 H, d, *J* = 2.04 Hz), 6.00 (1 H, s), 4.29–4.18 (2 H, m), 3.01–2.91 (1 H, m), 2.69 (3 H, s), 2.36–2.32 (1 H, m), 2.25–1.95 (3 H, m), 1.82–1.69 (1 H, m), 1.64–1.50 (1 H, m), 1.32 (6 H, d, *J* = 6.9 Hz). LCMS (ESI) *m*/*z*: 317.0 [M+H]^+^.

#### 2-Cyano-1-((1*r*,3*r*)-3-((5-Isopropylpyrazolo[1,5-*a*]pyrimidin-7-yl)amino)cyclobutyl)-3-methylguanidine (**15**)

To a solution of compound **15a** (70 mg, 0.180 mmol; see Supporting Information) in isopropyl alcohol (3.5 mL) in a sealed tube was added methanamine (338 mg, 3.59 mmol) in 33% aq. isopropanol at rt, and then the mixture was heated at 70 °C for 4 h. The progress of the reaction was monitored by TLC and LCMS. After completion of the reaction, the solvent was evaporated *in vacuo*, and the crude product was purified by Combiflash column chromatography (silica, 230–400 mesh), eluting with 5% MeOH in CH_2_Cl_2_ to give compound **15** (31.47 mg, 0.096 mmol, 53.20% yield) as an off-white solid. ^1^H NMR (400 MHz, DMSO-*d*_6_) δ ppm 8.03 (1 H, d, *J* = 1.92 Hz), 7.98 (1 H, d, *J* = 6.08 Hz), 7.17 (1 H, d, *J* = 6.5 Hz), 6.90 (1 H, br s), 6.33 (1 H, d, *J* = 2.0 Hz), 5.84 (1 H, s), 4.28–4.24 (1 H, m), 4.19–4.14 (1 H, m), 2.99–2.87 (1 H, m), 2.70 (3 H, d, *J* = 4.5 Hz), 2.59–2.56 (2 H, m), 2.46–2.39 (2 H, m), 1.23 (6 H, d, *J* = 6.8 Hz). ^1^H NMR (400 MHz, CD_3_OD): δ ppm 7.99 (1 H, s), 6.33 (1 H, s), 5.87 (1 H, s), 4.41–4.29 (2 H, m), 3.02–2.90 (1 H, m), 2.83 (3 H, s), 2.62–2.57 (4 H, m), 1.31 (6 H, d, *J* = 7.0 Hz). LCMS (ESI) *m*/*z*: 327.5 [M+H]^+^.

#### (1*S*,3*S*)-*N*3-(5-Isobutyl-3-methylpyrazolo[1,5-*a*]pyrimidin-7-yl)cyclopentane-1,3-diamine (**31**)

To a stirred solution of compound **31c** (60 mg, 0.150 mmol; see Supporting Information) in 1,4-dioxane (2 mL) was added 4 M HCl-dioxane (1 mL, 4 mmol) at 0 °C and stirred at rt for 2 h. The reaction mixture was evaporated *in vacuo*, and the resulting solid was triturated with pentane (3 × 2 mL) and ether (2 × 2 mL). The solid was dried *in vacuo* and lyophilized to give compound **31** (8.2 mg, 0.028 mmol, 18.2% yield) as an off-white solid. ^1^H NMR (400 MHz, DMSO-*d*_6_) δ ppm 7.84 (1 H, s), 7.37 (1 H, d, *J* = 7.4 Hz), 5.95 (1 H, s), 4.27–4.18 (1 H, m), 3.48–3.45 (2 H, m), 2.13–2.07 (5 H, m), 1.99–1.94 (2 H, m), 1.85 (3 H, s), 1.83–1.72 (1 H, m), 1.70–1.63 (1 H, m), 1.38–1.33 (1 H, m), 0.92 (6 H, d, *J* = 6.6 Hz). LCMS (ESI) *m*/*z*: 288.3 [M+H]^+^.

#### *tert*-Butyl *N*-[(1*S*,3*S*)-3-[(5-chloro-pyrazolo[1,5-*a*]pyrimidin-7-yl)amino]cyclopentyl]carbamate (**45**)

A stirred solution of 5,7-dichloro-pyrazolo[1,5-*a*]pyrimidine **44** (2 g, 10.64 mmol), *tert*-butyl ((1*S*,3*S*)-3-aminocyclopentyl)carbamate (2.34 g, 11.7 mmol), and K_2_CO_3_ (4.4 g, 31.91 mmol) in MeCN (20 mL) was heated to reflux for 16 h. The reaction mixture was filtered and concentrated under reduced pressure, and the crude material was purified by Combiflash column chromatography (silica, 230–400 mesh), eluting with 30% ethyl acetate in hexanes to give compound **45** (1.9 g, 5.36 mmol, 50.4% yield) as a white solid. LCMS (ESI) *m*/*z*: 352.1 [M+H]^+^.

#### *tert*-Butyl *N*-[(1*S*,3*S*)-3-[(5-chloro-pyrazolo[1,5-*a*]pyrimidin-7-yl)amino]cyclopentyl]carbamate (**45**)

A solution of *tert*-butyl ((1*S*,3*S*)-3-aminocyclopentyl)carbamate (3.83 g, 19.12 mmol), 5,7-dichloro-pyrazolo[1,5-*a*]pyrimidine **44** (3.6 g, 19.1 mmol), and NEt_3_ (2.68 mL, 19.1 mmol) in MeCN (40 mL) was stirred at 50 °C for 4 h. TLC (petroleum ether/ethyl acetate = 1:1, *R*_f_ = 0.6) indicated starting material was consumed completely and one new spot formed. The reaction mixture was concentrated *in vacuo*, and the residue was purified by Combiflash column chromatography (silica gel, 230–400 mesh), eluting with 0–50% ethyl acetate/petroleum ether gradient at 50 mL/min to give compound **45** (6.8 g,17.4 mmol, 91.0% yield) as a yellow gum. ^1^H NMR (400 MHz, CDCl_3_) δ ppm 7.96 (1 H, d, *J* = 2.6 Hz), 6.43 (1 H, d, *J* = 2.0 Hz), 5.93 (1 H, s), 4.55 (1 H, br s), 4.24–4.16 (1 H, m), 2.43–2.34 (1 H, m), 2.32–2.22 (1 H, m), 2.19–2.02 (2 H, m), 1.74–1.72 (1 H, m), 1.65–1.52 (3 H, m), 1.46 (9 H, s).

#### *tert*-Butyl *N*-[(1*S*,3*S*)-3-[[5-(3-methoxyphenyl)pyrazolo[1,5-*a*]pyrimidin-7-yl]amino]cyclopentyl]carbamate (**36a**)

A stirred mixture of compound **45** (80 mg, 0.230 mmol), (3-methoxyphenyl)boronic acid (69.1 mg, 0.450 mmol), and K_2_CO_3_ (94.3 mg, 0.680 mmol) in 1,4-dioxane (5 mL) was degassed for 30 min using argon, and then Pd_2_(dba)_3_ (20.8 mg, 0.020 mmol) and Xantphos Gen 3 (38.5 mg, 0.050 mmol) were added. The reaction mixture was heated to 100 °C for 16 h. After completion of the reaction, the reaction mixture was filtered through a pad of Celite and purified by Combiflash column chromatography (silica, 230–400 mesh), eluting with 30% ethyl acetate in hexanes to give compound **36a** (60 mg, 0.139 mmol, 61.3% yield) as a yellow, sticky liquid. LCMS (ESI) *m*/*z*: 424.0 [M+H]^+^.

#### [(1*S*,3*S*)-3-[[5-(3-Methoxyphenyl)pyrazolo[1,5-*a*]pyrimidin-7-yl]amino]cyclopentyl]ammonium chloride (**36**)

To **36a** (60 mg, 0.140 mmol) was added 4 M HCl in dioxane (2 mL, 0.140 mmol) at 0 °C and stirred at rt for 4 h. The reaction mixture was evaporated *in vacuo* and triturated with pentane to give compound **36** (30.1 mg, 0.083 mmol, 58.2% yield) as a light-yellow solid. ^1^H NMR (400 MHz, DMSO-*d*_6_) δ ppm 8.09 (1 H, s), 7.98–7.93 (3 H, m), 7.73–7.69 (2 H, m), 7.44 (1 H, t, *J* = 7.6 Hz), 7.08 (1 H, d, *J* = 8.2 Hz), 6.67 (1 H, s), 6.49 (1 H, s), 4.66–4.59 (1 H, m), 3.89 (3 H, s), 3.83–3.78 (1 H, m), 2.34–2.32 (1 H, m), 2.22 (3 H, t, *J* = 7.1 Hz), 1.89–1.87 (1 H, m), 1.74 (1 H, s). LCMS (ESI) *m*/*z*: 324.0 [M+H]^+^.

#### *tert*-Butyl *N*-[(1*S*,3*S*)-3-[[5-(4-Methoxyphenyl)pyrazolo[1,5-*a*]pyrimidin-7-yl]amino]cyclopentyl]carbamate (**37a**)

A stirred solution of compound **45** (80 mg, 0.230 mmol), (4-methoxyphenyl)boronic acid (69.1 mg, 0.450 mmol), and K_2_CO_3_ (94.3 mg, 0.680 mmol) in 1,4-dioxane (5 mL) was degassed for 30 min using argon, and then Pd_2_(dba)_3_ (20.82 mg, 0.020 mmol) and Xantphos Gen 3 (38.5 mg, 0.050 mmol) were added. The reaction mixture was heated at 100 °C for 16 h. After completion of the reaction, the reaction mixture was filtered through a pad of Celite, and the filtrate was evaporated *in vacuo*. The crude mixture was purified by Combiflash column chromatography (silica, 230–400 mesh), eluting with 30–40% ethyl acetate in hexanes to give compound **37a** (80 mg, 0.187 mmol, 82.1% yield) as a yellow sticky liquid. LCMS (ESI) *m*/*z*: 424.0 [M+H]^+^.

#### [(1*S*,3*S*)-3-[[5-(4-Methoxyphenyl)pyrazolo[1,5-*a*]pyrimidin-7-yl]amino]cyclopentyl]ammonium Chloride (**37**)

To compound **37a** (80 mg, 0.190 mmol) was added 4 M HCl in dioxane (2 mL, 0.190 mmol) at 0 °C and then stirred at rt for 4 h. The reaction mixture was evaporated *in vacuo* and triturated with pentane to give compound **37** (30.9 mg, 0.085 mmol, 45.3% yield) as an off-white solid. ^1^H NMR (400 MHz, DMSO-*d*_6_) δ ppm 8.15–8.11 (2 H, m), 8.07 (1 H, s), 8.03–7.95 (2 H, m), 7.80–7.74 (1 H, m), 7.08 (2 H, d, *J* = 8.0 Hz), 6.66 (1 H, s), 6.45 (1 H, s), 4.64 (1 H, d, *J* = 6.3 Hz), 3.87 (3 H, s), 3.83–3.78 (1 H, m), 2.35–2.32 (1 H, m), 2.26–2.18 (3 H, m), 1.93–1.83 (1 H, m), 1.77–1.72 (1 H, m). LCMS (ESI) *m*/*z*: 324.2 [M+H]^+^.

#### *tert*-Butyl *N*-[(1*S*,3*S*)-3-[[5-(2-methoxyphenyl)pyrazolo[1,5-*a*]pyrimidin-7-yl]amino]cyclopentyl]carbamate (**35a**)

A stirred solution of compound **45** (80 mg, 0.230 mmol), (2-methoxyphenyl)boronic acid (69.1 mg, 0.450 mmol), and K_2_CO_3_ (94.3 mg, 0.680 mmol) in 1,4-dioxane (5 mL) was degassed for 30 min, and Pd_2_(dba)_3_ (20.8 mg, 0.020 mmol) and Xantphos Gen 3 (38.5 mg, 0.050 mmol) were added. The reaction mixture was heated at 100 °C for 16 h. After completion of the reaction, the reaction mixture was filtered through a pad of Celite and purified by Combiflash column chromatography (silica, 230–400 mesh), eluting with 30–40% ethyl acetate in hexanes to give compound **35a** (50 mg, 0.118 mmol, 51.9% yield) as a yellow sticky liquid. LCMS (ESI) *m*/*z*: 424.2 [M+H]^+^.

#### [(1*S*,3*S*)-3-[[5-(2-Methoxyphenyl)pyrazolo[1,5-*a*]pyrimidin-7-yl]amino]cyclopentyl]ammonium Chloride (**35**)

To compound **35a** (50 mg, 0.120 mmol) was added 4 M HCl in dioxane (2 mL, 0.120 mmol) at 0 °C and then stirred at rt for 4 h. The reaction mixture was evaporated *in vacuo* and triturated with pentane to give compound **35** (23.7 mg, 0.065 mmol, 55.4% yield) as a light-brown solid. ^1^H NMR (400 MHz, DMSO-*d*_6_) δ ppm 8.6 (1 H, m), 8.18–7.75 (4 H, m), 7.74 (1 H, d, *J* = 7.7 Hz), 7.53 (1 H, t, *J* = 7.9 Hz), 7.23 (1 H, d, *J* = 8.3 Hz), 7.13 (1 H, t, *J* = 7.5 Hz), 6.72 (1 H, s), 6.53 (1 H, s), 4.63 (1 H, s), 3.91 (3 H, s), 3.79 (1 H, s), 2.33–2.21 (4 H, m), 1.98–1.84 (1 H, m), 1.81–1.71 (1 H, m). LCMS (ESI) *m*/*z*: 324.4 [M+H]^+^.

#### Ethyl 4-Ethyl-3-oxo-hexanoate (**47**)

2-Ethylbutanoic acid (10 g, 86.1 mmol) was dissolved in THF (200 mL) and cooled to 0 °C. After stirring at 0 °C for 20 min, CDI (21.6 g, 133 mmol) was added portion-wise. The temperature was allowed to rise to rt, and the mixture was stirred at rt for 16 h. In a second reaction flask, MgCl_2_ (8.19 g, 86.1 mmol) and potassium 3-ethoxy-3-oxo-propanoate (22.7 g, 133 mmol) were mixed in THF (200 mL) and stirred under argon overnight at 50 °C. The resultant white suspension from the second reaction flask was cooled to rt, the first flask contents were added dropwise over 10 min, and the reaction mixture was stirred for 16 h at rt. A difficult to stir chewing gum-like solid appeared upon initial addition, but after several hours the reaction mixture became more homogeneous and easier to stir. The reaction mixture was concentrated to approximately a third of its final combined volume, taken up in an equal volume of sat. potassium bisulfate solution, and extracted twice with ethyl acetate. The combined organic layers were washed with sat. sodium bicarbonate solution, dried over anhyd. Na_2_SO_4_, and evaporated *in vacuo*. The crude material was purified by Combiflash column chromatography (silica, 230–400 mesh), eluting with ethyl acetate–hexane to give compound **47** (7.2 g, 38.7 mmol, 44.9% yield) as a transparent liquid.

#### 5-(1-Ethylpropyl)-4*H*-pyrazolo[1,5-*a*]pyrimidin-7-one (**48**)

A mixture of compound **47** (7 g, 37.6 mmol) and 1*H*-pyrazol-5-amine (3.12 g, 37.6 mmol) in acetic acid (35 mL) was heated at 110 °C for 3 h. The solvent was evaporated *in vacuo*, and the residue was treated with ethyl acetate and filtered to give compound **48** (2.1 g, 9.96 mmol, 26.5% yield) as an off-white solid. LCMS (ESI) *m*/*z*: 206.4 [M+H]^+^.

#### 7-Chloro-5-(1-ethylpropyl)pyrazolo[1,5-*a*]pyrimidine (**49**)

A stirred solution of compound **48** (300 mg, 1.46 mmol) in POCl_3_ (2.1 mL, 22.5 mmol) was heated to 100 °C for 4 h. The reaction mixture was brought to rt, and the excess reagent was removed *in vacuo*. The residue was treated with ice–water, and the chlorinated product was extracted from the aqueous mixture by DCM. The organic layer was separated, dried over anhyd. Na_2_SO_4_, and purified by Combiflash column chromatography (silica, 230–400 mesh), eluting with 10–20% ethyl acetate in hexanes to give **49** (180 mg, 0.797 mmol, 54.5% yield) as a light-yellow liquid. LCMS (ESI) *m*/*z*: 224.1 [M+H]^+^.

#### (1*S*,3*S*)-*N*3-[3-Chloro-5-(1-ethylpropyl)pyrazolo[1,5-*a*]pyrimidin-7-yl]cyclopentane-1,3-diamine Hydrochloride (**29**)

To a stirred solution of *tert*-butyl compound **29b** (80 mg, 0.190 mmol; see Supporting Information) in 1,4-dioxane (6.40 mL) was added 4 M HCl–dioxane (0.5 mL, 2 mmol) at 0 °C and stirred at rt for 2 h. The reaction mixture was evaporated *in vacuo*, and the resulting solid was triturated with pentane (3 × 2 mL) and ether (2 × 2 mL). The solid was then dried *in vacuo* and lyophilized to give compound **29** (65 mg, 0.180 mmol, 95.0% yield) as an off-white solid. ^1^H NMR (400 MHz, DMSO-*d*_6_) δ ppm 9.03–8.49 (1 H, m), 8.36–8.05 (4 H, m), 6.44 (1 H, s), 4.64 (1 H, s), 3.75–3.64 (2 H, m), 3.53–3.43 (1 H, m), 2.65–2.59 (1 H, m), 2.30–1.99 (4 H, m), 1.88–1.59 (6 H, m), 0.80 (6 H, t, *J* = 7.3 Hz). LCMS (ESI) *m*/*z*: 322.4 [M+H]^+^.

#### *tert*-Butyl *N*-[(1*S*,3*S*)-3-[[5-(*o*-tolyl)pyrazolo[1,5-*a*]pyrimidin-7-yl]amino]cyclopentyl]carbamate (**32a**)

To a solution of compound **45** (50 mg, 0.14 mmol), *o*-tolylboronic acid (38 mg, 0.28 mmol), K_2_CO_3_ (59 mg, 0.43 mmol), and Pd(dppf)Cl_2_ (2 mg, 0.01 mmol) were added 1,4-dioxane (2.0 mL) and water (0.2 mL). The mixture was degassed and purged with N_2_ for 3 times and then stirred at 90 °C for 4 h under N_2_. The reaction mixture was concentrated *in vacuo*, and the residue was purified by prep-TLC (SiO_2_, petroleum ether/ethyl acetate 1:1, *R*_f_ = 0.6) to give compound **32a** (55 mg, 0.13 mmol, 94% yield) as a white solid. LCMS (ESI) *m/z:* 408.2 [M+H]^+^.

#### (1*S*,3*S*)-*N*3-[5-(*o*-Tolyl)pyrazolo[1,5-*a*]pyrimidin-7-yl]cyclopentane-1,3-diamine (**32**)

To a solution of compound **32a** (100 mg, 0.25 mmol) in DCM (3.0 mL) was added TFA (1.0 mL, 11.6 mmol), and the mixture was stirred at 25 °C for 12 h. The resulting solution was cooled to 0 °C and basified by addition of aq. sat. NaHCO_3_. The reaction mixture was concentrated *in vacuo*, and the residue was purified by prep-HPLC (neutral condition) to give compound **32** (17.7 mg, 0.056 mmol, 23% yield) as a yellow solid. LCMS (ESI) *m/z*: 308.2 [M+H]^+^. ^1^H NMR (400 MHz, CD_3_OD) δ ppm = 8.06 (1 H, d, *J* = 2.4 Hz), 7.42–7.26 (4 H, m), 6.42 (1 H, d, *J* = 2.0 Hz), 6.19 (1 H, s), 4.35 (1 H, quin, *J* = 6.7 Hz), 3.61 (1 H, quin, *J* = 6.6 Hz), 2.76–2.70 (1 H, m), 2.43–2.42 (1 H, m), 2.45–2.30 (1 H, m), 2.24–2.14 (1 H, m), 2.13–2.03 (1 H, m), 2.03–1.93 (1 H, m), 1.88–1.69 (1 H, m). LCMS (ESI) *m*/*z*: 308.2 [M+H]^+^.

#### *tert*-Butyl *N*-[1*S*,3*S*)-3-[[5-(5-chloro-2-methylphenyl)pyrazolo[1,5-*a*]pyrimidin-7-yl]amino]cyclopentyl]carbamate (**34a**)

A mixture of compound **45** (50 mg, 0.14 mmol), (5-chloro-2-methyl-phenyl)boronic acid (72 mg, 0.43 mmol), K_2_CO_3_ (59 mg, 0.43 mmol), and Pd(dppf)Cl_2_ (10 mg, 0.01 mmol) in 1,4-dioxane (3 mL) and water (0.30 mL) was degassed and purged with N_2_ 3 times, and then the mixture was stirred at 90 °C for 12 h under N_2_ atmosphere. The reaction mixture was concentrated under reduced pressure to give a residue which was purified by prep-TLC (SiO_2_, petroleum ether/ethyl acetate 1:1, *R*_f_ = 0.7) to give compound **34a** (45 mg, 0.038 mmol, 26% yield) as a yellow solid. LCMS (ESI) *m*/*z*: 442[M+H]^+^.

#### (1*S*,3*S*)-*N*3-[5-(5-Chloro-2-methylphenyl)pyrazolo[1,5-*a*]pyrimidin-7-yl]cyclopentane-1,3-diamine (**34**)

To a solution of compound **34a** (40 mg, 0.09 mmol) in DCM (3 mL) was added TFA (1.00 mL, 11.6 mmol). The mixture was stirred at 20 °C for 0.5 h. The reaction mixture was concentrated *in vacuo* to give a residue. The residue was dissolved in MeOH (2 mL) and basified to pH 8 by NH_3_·H_2_O (25% in H_2_O). Then the residue was purified by prep-HPLC (neutral conditions) to give compound **34** (9.85 mg, 0.0285 mmol, 31% yield) as a yellow solid. ^1^H NMR (400 MHz, CD_3_OD) δ ppm 8.07 (1 H, d, *J* = 2.2 Hz), 7.42 (1 H, d, *J* = 2.0 Hz), 7.38–7.29 (2 H, m), 6.44 (1 H, d, *J* = 2.2 Hz), 6.21 (1 H, s), 4.36 (1 H, q, *J* = 6.9 Hz), 3.70–3.54 (1 H, m), 2.44–2.30 (4 H, m), 2.27–2.15 (1 H, m), 2.14–1.94 (2 H, m), 1.87–1.74 (1 H, m), 1.59–1.46 (1 H, m). LCMS (ESI) *m*/*z*: 342.1 [M+H]^+^.

#### (1*S*,3*S*)-*N*3-(5-Chloro-pyrazolo[1,5-*a*]pyrimidin-7-yl)cyclopentane-1,3-diamine (**46**)

A solution of compound **45** (900 mg, 2.56 mmol) in HCl/EtOAC (15 mL, 60 mmol) was stirred at 25 °C for 12 h. The reaction mixture was concentrated and then reconstituted in DCM/MeOH (10:1, 15 mL). The mixture was basified by sat. NaHCO_3_ (∼50 mL) until pH ∼ 8 in an ice bath. The resulting mixture was extracted with DCM/MeOH (10:1,15 mL × 3). The combined organic layers were dried over Na_2_SO_4_ and concentrated to give compound **46** as a pale-yellow oil. LCMS (ESI) *m*/*z*: 252.0 [M+H]^+^.

#### (1*S*,3*S*)-*N*3-[5-(5-Fluoro-2-methylphenyl)pyrazolo[1,5-*a*]pyrimidin-7-yl]cyclopentane-1,3-diamine (**33**)

To a solution of compound **46** (50 mg, 0.20 mmol) in DMF (1 mL) were added (5-fluoro-2-methyl-phenyl)boronic acid (45.9 mg, 0.30 mmol), potassium phosphate (0.3 mL, 0.60 mmol), and tetraphenylphosphine palladium (23.0 mg, 0.02 mmol). The mixture was bubbled with nitrogen for 1 min and then stirred at 130 °C for 10 min in the microwave. LCMS showed that the starting material was completely consumed, and the desired mass was detected. The reaction mixture was filtered, and the filtrate was purified by prep-HPLC (Waters Xbridge Prep OBD C18 150 mm × 50 mm, 10 μm column; 15–45% acetonitrile in a 10 mM ammonium bicarbonate solution in water, 8 min gradient). Compound **33** (10.8 mg,0.03 mmol, 16.2% yield) was obtained as a white solid. LCMS (ESI) *m*/*z* 326.2 [M+H]^+^. ^1^H NMR (400 MHz, CD_3_OD) δ ppm 8.07 (1 H, d, *J* = 2.4 Hz), 7.33–7.31 (1 H, m), 7.18–7.15 (1 H, m), 7.09–7.08 (1 H, m), 6.44 (1 H, d, *J* = 2.4 Hz), 6.17 (1 H, s), 4.44–4.37 (1 H, m), 3.68–3.61 (1 H, m), 2.39–2.37 (1 H, m), 2.34 (1 H, s), 2.27–2.25 (1 H, m), 2.15–2.09 (1 H, m), 1.86–1.83 (1 H, m), 1.62–1.60 (1 H, m), 1.59–1.57 (1 H, m).

#### 1-((1*r*,3*r*)-3-((5-Isopropylpyrazolo[1,5-*a*]pyrimidin-7-yl)amino)cyclobutyl)-3-methylurea (**14**).

*S*-Methyl *N*-methylcarbamothioate (300 mg, 2.85 mmol) was added to a stirred solution of compound 8 (200 mg, 0.82 mmol) in 1,4-dioxane (2.5 mL) and water (1.5 mL) in a sealed tube at rt. The reaction mixture was heated at 65 °C for 12 h. The progress of the reaction was monitored by LCMS. After completion of reaction, the reaction mixture was evaporated in vacuo and purified by Combiflash column chromatography (silica, 230–400 mesh), eluting with 5% MeOH in CH_2_Cl_2_ to give (100 mg, 0.323 mmol, 39.6% yield) as a white solid. ^1^H NMR (400 MHz, DMSO-*d*_6_) δ ppm 8.02 (1 H, d, *J* = 2.2 Hz), 7.96 (1 H, d, *J* = 6.1 Hz), 6.35–6.27 (2 H, m), 5.81 (1 H, s), 5.68 (1 H, d, *J* = 4.8 Hz), 4.22–4.10 (2 H, m), 2.98–2.86 (1 H, m), 2.54 (3 H, d, *J* = 4.6 Hz), 2.48–2.42 (2 H, m), 2.33–2.22 (2 H, m), 1.22 (6 H, d, *J* = 6.9 Hz). LCMS (ESI) *m*/*z*: 303.4 [M + H]^+^.

#### *tert*-Butyl *N*-[(1*S*,3*S*)-3-[[5-(1-Ethylpropyl)pyrazolo[1,5-*a*]pyrimidin-7-yl]amino]cyclopentyl]carbamate (**28a**).

A stirred solution of compound **49** (2.4 g, 10.73 mmol), *tert*-butyl ((1*S*,3*S*)-3-aminocyclopentyl)carbamate (2.36 g, 11.8 mmol) and K_2_CO_3_ (4.44 g, 32.19 mmol) in MeCN (25 mL) was heated to reflux for 16 h. The reaction mixture was cooled and filtered. The filtrate was concentrated in vacuo, and the residue was purified by Combiflash column chromatography, eluting with 30% ethyl acetate in hexanes to give compound **28a** (3.6 g, 9.05 mmol, 84.3% yield) as an off-white solid. LCMS (ESI) *m*/*z*: 388.3 [M + H]^+^.

#### [(1*S*,3*S*)-3-[[5-(1-Ethylpropyl)pyrazolo[1,5-*a*]pyrimidin-4-ium-7-yl]amino]cyclopentyl]ammonium Dichloride (**28**).

To **28a** (3.4 g, 8.77 mmol) in 1,4-dioxane (6.8 mL) was added 4 M HCl in dioxane (10.6 mL, 43.9 mmol) at 0 °C and stirred at rt for 4 h. The reaction mixture was evaporated in vacuo, triturated with pentane, and lyophilized from H_2_O to give compound 28 (3 g, 8.27 mmol, 94.3% yield) as an off-white sticky solid. ^1^H NMR (400 MHz, DMSO-*d*_6_) δ ppm 9.13 (1 H, s), 8.40 (3 H, s), 8.20 (1 H, d, *J* = 2.1 Hz), 6.62 (1 H, s), 6.56 (1 H, d, *J* = 2.2 Hz), 4.90 (1 H, s), 3.77 (1 H, s), 2.86–2.74 (1 H, m), 2.38–2.14 (4 H, m), 1.97–1.73 (6 H, m), 0.88 (6 H, t, *J* = 7.4 Hz). LCMS (ESI) *m*/*z*: 288.2 [M + H]^+^.

#### *tert*-Butyl ((1*R*,3*R*)-3-((5-(Pentan-3-yl)pyrazolo[1,5-*a*]pyrimidin-7-yl)amino)cyclopentyl)carbamate (**38a**).

To a stirred solution of compound **49** (4.8 g, 21.5 mmol) in MeCN (50 mL) was added *tert*-butyl ((1*R*,3*R*)-3-aminocyclopentyl)carbamate (4.3 g, 21.5 mmol) and K_2_CO_3_ (8.89 g, 64.4 mmol) at 20 °C before the mixture was stirred at 80 °C for 12 h. The progress of the reaction was monitored by LCMS. The reaction mixture was cooled to 20 °C, poured into H_2_O (100 mL), and extracted with ethyl acetate (3 × 200 mL). The combined organic layers were washed with brine (300 mL), dried over anhyd. Na_2_SO_4_, filtered and concentrated under reduced pressure to give compound **38a** (7.6 g, 19.6 mmol, 91.6% yield) as a yellow solid, which was used directly in the next step without further purification. LCMS (ESI) *m*/*z*: 388.3 [M + H]^+^.

#### (1*R*,3*R*)-*N*1-(5-(Pentan-3-yl)pyrazolo[1,5-*a*]pyrimidin-7-yl)cyclopentane-1,3-diamine (**38**).

To a solution of compound **38a** (8.0 g, 20.6 mmol) in ethyl acetate (40 mL) was added HCl/ethyl acetate (40 mL, 614 mmol) and the mixture was stirred at 15 °C for 24 h. The progress of the reaction was monitored by LCMS. After completion of reaction, the reaction mixture was evaporated in vacuo dissolved in H_2_O (200 mL) and extracted with ethyl acetate (3 × 100 mL). The aqueous phase was adjusted to pH = 8 with ammonium hydroxide and extracted with CH_2_Cl_2_/MeOH (10:1, 3 × 300 mL). The combined organic layers were washed with brine (2 × 300 mL), dried over anhyd. Na_2_SO_4_, filtered, and concentrated under reduced pressure. The crude product was purified by flash column chromatography (ISCO 40 g silica, 0–10% MeOH in CH_2_Cl_2_, gradient over 20 min) to give compound 38 (5.1 g, 17.745 mmol, 86.0% yield) as a yellow solid. ^1^H NMR (400 MHz, CD_3_OD) δ ppm 7.98 (1 H, d, *J* = 2.0 Hz), 6.32 (1 H, d, *J* = 2.4 Hz), 6.02 (1 H, s), 4.35–4.28 (1 H, m), 3.59–3.53 (1 H, m), 2.51–2.49 (1 H, m), 2.49–2.47 (1 H, m), 2.16–2.01 (1 H, m), 2.00–1.97 (2 H, m), 1.77–1.70 (5 H, m), 1.52–1.47 (1 H, m), 0.84 (6 H, t, *J* = 7.6 Hz). LCMS (ESI) *m*/*z*: 288.2 [M + H]^+^.

#### *tert*-Butyl ((1*S*,3*R*)-3-((5-(Pentan-3-yl)pyrazolo[1,5-*a*]pyrimidin-7-yl)amino)cyclopentyl)carbamate (**39a**).

To a stirred solution of compound **49** (10.1 g, 44.9 mmol) in MeCN (150 mL) was added *tert*-butyl ((1*R*,3*S*)-3-aminocyclopentyl)carbamate (9.0 g, 44.9 mmol) and K_2_CO_3_ (18.6 g, 135 mmol) at 20 °C before the mixture was stirred at 80 °C for 16 h. The progress of the reaction was monitored by LCMS. The reaction mixture was cooled to 20 °C, poured into H_2_O (100 mL), and extracted with ethyl acetate (3 × 200 mL). The combined organic layers were washed with brine (300 mL), dried over anhyd. Na_2_SO_4_, filtered and concentrated under reduced pressure to give compound **39a** (20 g, 51.6 mmol, >100.0% yield) as a pale, yellow gum, which was used directly in the next step without further purification. LCMS (ESI) *m*/*z*: 388.4 [M + H]^+^.

#### (1*R*,3*S*)-*N*1-(5-(Pentan-3-yl)pyrazolo[1,5-*a*]pyrimidin-7-yl)cyclopentane-1,3-diamine (**39**).

To a solution of compound **39a** (20 g, 51.6 mmol) in ethyl acetate (100 mL) was added HCl/ethyl acetate (100 mL, 400 mmol) and the mixture was stirred at 20 °C for 10 h. The progress of the reaction was monitored by LCMS. After completion of reaction, the reaction mixture was poured into sat. NaHCO_3_ (200 mL) and extracted with ethyl acetate (200 mL) and CH_2_Cl_2_ (2 × 300 mL). The combined organic layers were washed with brine (50 mL), dried over anhyd. Na_2_SO_4_, filtered, and concentrated under reduced pressure. The crude product was purified by flash column chromatography (300 g Agela C18, 35–65% MeOH in NH_3_H_2_O, gradient over 55 min) to give compound **39** (8.5 g, 29.37 mmol, 57.3% yield) as a yellow gum. ^1^H NMR (400 MHz, CD_3_OD) δ ppm 7.96 (1 H, d, *J* = 1.8 Hz), 6.38–6.28 (1 H, m), 5.98 (1 H, s), 4.22–4.16 (1 H, m), 3.49–3.41 (1 H, m), 2.62–2.41 (2 H, m), 2.27–2.11 (1 H, m), 2.01 (1 H, qd, *J* = 6.8, 13.2 Hz), 1.94–1.79 (1 H, m), 1.77–1.58 (5 H, m), 1.57–1.41 (1 H, m), 0.82 (6 H, t, *J* = 7.2 Hz). LCMS (ESI) *m*/*z*: 288.2 [M + H]^+^.

#### *tert*-Butyl ((1*R*,3*S*)-3-((5-(Pentan-3-yl)pyrazolo[1,5-*a*]pyrimidin-7-yl)amino)cyclopentyl)carbamate (**40a**).

To a stirred solution of compound **49** (10.05 g, 44.94 mmol) in MeCN (150 mL) was added *tert*-butyl ((1*S*,3*R*)-3-aminocyclopentyl)carbamate (9.00 g, 44.94 mmol) and K_2_CO_3_ (18.60 g, 134.81 mmol) at 20 °C before the mixture was stirred at 80 °C for 15 h. The progress of the reaction was monitored by LCMS. The reaction mixture was cooled to 20 °C, poured into H_2_O (100 mL), and extracted with ethyl acetate (3 × 200 mL). The combined organic layers were washed with brine (300 mL), dried over anhyd. Na*2*SO*4*, filtered and concentrated under reduced pressure to give compound **40a** (18.0 g, 46.5 mmol, 103.4% yield) as a yellow oil, which was used directly in the next step without further purification. LCMS (ESI) *m*/*z*: 388.2 [M + H]^+^.

#### (1*S*,3*R*)-*N*1-(5-(Pentan-3-yl)pyrazolo[1,5-*a*]pyrimidin-7-yl)cyclopentane-1,3-diamine (**40**).

To a solution of compound **40a** (19.4 g, 49.9 mmol) in ethyl acetate (125 mL) was added HCl/ethyl acetate (125 mL, 499 mmol) and the mixture was stirred at 20 °C for 15 h. The progress of the reaction was monitored by LCMS. After completion of reaction, the reaction mixture was evaporated in vacuo dissolved in H_2_O (200 mL) and extracted with ethyl acetate (3 × 100 mL). The aqueous phase was adjusted to pH = 8 with ammonium hydroxide and extracted with CH_2_Cl_2_/MeOH (10:1, 3 × 300 mL). The combined organic layers were washed with brine (2 × 300 mL), dried over anhyd. Na_2_SO_4_, filtered, and concentrated under reduced pressure. The crude product was purified by flash column chromatography (ISCO 120 g silica, 0–10% MeOH in CH_2_Cl_2_, gradient over 20 min) to give compound 40 (11.0 g, 38.3 mmol, 76.7% yield) as a pale, yellow gum. ^1^H NMR (400 MHz, CD_3_OD) δ ppm 7.98 (1 H, d, *J* = 2.0 Hz), 6.33 (1 H, d, *J* = 2.4 Hz), 6.00 (1 H, s), 4.20–4.16 (1 H, m), 3.46–3.42 (1 H, m), 2.56–2.47 (2 H, m), 2.25–2.16 (1 H, m), 2.15–1.95 (1 H, m), 1.94–1.83 (1 H, m), 1.77–1.70 (4 H, m), 1.68–1.55 (1 H, m), 1.54–1.52 (1 H, m), 0.84 (6 H, t, *J* = 7.6 Hz). LCMS (ESI) *m*/*z*: 288.2 [M + H]^+^.

### CDK Kinases Assay

*In vitro* kinase profiling of the CDK kinase panel was performed at Reaction Biology Corporation (www.reactionbiology.com, Malvern, PA) using the “HotSpot” assay platform. Assay performed as described.^37^ In summary, each CDK and cyclin pairing along with required cofactors was prepared in base reaction buffer [20 mM HEPES (pH 7.5), 10 mM MgCl_2_, 1 mM EGTA, 0.01% Brij35, 0.02 mg/mL BSA, 0.1 mM Na_3_VO_4_, 2 mM DTT, 1% DMSO]. Compounds in DMSO stock solutions are then acoustically delivered to the CDK/cyclin buffer suspension and incubated for 20 min before a mixture of ATP (Sigma, St. Louis MO) and^32^P-ATP (specific activity 0.01 μCi/μL final; PerkinElmer, Waltham MA) to a final concentration of 10 μM is added to initiate the reaction. The reactions are incubated for 2 h at rt whereupon reactions are spotted onto P81 ion exchange paper (Whatman Inc., Piscataway, NJ). The filters are washed extensively with 0.75% phosphoric acid, and the remaining radioactive phosphorylated substrate remaining on the paper was measured. Kinase activity data were expressed as the percent remaining kinase activity in test samples compared to that in vehicle (DMSO) reactions. IC_50_ values and curve fits were obtained using Prism4 Software (GraphPad). Kinome tree representations were prepared using Kinome Mapper (https://www.reactionbiology.com/resources/tools/kinase-mapper).

### Ligand Docking Protocol

The crystal structure of CDK9 (PDB:3MY1) was used for this study. Structure preparation and molecular docking were performed by using MOE software. Protein structure was prepared using the MOE “Structure Preparation” protocol with all default parameters. Ligand, **28**, was prepared using MOE and properly protonated. “Induced Fit” molecular docking protocol was used. Top five docking poses were kept and further analyzed.

### Quantum Mechanics Calculation

The torsional profile between pyrazolo[1,5-*a*]pyrimidine ring and diamino-cyclopentane ring of docked pose for each diamino-cyclopentane diastereomers was analyzed using GAUSSIAN 16 software at the B3LYP/6-31G* level with PCM as solvent model.^38^ The energy difference between the docked conformer and the conformer with the lowest energy was defined as the molecular strain energy in this study.

### X-ray Crystallography

The CDK9 and CyclinT1 constructs were designed as reported.^39^ GST-TEV-CDK9 (M1-T330, S7D, V8N, K44R, Y138F, K280A, D307E, N311E) and His-Flag-TEV-CyclinT1(M1-R259, R26A, Q77R, E96G, K106R, F241L) were cloned into the pFastBac1 vector. The baculovirus CDK9 and cyclinT1 was added in a 1:1 ratio to infect SF21 cells to express the CDK9/cyclin T1 complex. The cell pellet containing protein complex was loaded onto the Ni-NTA column and then the GST column followed by TEV cleavage to remove the GST-tag on CDK9 and the His tag on cyclin T1. The protein complex was further separated through reverse Nickel and GST column and purified by SEC column (Superdex 200 Increase 10/300 GL) in a final buffer 20 mM Tris-HCl (pH 8.0), 500 mM NaCl, 5 mM DTT. The protein complex was concentrated to 5.66 mg/mL and incubated with **28** on ice for 1 h before setting up crystallization trays. The co-crystals of CDK9-cyclin T1-**28** were obtained from 0.2 M potassium citrate and 20% PEG 3350 before being harvested and flash-cooled in liquid nitrogen for data collection. A 3.75 Å dataset was collected on beamline BL45XU at Spring-8 synchrotron source, indexed and integrated with XDS,^40^ and scaled by aimless. The CDK9-CyclinT1 complex structure was solved by molecular replacement with Phaser module in the CCP4 package suite,^41^ using the coordinates of PDB 3BLH as the search model. Compound **28** was placed into the electron density based on the difference electron density maps. After multiple cycles of iterative refinement with Refmac module in the CCP4 package suite and manual adjustment in the Coot program,^42^ the complex structure was finally refined to *R*_work_/*R*_free_ as 19.85%/24.99%, respectively. The refinement statistics are listed in Table S1.

### OncoPanel Multiplexed Cytotoxicity Assay

On-cell compound profiling was performed at Eurofins Discovery Services (www.eurofinsdiscovery.com, St. Charles, MO) using the OncoPanel Multiplexed Cytotoxicity assay platform. Cells were grown in RPMI 1640, 10% FBS, 2 mM l-alanyl-l-glutamine, 1 mM Na pyruvate, or a special medium. Cells were seeded into 384-well plates and incubated in a humidified atmosphere of 5% CO2 at 37 °C. Compounds were added the day following cell seeding. At the same time, a time zero untreated cell plate was generated. After a 3-day incubation period, cells were fixed and stained with fluorescently labeled antibodies and nuclear dye to allow imaging of nuclei, apoptotic cells, and mitotic cells. Compound **28** was serially diluted in 2-fold steps from the highest test concentration specified in the above table and assayed over 10 concentrations with a maximum assay concentration of 0.1% DMSO. Automated fluorescence microscopy was carried out using a Molecular Devices ImageXpress Micro XL high-content imager, and images were collected with a 4× objective. 16-bit TIFF images were acquired and analyzed with MetaXpress 5.1.0.41 software. Cell proliferation was measured by the fluorescence intensity of the incorporated nuclear dye. The output is referred to as the relative cell count, where the measured nuclear intensity is transformed to the percent of control.

### Xenograft Tumor Models

Athymic Nude-*Foxn1*^*nu*^ immunocompromised female mice between 5 and 8 weeks of age were implanted subcutaneously into the left flank with TNBC tumor model fragments. After tumors grew to 150–300 mm^3^, mice (*n* = 10/group) were administered vehicle p.o., **28** at 60 mg/kg p.o. QD×1 followed by (QD×3 on, QD×4 off) for 4 cycles and standard of care [Carboplatin 50 mg/kg i.p. Q14×2 + Paclitaxel 10 mg/kg i.v. Q14×2 or Cisplatin 5 mg/kg i.p. Q7D×3 + Gemcitabine 100 mg/kg i.p. Q7D×3 or Carboplatin 40 mg/kg i.p. Q7D×3 + Gemcitabine 100 mg/kg i.p. Q7D×3 for 3 cycles]. All experimental procedures were performed according to the guidelines of the Institutional Animal Care and Use Committee (IACUC) of Champions Oncology. After inoculation, the animals were checked daily for morbidity and mortality. At the time of routine monitoring, the animals were checked for any effects of tumor growth and treatments on normal behavior such as mobility, food and water consumption, tumor volume, body weight gain/loss (body weights and tumor volumes were measured twice weekly), eye/hair matting, and any other abnormal effect. Death and observed clinical signs were recorded on the basis of the numbers of animals within each subset. Tumor volume (TV) was calculated using the formula (0.52[length × width^2^]). Inhibition of tumor growth (TGI) was determined by calculating the percent TGI with the following formula: (100% × [1 – (final TV × initial TV of a treated group)/(final TV – initial TV of the control group)]). Treatment started on Day 0. Statistical analyses comparing all groups were performed by one-way ANOVA followed by Tukey’s multiple comparisons test (GraphPad Prism 8.4.3). *P*-values <0.05 are statistically significant.

### Analysis of RNAP II (Ser2/5) and MYC in Tumor Samples

Tumors were harvested at 2 h and at 8 h post-dose. Tumors were bisected: half of the tumor was flash frozen, placed on dry ice, and stored at −80 °C, and the other half was fixed in 10% neural-buffered formalin for 18–24 h and then transferred to 70% ethanol at rt until paraffin was embedded. Tumors <250 mm^3^ were processed as a single snap-frozen sample. Phosphorylation of the RNAP II large subunit POLR2A (Ser2/5) and MYC levels were measured via Homogeneous Time Resolved Fluorescence (HTRF) available from Cisbio (www.cisbio.com, Bedford, MA). Frozen tumor tissue was homogenized by using a mortar and pestle and lysed according to the manufacturer’s protocol. Protein concentrations were measured using a Pierce BCA Protein assay kit (www.thermofisher.com, Waltham, MA). Optimized protein concentration (1–2 mg/mL) was added to the appropriate well of a white-walled 384-well plate. HTRF antibodies [Anti-Human c-Myc-Eu Cryptate Antibody/Anti-Human c-Myc-d2 Antibody and Anti-Human pRNA (S2/S5)-Eu Cryptate Antibody/Anti-Human pRNA (S2/S5)-d2 Antibody] were then combined and diluted into detection buffer, and 4 μL was added to each well. The plate was then incubated at rt out of light for 3 h, before being read on the Envision platform (www.perkinelmer.com, Waltham, MA).

## Supporting Information Available

X-ray crystal data collection and refinement statistics of compound **28** bound to ATP-competitive binding site of CDK9 in complex with cyclin T1; experimental procedures for the synthesis of select reagents and intermediates; kinome selectivity of compound **28** as determined by Kinase Hotspot; stereochemical effect on CDK panel selectivity among analogues **28**, **38**, **39**, and **40**; plotted viability effects of **28** on TNBC cell lines; OncoPanel Multiplexed Cytotoxicity Assay control compound data; and two additional TNBC PDX models treated with **28** (PDF)
Molecular formula strings containing chemical structures and biochemical characterization of relevant compounds (CSV)

## Accession Codes

The coordinates of compound **28** with CDK9 in complex with cyclin T1 have been deposited in RCSB Protein Data Bank (PDB) under accession code8K5R.

## Abbreviations Used

ACN: acetonitrile
ADME: absorption, distribution, metabolism, and excretion
anhyd.: anhydrous
aq.: aqueous
ATP: adenosine 5′-triphosphate
BSA: bovine serum albumin
CDI: 1,1′-carbonyldiimidazole
CDK: cyclin-dependent kinase
DCM: dichloromethane
DIPEA: *N,N*-diisopropylethylamine
DMF: *N,N*-dimethylformamide
DMSO: dimethyl sulfoxide
DTT: dithiothreitol
EGTA: ethylene glycol-bis(β-aminoethyl ether)-*N,N,N′,N′*-tetraacetic acid
ESI: electrospray ionization
GCMS: gas chromatography mass spectrometry
GST: glutathione S-transferase
HBD/HBA: hydrogen-bond donor/hydrogen-bond acceptor
HEPES: 4-(2-hydroxyethyl)-1-piperazineethanesulfonic acid
HPLC: high-pressure liquid chromatography
HRMS: high-resolution mass spectrometry
i.p.: intraperitoneal
i.v.: intravenous
LCMS: liquid chromatography mass spectrometry
NBS: *N*-bromosuccinimide
NCS: *N*-chlorosuccinimide
PARP: poly(ADP-ribose) polymerase
PD: pharmacodynamic
PK: pharmacokinetic
p.o.: oral
*R*_f_: retention factor
RNAP II: RNA polymerase II
SAR: structure–activity relationship
sat.: saturated
SEC: size exclusion chromatography
SMM: small molecule microarray
TEA: triethanolamine
TFA: trifluoroacetic acid
THF: tetrahydrofuran
TLC: thin-layer chromatography
