# Supplementary material for: Discovery of KB-0742, a Potent, Selective, Orally Bioavailable Small Molecule Inhibitor of CDK9 for MYC-Dependent Cancers
Source: J Med Chem. 2023 Nov 15;66(23):15629–47. doi: 10.1021/acs.jmedchem.3c01233 (PMC10726352; doi:10.1021/acs.jmedchem.3c01233)
Supplement: Supplementary file 1 — jm3c01233_si_001.pdf [file jm3c01233_si_001.pdf]

Supporting Information

Discovery of KB-0742, a potent, selective, orally bioavailable small molecule inhibitor of CDK9 for MYC-dependent cancers

David B. Freeman*[,1], Tamara D. Hopkins,[1] Peter J. Mikochik,[1] Joseph P. Vacca,[1] Hua Gao,[1] Adel Naylor-Olsen,[3] Sonali Rudra,[4] Huixu Li,[5] Marius S. Pop,[1] Rosa A. Villagomez,[1] Christina Lee,[1] Heng Li,[1] Minyun Zhou,[1] Douglas C. Saffran,[1] Nathalie Rioux,[2] Tressa R. Hood,[1] Melinda A. L. Day,[1] Michael R. McKeown,[1] Charles Y. Lin, [1]Norbert Bischofberger[1] and B. Wesley Trotter[1]

[1]Kronos Bio, Inc., 301 Binney Street, 2nd Floor East, Cambridge, MA 02142 and 1300 So. El Camino Real Suite 400 San Mateo, CA 94402

[2]Certara Strategic Consulting, 100 Overlook Center, Suite 101, Princeton, NJ 08540

[3]Naylor Olsen Consulting, LLC, 3369 Saddle Wood Court, Lansdale, PA 19446

[4]TCG Lifesciences Private Limited, Block BN, Plot 7, Salt-lake Electronics Complex, Sector V, Kolkata 700091, West Bengal, India [5]WuXi AppTec (Tianjin) Co., Ltd., 168 NanHai Road, 10th Avenue, TEDA, Tianjin 300457, P. R. China

Corresponding Author

David B. Freeman — Kronos Bio, Inc. Cambridge, Massachusetts 02142, United States; orcid.org/0000-0002-6826-2272; Phone: (857) 856-5773; Email: dbf@kronosbio.com

Table of Contents — Page

Title page — **S1**

Cocrystal structure of compound **28** with CDK9/cyclin T1 — **S2**

Synthesis of supporting reagents and intermediates — **S5**

Exemplary modeling of compounds 2, 4, and 6 showcasing interactions with hinge residue Cys106 and Asp109 impacting biochemical potency — **S10**

Kinome panel upon compound **28** treatment using Kinase HotSpot™ Profiler — **S11**

Stereochemical effect on CDK selectivity among analogues **28**, **38**, **39**, and **40** — **S32**

Viability effects of **28** on TNBC cell lines **S32**

OncoPanel^{TM} Multiplexed Cytotoxicity Assay control compound **S33**

TNBC PDX models treated with **28** **S34**

Compound **28** spectral characterization and purity **S35**

References **S37**

X-ray crystallography

The CDK9 and CyclinT1 constructs were designed as reported[1]. GST-TEV-CDK9 (M1-T330, S7D, V8N, K44R, Y138F, K280A, D307E, N311E) and His-Flag-TEV-CyclinT1 (M1-R259, R26A, Q77R, E96G, K106R, F241L) were cloned into pFastBac1 vector. The baculovirus of CDK9 and CyclinT1 was added at a 1:1 ratio to infect SF21 cells to express the CDK9/Cyclin T1 complex. The cell pallet containing protein complex was loaded onto Nickel-NTA column and then GST column followed by TEV cleavage to remove GST-tag on CDK9 and His tag on Cyclin T1. The protein complex was further separated through reverse Nickel and GST column and purified by SEC column (Superdex 200 Increase 10/300 GL) in a final buffer 20 mM Tris-HCl (pH 8.0), 500 mM NaCl, 5 mM DTT. The protein complex was concentrated to 5.66 mg/ml and incubated with KB130742 on ice for 1 hr before setting up crystallization trays. The co-crystals of CDK9-Cyclin T1-130742 were crystallized from 0.2 M K3 citrate, 20% PEG 3350 and harvested and flash cooled in liquid nitrogen for data collection. A 3.75 Å dataset was collected on beamline BL45XU at Spring-8 synchrotron source and were indexed and integrated with XDS[2] and scaled by aimless. The CDK9-CyclinT1 complex structure was solved by molecular replacement with

Phaser module in CCP4 package suite[3], using the coordinates with PDB code 3BLH as the search model. The compound 130742 was placed into the electron density based on the difference electron density maps. After multi-cycles of iterative refinement with Refmac module in CCP4 package suite and manually adjustment in Coot program[4], the complex structure is finally refined to R_{work}/R_{free} as 19.85%/24.99%, respectively. The refinement statistics are listed in the table below.

Table S1. X-ray crystallography table data collection and refinement statistics

**Crystal**	1H637
**Data collection**	
Diffraction source	Spring-8, BL45XU
Wavelength	0.99999
Space group	H3
**Unit cell dimensions**	
a, b, c (Å)	171.50 171.50 96.12
α, β, γ, (°)	90.00 90.00 120.00
Resolution (Å)	85.75-3.75 (4.19-3.75)
Unique reflections	10792 (3092)
Completeness (%)	99.7 (100)
Redundancy	4.7 (4.9)
R_{merge}(%)	6.2 (68.3)

I/σ (I)	11.6 (2.3)
CC1/2	0.999 (0.816)
**Refinement**	
Resolution (Å)	85.75-3.75 (4.19-3.75)
No. of reflections	10252
$R_{work/}$ R_{free} (%)	19.85/24.99
**No. of atoms**	
Protein	4537
Ion/Ligand	21
Water	0
**B-factors**	
Protein	154.822
Ion/Ligand	0.500
Water	0
**R.m.s deviations**	
Bond lengths (Å)	0.0025
Bond angles (º)	0.8628
**Ramachandran plot (%)**	
Most favored	91.18

Allowed	6.06
Disallowed	2.76
**Rotamer**	
Most favored	87.20%
Allowed	9.6
Disallowed	3.20

* Statistics for the highest-resolution shell are shown in parentheses.

Synthesis of supporting reagents and intermediates

Scheme S1

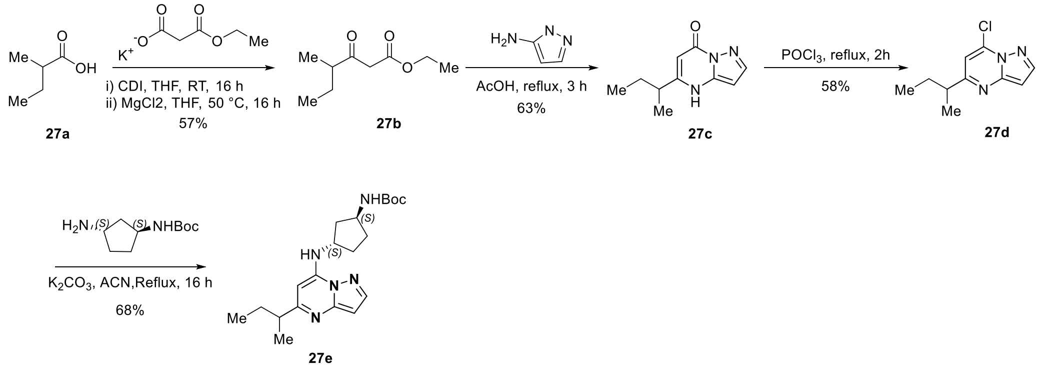

*ethyl 4-methyl-3-oxo-hexanoate (**27b**).* 2-Methylbutanoic acid **27a** (5 g, 49.0 mmol) was dissolved in THF (100 mL) and cooled to 0 °C. Within 20 min CDI (12.3 g, 75.88 mmol) were added portion-wise. The temp was allowed to rise to rt and the mixture was stirred at rt for overnight. In another flask $MgCl_2$ (4.66 g, 49.9 mmol) and potassium 3-ethoxy-3-oxo-propanoate (12.9 g, 75.9 mmol) were mixed with THF (100 mL) and stirred under argon over night at 50 °C. The white suspension was cooled to rt and the solution that was left overnight was added dropwise within 10 min and the reaction mixture was stirred 16 h at rt. (Note: Several minutes after the addition a difficult to stir chewing gum deposits, and after several hours the reaction mixture becomes easier to stir again). The reaction mixture was concentrated to about a third, taken up in half saturated (sat.) potassium bisulphate solution and extracted twice with ethyl acetate. Then organic layers were washed with sat. sodium bicarbonate solution, combined, dried over anhyd Na_2SO_4 and evaporated. The crude residue was purified by Combiflash column chromatography (silica gel 230–400

mesh), eluent hexane-ethyl acetate to give *ethyl 4-methyl-3-oxo-hexanoate* ***27b*** (4.8 g, 27.9 mmol, 56.9% yield) as a transparent liquid.

*5-sec-butyl-4H-pyrazolo[1,5-a]pyrimidin-7-one (**27c**).* A mixture of compound **27b** (2 g, 11.6 mmol), and 1H-pyrazol-5-amine (965mg, 11.6 mmol), in acetic acid (8 mL) were heated under reflux for 3 h and the solvent was evaporated *in vacuo*. The residue was treated with ethyl acetate and filtered to give compound **27c** (1.4 g, 7.32 mmol, 63.0% yield) as a white solid. LCMS (ESI) *m/z*: 191.9 $[M + H]^+$.

*7-chloro-5-sec-butyl-pyrazolo[1,5-a]pyrimidine (**27d**).* A stirred solution of compound **27c** (1.3 g, 6.8 mmol) in $POCl_3$ (12.7 mL, 136 mmol) was heated to reflux for 2 h. The reaction mixture was cooled to rt, and the excess reagent was removed *in vacuo.* The residue was treated with ice-water, and the chlorinated product was extracted from the aqueous mixture by DCM. The organic layer was separated, dried over anhyd Na_2SO_4 and purified by Combiflash column chromatography (silica gel 230–400 mesh) eluent 10-20% ethyl acetate in hexanes to give compound **27d** (830 mg, 3.96 mmol, 58.2% yield) as a light-yellow sticky liquid. LCMS (ESI) *m/z*: 210.1 $[M + H]^+$.

*tert-butyl N-[(1S,3S)-3-[(5-sec-butylpyrazolo[1,5-a]pyrimidin-7-yl)amino]cyclopentyl]carbamate (**27e**).* To a stirred solution of compound **27d** (90 mg, 0.430 mmol), *tert*-Butyl ((1*S*,3*S*)-3-aminocyclopentyl)carbamate (103.2 mg, 0.520 mmol) and K_2CO_3 (178 mg, 1.29 mmol) in MeCN (10 mL) were heated to reflux for 16 h. The reaction mixture was filtered, concentrated *in vacuo* and purified by Combiflash column chromatography (silica gel, 230–400 mesh) to give compound **27e** (110 mg, 0.295 mmol, 68.6% yield) as a white solid. LCMS (ESI) *m/z*: 374.0 $[M + H]^+$.

Scheme S2

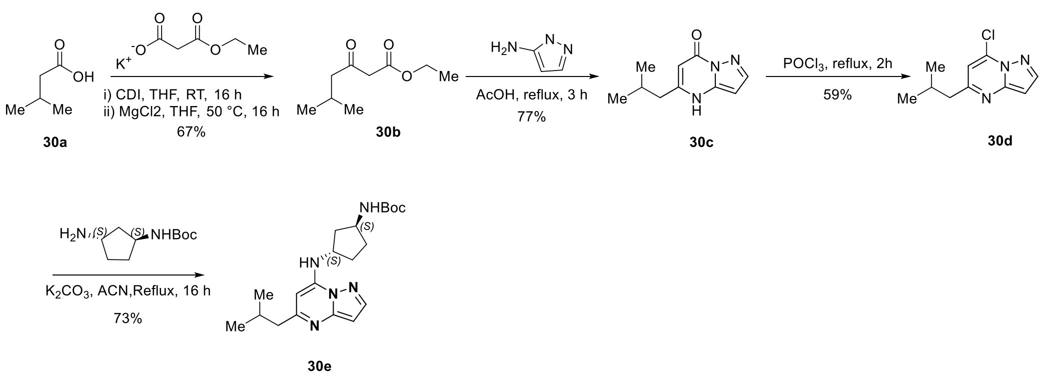

*ethyl 5-methyl-3-oxo-hexanoate (**30b**)*. 3-Methylbutanoic acid **30a** (4 g, 39.1 mmol) was dissolved in THF (80 mL) and cooled to 0 °C. Within 20 min CDI (9.84 g, 60.7 mmol) was added portion-wise. The temperature was allowed to rise to rt and the mixture was stirred at rt for 16 h. In another flask $MgCl_2$ (3.73 g, 39.2 mmol) and potassium 3-ethoxy-3-oxo-propanoate (10.3 g, 60.7 mmol) were mixed in THF (80 mL) and stirred under argon for 16 h at 50 °C. The white suspension was cooled to rt and the solution that was stirred for 16 h was added dropwise within 10 min and the reaction mixture was stirred again for 16 h at rt. (note: After the addition after several minutes a difficult to stir chewing gum deposits, and after several hours the reaction mixture becomes easier to stir again). The reaction mixture was concentrated to about a third, taken up in sat. potassium bisulphate solution and extracted twice with ethyl acetate. Then the combined organic layers were washed with sat. sodium bicarbonate solution, dried over anhyd sodium sulfate and evaporated in vacuo. The crude residue was purified by Combiflash column chromatography (silica gel, 230–400 mesh) eluent 10-20% ethyl acetate in hexanes to give *ethyl 5-methyl-3-oxo-hexanoate* **30b** (4.5 g, 26.1 mmol, 66.7% yield) as a yellow liquid. LCMS (ESI) m/z: 173.1 $[M + H]^+$

*5-isobutyl-4H-pyrazolo[1,5-a]pyrimidin-7-one (**30c**).* A mixture of ethyl 5-methyl-3-oxo-hexanoate (1 g, 5.81 mmol) and 1H-pyrazol-5-amine (0.48 g, 5.81 mmol) in acetic acid (10 mL) was heated under reflux for 3 h. The solvent was evaporated *in vacuo* and the residue was treated with ethyl acetate and filtered to give compound **30c** (0.85g, 4.45 mmol, 76.6% yield) as an off-white solid. LCMS (ESI) *m/z*: 192.0 $[M + H]^+$.

*7-chloro-5-isobutyl-pyrazolo[1,5-a]pyrimidine (**30d**).* A stirred solution of compound **30c** (0.8 g, 4.18 mmol) in $POCl_3$ (7.82 mL, 83.7 mmol) was heated to reflux for 2 h. The reaction mixture was cooled to rt, and the excess reagent was removed *in vacuo*. The residue was treated with ice-water, and the chlorinated product was extracted from the aqueous mixture by DCM. The organic layers was combined, dried over anhyd sodium sulfate and purified by Combiflash chromatography, eluent 10% ethyl acetate in hexanes to give compound **30d** (520 mg, 2.48 mmol, 59.3% yield) as a light yellow liquid. LCMS (ESI) *m/z*: 210.0 $[M + H]^+$.

tert-butyl N-[(1S,3S)-3-[(5-isobutylpyrazolo[1,5-a]pyrimidin-7-yl)amino]cyclopentyl]carbamate ***(30e).*** A mixture of compound **30d** (100 mg, 0.480 mmol), *tert*-Butyl ((1*S*,3*S*)-3-aminocyclopentyl)carbamate (115 mg, 0.570 mmol) and K_2CO_3 (197 mg, 1.43 mmol) in MeCN (10 mL) were heated to reflux for 16 h. The reaction mixture was filtered, concentrated *in vacuo* and purified by Combiflash column chromatography (silica gel, 230–400 mesh), eluent 30% ethyl acetate in hexane to give compound **30e** (130 mg, 0.348 mmol, 73.0% yield) as a yellow sticky liquid. LCMS (ESI) *m/z:* 373.9 $[M + H]^+$.

Scheme S3

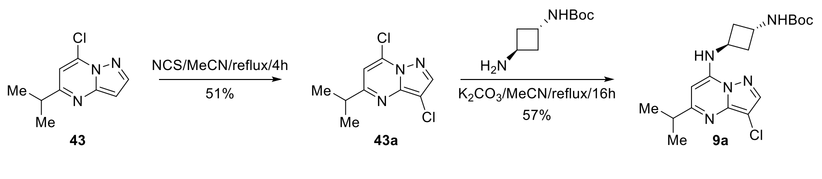

3,7-dichloro-5-isopropyl-pyrazolo[1,5-a]pyrimidine ***(43a)****.* To a stirred solution of 7-chloro-5-isopropyl-pyrazolo[1,5-a]pyrimidine **43** (500 mg, 2.56 mmol) in MeCN (10 mL) was added NCS (512 mg, 3.83 mmol) at 0 °C. The resulting mixture was refluxed for 4 h. The reaction mixture was concentrated *in vacuo*. The crude was purified by Combiflash column chromatography (silica gel, 230–400 mesh), eluent 10% ethyl acetate in hexanes, to give compound **43a** (300 mg,1.30 mmol, 51.0% yield) as a light yellow solid. LCMS (ESI) *m/z*: 229.9 $[M + H]^+$.

tert-butyl-((1r,3r)-3-((3-chloro-5-isopropylpyrazolo[1,5-a]pyrimidin-7-yl)amino)cyclobutyl)carbamate ***(9a)****.* A mixture of compound **43a** (75 mg, 0.330 mmol), *tert*-butyl ((1*r*,3*r*)-3-aminocyclobutyl)carbamate (72.9 mg, 0.390 mmol) and K_2CO_3 (135mg, 0.980 mmol) in MeCN (10 mL) were heated to reflux for 16 h. The reaction mixture was filtered and concentrated *in vacuo.* The crude product was purified by Combiflash column chromatography (silica gel, 230–400 mesh), eluent 30% ethyl acetate in hexanes to give compound **9a** (70 mg, 0.184 mmol, 56.5% yield) as a yellow liquid. LCMS (ESI) *m/z*: 379.7 $[M + H]^+$.

Scheme S4

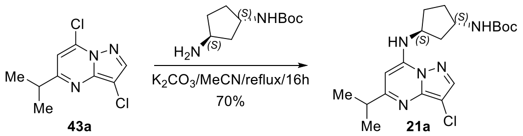

tert-butyl-N-[(1S,3S)-3-[(3-chloro-5-isopropyl-pyrazolo[1,5-a]pyrimidin-7-yl)amino]cyclopentyl]carbamate ***(21a)****.* A mixture of **43a** (75 mg, 0.330 mmol), *tert*-butyl ((1*S*,3*S*)-3-aminocyclopentyl)carbamate (78.3 mg, 0.390 mmol) and K_2CO_3 (135 mg, 0.980 mmol) in MeCN (10 mL) were heated to reflux for 16 h. The reaction mixture was filtered, concentrated *in vacuo* and purified by Combiflash chromatography (silica gel, 230–400 mesh), eluent 30% ethyl acetate in hexanes to give compound **21a** (90 mg,0.229 mmol, 70.1% yield) as a clear liquid. LCMS (ESI) *m/z*: 393.8 $[M + H]^+$.

Scheme S5

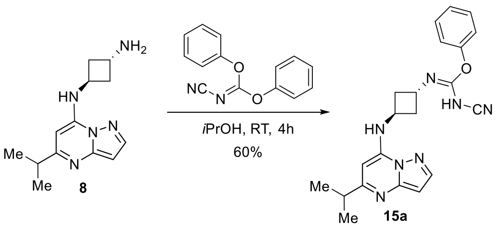

(1r,3r)-1-cyano-3-[3-[(5-isopropylpyrazolo[1,5-a]pyrimidin-7-yl)amino]cyclobutyl]-2-phenyl-isourea ***(15a)****.* To a stirred solution of compound **8** (100 mg, 0.410 mmol) in isopropyl alcohol (5 mL), diphenyl N-cyanocarbonimidate (146 mg, 0.610 mmol) was added and the reaction mixture was stirred for 4 h at rt. After completion of the reaction as monitored by TLC, solvent was

evaporated *in vacuo* and the crude was purified by Combiflash column chromatography (silica, 230–400 mesh), eluting with 70% ethyl acetate in hexanes to give compound **15a** (95 mg, 0.244 mmol, 59.8% yield) as a light-yellow solid. LCMS (ESI) *m/z*: 390.2 $[M + H]^+$.

Scheme S6

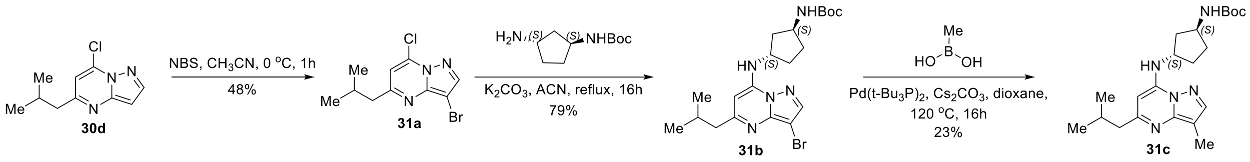

*3-bromo-7-chloro-5-isobutyl-pyrazolo[1,5-a]pyrimidine (**31a**).* To a stirred solution of compound **30d** (300 mg, 1.43 mmol) in MeCN (5.6 mL) was added NBS (304 mg, 1.72 mmol) at 0 °C. The resulting mixture was stirred at 0 °C for 1 h. The reaction mixture was concentrated *in vacuo* and the crude was purified by Combiflash column chromatography (silica, 230–400 mesh), eluting with 10% ethyl acetate in hexanes to give compound **31a** (200 mg, 0.693 mmol, 48.4% yield) as a ligh- yellow solid. LCMS (ESI) *m/z*: 288.0 $[M + H]^+$.

*tert-butyl-N-[(1S,3S)-3-[(3-bromo-5-isobutyl-pyrazolo[1,5-a]pyrimidin-7-yl)amino]cyclopentyl]carbamate (**31b**).* A stirred solution of compound **31a** (200 mg, 0.690 mmol), *tert*-butyl ((1*S*,3*S*)-3-aminocyclopentyl)carbamate (153 mg, 0.760 mmol) and K_2CO_3 (0.29 g, 2.08 mmol) in MeCN (2.61 mL) was heated to reflux for 16 h. The reaction mixture was filtered and evaporated in vacuo to give compound **31b** (250 mg, 0.547 mmol, 79.0% yield) as a red color sticky solid. LCMS (ESI) *m/z*: 452.0 $[M + H]^+$.

*tert-butyl-N-[(1S,3S)-3-[(5-isobutyl-3-methyl-pyrazolo[1,5-a]pyrimidin-7-yl)amino]cyclopentyl]carbamate (**31c**).* A stirred solution of compound **31b** (300 mg, 0.660 mmol), cesium carbonate (1512 mg, 4.64 mmol) and methyl boronic acid (0.44 mL, 6.63 mmol) in 1,4-dioxane (14.8 mL) in a sealed tube was degassed for 15 min with Argon. Then to the mixture were added bis(tri-*tert*-butylphosphine)palladium(0) (50.8 mg, 0.100 mmol) and degassed for 5 min and heated at 120 °C for 16 h. After completion of the reaction, solvent was evaporated and quenched with water (10 mL). The aqueous layer was extracted with DCM (10 mL) and the combined organic layers were washed with brine (10 mL), dried over anhyd Na_2SO_4 and evaporated in vacuo. The crude was purified by Combiflash column chromatography (silica, 230–400 mesh), eluting with 20-25% ethyl acetate in hexanes to give compound **31c** (60 mg, 0.151 mmol, 22.8% yield) as an off white solid. LCMS (ESI) *m/z*: 388.6 $[M + H]^+$.

Scheme S7

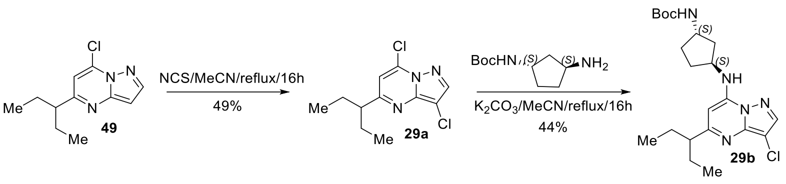

*3,7-dichloro-5-(1-ethylpropyl)pyrazolo[1,5-a]pyrimidine (**29a**).* To a stirred solution of compound **49** (180 mg, 0.800 mmol) in MeCN (6.75 mL) was added NCS (135 mg, 1.01 mmol) and the reaction mixture was stirred for 18 h at rt. The solvent was removed *in vacuo* and the crude was purified by Combiflash column chromatography (silica, 230–400 mesh), eluting with at 3% ethyl acetate in hexanes to give compound **29a** as a pale color syrup. LCMS (ESI) *m/z*: 258.2 $[M + H]^+$.

*tert-butyl-N-[(1S,3S)-3-[[3-chloro-5-(1-ethylpropyl)pyrazolo[1,5-a]pyrimidin-7-yl]amino]cyclopentyl]carbamate (**29b**).* A stirred solution of compound **29a** (110 mg, 0.430 mmol), *tert*-butyl ((1*S*,3*S*)-3-aminocyclopentyl)carbamate (93.9 mg, 0.470 mmol) and K_2CO_3 (0.18 g, 1.28 mmol) in MeCN (1.5 mL) were heated to reflux for 16 h. The reaction mixture was filtered, concentrated under reduced pressure to give the crude. The crude was purified by Combiflash column chromatography (silica, 230–400 mesh), eluting with 25-30% of ethyl acetate in hexanes to give compound **29b** (80 mg, 0.186 mmol, 43.8% yield) as an off- white solid. LCMS (ESI) *m/z*: 422.2 $[M + H]^+$.

Figure S1. Exemplary modeling of compounds 2, 4, and 6 showcasing interactions with hinge residue Cys106 and Asp109 impacting biochemical potency

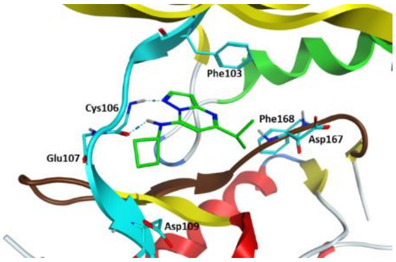

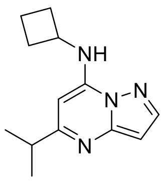

2
CDK9/Cyclin T1
Biochemical IC_{50} = 95

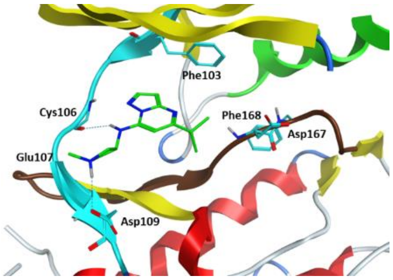

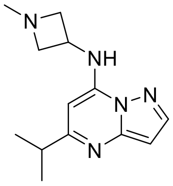

4
CDK9/Cyclin T1
Biochemical IC_{50} = 2815

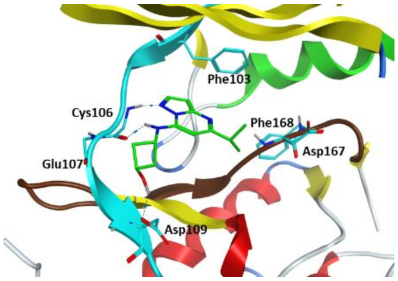

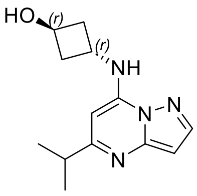

6
CDK9/Cyclin T1
Biochemical IC_{50} = 183

Table S2. Kinome panel upon compound 28 treatment using Kinase HotSpot™ Profiler

Kinases	% Enzyme Activity (relative to DMSO controls) KB-00130742-003-001 (10uM)		IC50 (M) Control Cmpd	Control Cmpd ID
	Data 1	Data 2		
**ABL1**	105.33	104.68	**3.12E-08**	**STAUROSPORINE**
**ABL2/ARG**	97.94	97.71	**1.34E-08**	**STAUROSPORINE**
**ACK1**	71.28	70.51	**1.83E-08**	**STAUROSPORINE**
**AKT1**	103.95	103.25	**6.83E-09**	**STAUROSPORINE**
**AKT2**	93.43	91.18	**2.48E-08**	**STAUROSPORINE**
**AKT3**	86.11	85.67	**2.47E-09**	**STAUROSPORINE**
**ALK**	108.89	108.42	**2.44E-09**	**STAUROSPORINE**
**ALK1/ACVRL1**	100.68	100.45	**2.14E-08**	**LDN193189**
**ALK2/ACVR1**	100.21	96.23	**5.64E-08**	**LDN193189**
**ALK3/BMPR1A**	102.56	101.54	**6.49E-08**	**LDN193189**
**ALK4/ACVR1B**	95.27	94.72	**1.89E-07**	**LDN193189**
**ALK5/TGFBR1**	99.01	97.52	**5.81E-07**	**LDN193189**
**ALK6/BMPR1B**	102.26	101.68	**1.10E-08**	**LDN193189**
**ARAF**	90.50	87.11	**1.96E-08**	**GW5074**
**ARK5/NUAK1**	95.76	94.22	**8.57E-10**	**STAUROSPORINE**
**ASK1/MAP3K5**	99.53	98.22	**1.88E-08**	**STAUROSPORINE**
**Aurora A**	102.75	101.16	**1.57E-09**	**STAUROSPORINE**
**AURORA B**	104.95	104.20	**9.37E-09**	**STAUROSPORINE**
**Aurora C**	107.52	106.14	**4.20E-10**	**STAUROSPORINE**
**AXL**	98.87	95.64	**2.20E-09**	**STAUROSPORINE**
**BLK**	100.12	95.30	**1.49E-09**	**STAUROSPORINE**
**BMPR2**	83.68	80.86	**7.46E-08**	**STAUROSPORINE**
**BMX/ETK**	102.39	98.56	**7.79E-09**	**STAUROSPORINE**
**BRAF**	101.36	98.84	**1.22E-08**	**GW5074**
**BRK**	76.52	76.16	**2.29E-07**	**STAUROSPORINE**
**BRSK1**	109.61	108.90	**5.06E-10**	**STAUROSPORINE**

**BRSK2**	99.85	98.21	**7.85E-10**	**STAUROSPORINE**
**BTK**	101.06	100.75	**1.65E-08**	**STAUROSPORINE**
**c-Kit**	85.70	85.26	**1.18E-09**	**STAUROSPORINE**
**c-MER**	103.32	101.02	**1.34E-08**	**STAUROSPORINE**
**c-MET**	105.73	98.93	**1.54E-07**	**STAUROSPORINE**
**c-Src**	99.84	98.27	**1.87E-09**	**STAUROSPORINE**
**CAMK1a**	96.23	95.95	**2.19E-09**	**STAUROSPORINE**
**CAMK1b**	97.65	97.42	**7.16E-09**	**STAUROSPORINE**
**CAMK1d**	95.89	95.66	**4.06E-10**	**STAUROSPORINE**
**CAMK1g**	95.49	90.65	**2.71E-09**	**STAUROSPORINE**
**CAMK2a**	97.49	96.66	**6.65E-11**	**STAUROSPORINE**
**CAMK2b**	94.33	93.19	**8.92E-11**	**STAUROSPORINE**
**CAMK2d**	103.83	101.48	**6.31E-11**	**STAUROSPORINE**
**CAMK2g**	100.30	95.05	**5.30E-10**	**STAUROSPORINE**
**CAMK4**	96.99	95.66	**1.14E-07**	**STAUROSPORINE**
**CAMKK1**	95.88	94.68	**4.90E-08**	**STAUROSPORINE**
**CAMKK2**	93.75	91.22	**3.16E-08**	**STAUROSPORINE**
**CDC7/DBF4**	101.17	99.69	**1.96E-08**	**STAUROSPORINE**
**CDK1/cyclin A**	45.03	44.50	**2.84E-09**	**STAUROSPORINE**
**CDK1/cyclin B**	46.05	44.91	**1.48E-09**	**STAUROSPORINE**
**CDK1/cyclin E**	41.61	39.86	**3.12E-09**	**STAUROSPORINE**
**CDK14/cyclin Y (PFTK1)**	73.73	71.62	**1.39E-07**	**STAUROSPORINE**
**CDK16/cyclin Y (PCTAIRE)**	31.88	30.96	**1.28E-08**	**STAUROSPORINE**
**CDK17/cyclin Y (PCTK2)**	32.94	31.54	**1.24E-08**	**STAUROSPORINE**
**CDK18/cyclin Y (PCTK3)**	22.58	22.52	**2.05E-08**	**STAUROSPORINE**
**CDK19/cyclin C**	85.40	83.32	**2.98E-10**	**STAUROSPORINE**
**CDK2/cyclin A**	42.19	41.15	**7.18E-10**	**STAUROSPORINE**
**CDK2/Cyclin A1**	50.15	49.75	**1.63E-09**	**STAUROSPORINE**
**CDK2/cyclin E**	51.58	50.29	**2.17E-09**	**STAUROSPORINE**
**CDK2/cyclin E2**	59.69	59.36	**1.86E-09**	**STAUROSPORINE**
**CDK2/cyclin O**	16.18	15.84	**1.45E-09**	**STAUROSPORINE**
**CDK3/cyclin E**	27.53	26.73	**1.35E-09**	**STAUROSPORINE**

**CDK3/cyclin E2**	23.43	23.18	**1.32E-09**	**STAUROSPORINE**
**CDK4/cyclin D1**	31.65	31.57	**1.32E-08**	**STAUROSPORINE**
**CDK4/cyclin D3**	40.14	39.85	**2.33E-08**	**STAUROSPORINE**
**CDK5/P25**	71.04	69.65	**1.90E-09**	**STAUROSPORINE**
**CDK5/p35**	72.42	72.30	**1.82E-09**	**STAUROSPORINE**
**CDK6/cyclin D1**	70.44	70.28	**2.07E-09**	**STAUROSPORINE**
**CDK6/cyclin D3**	31.73	30.00	**1.40E-08**	**STAUROSPORINE**
**CDK7/cyclin H**	35.53	35.50	**3.97E-08**	**STAUROSPORINE**
**CDK8/cyclin C**	92.27	92.21	**3.97E-10**	**STAUROSPORINE**
**CDK9/cyclin K**	-0.41	-0.66	**1.28E-08**	**STAUROSPORINE**
**CDK9/cyclin T1**	-0.12	-0.15	**7.03E-09**	**STAUROSPORINE**
**CDK9/cyclin T2**	1.70	0.60	**3.53E-09**	**STAUROSPORINE**
**CHK1**	117.59	112.89	**7.92E-11**	**STAUROSPORINE**
**CHK2**	95.25	94.35	**6.59E-09**	**STAUROSPORINE**
**CK1a1**	108.47	105.77	**5.95E-06**	**STAUROSPORINE**
**CK1a1L**	103.28	102.84	**2.02E-06**	**STAUROSPORINE**
**CK1d**	104.88	101.35	**2.39E-07**	**D4476**
**CK1epsilon**	104.06	103.31	**3.02E-07**	**D4476**
**CK1g1**	94.26	93.63	**6.54E-06**	**STAUROSPORINE**
**CK1g2**	92.59	91.26	**2.41E-06**	**STAUROSPORINE**
**CK1G3**	108.30	107.47	**3.45E-06**	**STAUROSPORINE**
**CK2a**	95.74	91.64	**1.43E-07**	**GW5074**
**CK2a2**	95.04	94.03	**5.78E-07**	**STAUROSPORINE**
**CLK1**	97.69	94.88	**7.18E-09**	**STAUROSPORINE**
**CLK2**	85.94	83.47	**3.73E-09**	**STAUROSPORINE**
**CLK3**	100.14	93.81	**2.15E-06**	**STAUROSPORINE**
**CLK4**	101.12	98.65	**4.16E-08**	**STAUROSPORINE**
**COT1/MAP3K8**	76.80	75.25	**6.36E-06**	**RO-31-8220**
**CSK**	100.45	96.93	**2.12E-08**	**STAUROSPORINE**
**CTK/MATK**	98.39	95.91	**2.03E-07**	**STAUROSPORINE**
**DAPK1**	99.09	98.01	**1.20E-08**	**STAUROSPORINE**
**DAPK2**	100.11	99.91	**8.37E-09**	**STAUROSPORINE**

**DCAMKL1**	83.01	82.87	**5.74E-08**	**STAUROSPORINE**
**DCAMKL2**	79.52	78.94	**1.11E-08**	**STAUROSPORINE**
**DDR1**	96.11	94.34	**4.12E-09**	**STAUROSPORINE**
**DDR2**	94.15	93.86	**4.85E-10**	**STAUROSPORINE**
**DLK/MAP3K12**	91.13	89.17	**4.17E-07**	**STAUROSPORINE**
**DMPK**	113.29	112.93	**5.05E-08**	**STAUROSPORINE**
**DMPK2**	98.44	96.34	**1.36E-09**	**STAUROSPORINE**
**DRAK1/STK17A**	100.09	99.15	**3.13E-08**	**STAUROSPORINE**
**DYRK1/DYRK1A**	100.10	98.57	**2.87E-09**	**STAUROSPORINE**
**DYRK1B**	88.60	88.45	**8.73E-10**	**STAUROSPORINE**
**DYRK2**	102.09	97.74	**1.08E-07**	**STAUROSPORINE**
**DYRK3**	99.13	96.65	**1.94E-08**	**STAUROSPORINE**
**DYRK4**	101.18	98.56	**4.68E-06**	**GW5074**
**EGFR**	104.29	103.98	**6.93E-08**	**STAUROSPORINE**
**EPHA1**	97.39	94.42	**1.20E-07**	**STAUROSPORINE**
**EPHA2**	101.84	101.62	**7.19E-08**	**STAUROSPORINE**
**EPHA3**	102.22	101.96	**2.79E-08**	**STAUROSPORINE**
**EPHA4**	103.24	101.94	**1.86E-08**	**STAUROSPORINE**
**EPHA5**	94.88	93.05	**6.15E-08**	**STAUROSPORINE**
**EPHA6**	104.40	103.58	**2.22E-08**	**STAUROSPORINE**
**EPHA7**	100.34	98.17	**3.43E-08**	**STAUROSPORINE**
**EPHA8**	102.69	102.47	**1.13E-07**	**STAUROSPORINE**
**EPHB1**	89.22	88.82	**4.96E-08**	**STAUROSPORINE**
**EPHB2**	97.60	94.92	**1.16E-07**	**STAUROSPORINE**
**EPHB3**	90.73	90.21	**1.31E-06**	**STAUROSPORINE**
**EPHB4**	89.33	86.09	**1.42E-07**	**STAUROSPORINE**
**ERBB2/HER2**	88.78	86.76	**4.11E-08**	**STAUROSPORINE**
**ERBB4/HER4**	96.83	94.89	**1.84E-07**	**STAUROSPORINE**
**ERK1**	96.45	96.41	**5.28E-09**	**SCH772984**
**ERK2/MAPK1**	89.26	88.64	**8.97E-10**	**SCH772984**
**ERK5/MAPK7**	100.81	99.67	**1.34E-05**	**STAUROSPORINE**
**ERK7/MAPK15**	83.30	81.04	**9.80E-09**	**STAUROSPORINE**

**ERN1/IRE1**	81.07	80.74	**7.79E-08**	**STAUROSPORINE**
**ERN2/IRE2**	104.51	104.28	**4.92E-08**	**STAUROSPORINE**
**FAK/PTK2**	91.83	90.05	**1.05E-08**	**STAUROSPORINE**
**FER**	80.55	79.12	**7.24E-10**	**STAUROSPORINE**
**FES/FPS**	97.77	97.73	**1.46E-09**	**STAUROSPORINE**
**FGFR1**	100.36	99.78	**3.62E-09**	**STAUROSPORINE**
**FGFR2**	96.78	95.21	**1.32E-09**	**STAUROSPORINE**
**FGFR3**	101.45	100.12	**1.91E-08**	**STAUROSPORINE**
**FGFR4**	98.24	97.04	**7.37E-08**	**STAUROSPORINE**
**FGR**	77.67	76.43	**9.28E-10**	**STAUROSPORINE**
**FLT1/VEGFR1**	97.90	96.41	**4.42E-09**	**STAUROSPORINE**
**FLT3**	105.84	105.17	**1.27E-09**	**STAUROSPORINE**
**FLT4/VEGFR3**	98.64	98.40	**1.90E-09**	**STAUROSPORINE**
**FMS**	76.37	73.79	**1.91E-09**	**STAUROSPORINE**
**FRK/PTK5**	102.61	100.92	**2.44E-08**	**STAUROSPORINE**
**FYN**	115.44	106.22	**2.51E-09**	**STAUROSPORINE**
**GCK/MAP4K2**	97.34	96.08	**3.82E-10**	**STAUROSPORINE**
**GLK/MAP4K3**	101.40	100.57	**3.61E-10**	**STAUROSPORINE**
**GRK1**	101.96	97.48	**3.49E-08**	**STAUROSPORINE**
**GRK2**	112.26	109.31	**1.25E-06**	**STAUROSPORINE**
**GRK3**	97.43	95.87	**5.81E-07**	**STAUROSPORINE**
**GRK4**	89.54	88.38	**5.88E-08**	**STAUROSPORINE**
**GRK5**	99.63	98.26	**4.46E-08**	**STAUROSPORINE**
**GRK6**	101.52	98.97	**4.23E-08**	**STAUROSPORINE**
**GRK7**	98.05	96.95	**5.87E-09**	**STAUROSPORINE**
**GSK3a**	99.88	99.62	**6.47E-09**	**STAUROSPORINE**
**GSK3b**	97.38	96.70	**4.81E-09**	**STAUROSPORINE**
**Haspin**	98.43	96.39	**1.10E-08**	**STAUROSPORINE**
**HCK**	90.88	88.73	**1.55E-09**	**STAUROSPORINE**
**HGK/MAP4K4**	69.17	65.92	**3.52E-10**	**STAUROSPORINE**
**HIPK1**	99.43	98.99	**1.38E-06**	**RO-31-8220**
**HIPK2**	92.87	91.23	**8.65E-07**	**STAUROSPORINE**

**HIPK3**	90.05	87.45	**1.17E-06**	**STAUROSPORINE**
**HIPK4**	98.17	98.01	**3.54E-07**	**STAUROSPORINE**
**HPK1/MAP4K1**	91.01	88.00	**3.25E-08**	**RO-31-8220**
**IGF1R**	96.38	95.63	**3.61E-08**	**STAUROSPORINE**
**IKKa/CHUK**	84.15	83.43	**9.73E-08**	**STAUROSPORINE**
**IKKb/IKBKB**	102.77	98.84	**4.89E-07**	**STAUROSPORINE**
**IKKe/IKBKE**	97.71	93.38	**3.52E-10**	**STAUROSPORINE**
**IR**	110.02	106.45	**1.88E-08**	**STAUROSPORINE**
**IRAK1**	100.12	99.30	**4.41E-08**	**STAUROSPORINE**
**IRAK4**	86.72	82.08	**3.40E-09**	**STAUROSPORINE**
**IRR/INSRR**	101.01	98.44	**8.35E-09**	**STAUROSPORINE**
**ITK**	94.49	90.88	**1.52E-08**	**STAUROSPORINE**
**JAK1**	102.50	100.73	**2.27E-10**	**STAUROSPORINE**
**JAK2**	101.41	99.62	**1.08E-10**	**STAUROSPORINE**
**JAK3**	98.34	96.12	**7.24E-11**	**STAUROSPORINE**
**JNK1**	87.29	86.19	**3.51E-07**	**STAUROSPORINE**
**JNK2**	100.98	100.26	**1.68E-06**	**STAUROSPORINE**
**JNK3**	100.36	98.94	**4.78E-08**	**JNKI VIII**
**KDR/VEGFR2**	98.01	97.06	**9.08E-09**	**STAUROSPORINE**
**KHS/MAP4K5**	79.64	78.53	**2.03E-10**	**STAUROSPORINE**
**KSR1**	101.10	99.49	**7.76E-06**	**STAUROSPORINE**
**KSR2**	100.32	99.62	**4.01E-06**	**STAUROSPORINE**
**LATS1**	99.71	98.08	**8.25E-09**	**STAUROSPORINE**
**LATS2**	114.49	111.85	**3.18E-09**	**STAUROSPORINE**
**LCK**	100.72	98.00	**1.46E-09**	**STAUROSPORINE**
**LCK2/ICK**	95.57	90.61	**4.96E-08**	**STAUROSPORINE**
**LIMK1**	102.66	100.50	**6.47E-10**	**STAUROSPORINE**
**LIMK2**	99.97	99.83	**4.26E-08**	**STAUROSPORINE**
**LKB1**	85.33	80.71	**2.53E-08**	**STAUROSPORINE**
**LOK/STK10**	105.39	105.28	**5.25E-08**	**RO-31-8220**
**LRRK2**	99.58	97.64	**5.86E-09**	**STAUROSPORINE**
**LYN**	91.69	90.72	**6.52E-10**	**STAUROSPORINE**

**LYN B**	103.57	103.48	**3.60E-09**	**STAUROSPORINE**
**MAK**	106.08	104.79	**1.90E-08**	**STAUROSPORINE**
**MAPKAPK2**	106.40	102.50	**9.35E-08**	**STAUROSPORINE**
**MAPKAPK3**	97.48	95.32	**1.52E-06**	**STAUROSPORINE**
**MAPKAPK5/PRAK**	93.99	93.67	**3.31E-07**	**STAUROSPORINE**
**MARK1**	102.15	102.14	**1.04E-10**	**STAUROSPORINE**
**MARK2/PAR-1Ba**	106.99	104.61	**7.53E-11**	**STAUROSPORINE**
**MARK3**	104.92	104.48	**2.78E-10**	**STAUROSPORINE**
**MARK4**	98.02	95.78	**2.39E-10**	**STAUROSPORINE**
**MAST3**	95.81	95.27	**1.52E-06**	**STAUROSPORINE**
**MASTL**	98.69	96.54	**2.51E-08**	**STAUROSPORINE**
**MEK1**	104.02	101.08	**1.97E-08**	**STAUROSPORINE**
**MEK2**	107.01	105.40	**3.59E-08**	**STAUROSPORINE**
**MEK3**	103.12	100.04	**2.21E-08**	**STAUROSPORINE**
**MEK5**	109.20	108.37	**4.43E-08**	**STAUROSPORINE**
**MEKK1**	90.26	87.22	**4.15E-07**	**STAUROSPORINE**
**MEKK2**	74.40	73.89	**3.98E-08**	**STAUROSPORINE**
**MEKK3**	105.66	101.27	**1.70E-08**	**STAUROSPORINE**
**MEKK6**	98.15	96.05	**4.28E-07**	**STAUROSPORINE**
**MELK**	96.43	96.26	**4.90E-10**	**STAUROSPORINE**
**MINK/MINK1**	81.91	77.82	**4.25E-10**	**STAUROSPORINE**
**MKK4**	93.87	91.53	**1.29E-06**	**STAUROSPORINE**
**MKK6**	89.70	86.31	**4.87E-09**	**STAUROSPORINE**
**MKK7**	102.06	101.56	**1.41E-06**	**STAUROSPORINE**
**MLCK/MYLK**	101.13	100.71	**2.78E-08**	**STAUROSPORINE**
**MLCK2/MYLK2**	84.51	84.19	**8.69E-09**	**STAUROSPORINE**
**MLK1/MAP3K9**	108.20	107.44	**9.36E-10**	**STAUROSPORINE**
**MLK2/MAP3K10**	125.24	111.25	**3.39E-09**	**STAUROSPORINE**
**MLK3/MAP3K11**	101.75	100.00	**4.18E-09**	**STAUROSPORINE**
**MLK4**	93.41	93.23	**3.41E-06**	**STAUROSPORINE**
**MNK1**	104.40	96.09	**7.36E-08**	**STAUROSPORINE**
**MNK2**	98.77	98.38	**1.50E-08**	**STAUROSPORINE**

**MRCKa/CDC42BPA**	111.22	110.81	**6.17E-09**	**STAUROSPORINE**
**MRCKb/CDC42BPB**	97.68	96.87	**2.36E-09**	**STAUROSPORINE**
**MSK1/RPS6KA5**	87.16	86.56	**5.09E-10**	**STAUROSPORINE**
**MSK2/RPS6KA4**	92.69	92.33	**1.64E-09**	**STAUROSPORINE**
**MSSK1/STK23**	91.58	90.21	**1.49E-06**	**STAUROSPORINE**
**MST1/STK4**	105.97	105.03	**1.04E-09**	**STAUROSPORINE**
**MST2/STK3**	93.20	93.03	**3.87E-09**	**STAUROSPORINE**
**MST3/STK24**	108.55	103.89	**5.22E-09**	**STAUROSPORINE**
**MST4**	88.01	87.06	**5.03E-09**	**STAUROSPORINE**
**MUSK**	91.26	90.57	**2.66E-09**	**STAUROSPORINE**
**MYLK3**	104.69	102.62	**9.34E-08**	**STAUROSPORINE**
**MYLK4**	101.52	100.62	**5.60E-08**	**STAUROSPORINE**
**MYO3A**	96.66	93.64	**1.97E-08**	**STAUROSPORINE**
**MYO3b**	96.29	95.75	**9.15E-09**	**STAUROSPORINE**
**NEK1**	76.85	76.47	**9.53E-09**	**STAUROSPORINE**
**NEK11**	112.56	111.60	**3.38E-06**	**STAUROSPORINE**
**NEK2**	94.02	94.02	**1.96E-07**	**STAUROSPORINE**
**NEK3**	93.55	93.54	**1.02E-07**	**JNK-IN-7**
**NEK4**	100.89	99.25	**7.08E-08**	**STAUROSPORINE**
**NEK5**	104.75	98.58	**3.27E-08**	**STAUROSPORINE**
**NEK6**	99.03	97.91	**1.24E-05**	**PKR INHIBITOR**
**NEK7**	93.86	92.30	**1.71E-05**	**PKR INHIBITOR**
**NEK8**	99.10	97.93	**1.77E-08**	**STAUROSPORINE**
**NEK9**	94.20	92.31	**6.43E-08**	**STAUROSPORINE**
**NIM1**	96.13	90.47	**1.18E-07**	**STAUROSPORINE**
**NLK**	105.31	102.58	**6.52E-08**	**STAUROSPORINE**
**OSR1/OXSR1**	93.22	92.62	**7.18E-08**	**STAUROSPORINE**
**P38a/MAPK14**	100.39	95.31	**1.79E-08**	**SB202190**
**P38b/MAPK11**	93.98	90.74	**3.38E-08**	**SB202190**
**P38d/MAPK13**	100.04	99.58	**2.36E-07**	**STAUROSPORINE**
**P38g**	99.27	99.07	**6.40E-07**	**STAUROSPORINE**
**p70S6K/RPS6KB1**	93.88	92.95	**3.17E-10**	**STAUROSPORINE**

**p70S6Kb/RPS6KB2**	103.82	102.40	**1.37E-09**	**STAUROSPORINE**
**PAK1**	101.02	99.87	**1.30E-10**	**STAUROSPORINE**
**PAK2**	101.48	100.15	**1.75E-09**	**STAUROSPORINE**
**PAK3**	81.79	78.33	**2.01E-10**	**STAUROSPORINE**
**PAK4**	108.40	105.10	**3.21E-09**	**STAUROSPORINE**
**PAK5**	96.25	95.91	**3.57E-09**	**STAUROSPORINE**
**PAK6**	115.50	112.60	**4.29E-09**	**STAUROSPORINE**
**PASK**	93.61	91.81	**1.70E-08**	**STAUROSPORINE**
**PBK/TOPK**	99.06	96.50	**8.24E-08**	**STAUROSPORINE**
**PDGFRa**	89.76	84.34	**4.88E-10**	**STAUROSPORINE**
**PDGFRb**	95.14	94.19	**1.42E-09**	**STAUROSPORINE**
**PDK1/PDPK1**	93.51	92.49	**7.53E-10**	**STAUROSPORINE**
**PEAK1**	97.64	96.11	**2.51E-09**	**STAUROSPORINE**
**PHKg1**	101.95	99.73	**1.25E-09**	**STAUROSPORINE**
**PHKg2**	96.39	94.38	**1.35E-09**	**STAUROSPORINE**
**PIM1**	107.53	104.78	**1.29E-09**	**STAUROSPORINE**
**PIM2**	111.98	110.19	**2.92E-08**	**STAUROSPORINE**
**PIM3**	109.97	105.05	**1.95E-10**	**STAUROSPORINE**
**PKA**	101.05	100.68	**1.54E-09**	**STAUROSPORINE**
**PKAcb**	101.88	100.29	**1.31E-09**	**STAUROSPORINE**
**PKAcg**	100.02	97.53	**1.94E-09**	**STAUROSPORINE**
**PKCa**	89.49	88.51	**2.00E-10**	**STAUROSPORINE**
**PKCb1**	88.39	87.95	**3.38E-09**	**STAUROSPORINE**
**PKCb2**	105.13	104.32	**1.51E-09**	**STAUROSPORINE**
**PKCd**	95.45	93.17	**4.27E-10**	**STAUROSPORINE**
**PKCepsilon**	103.82	101.33	**1.17E-10**	**STAUROSPORINE**
**PKCeta**	94.32	92.66	**5.74E-10**	**STAUROSPORINE**
**PKCg**	99.87	99.48	**2.50E-10**	**STAUROSPORINE**
**PKCiota**	95.57	94.97	**1.51E-08**	**STAUROSPORINE**
**PKCmu/PRKD1**	83.61	83.40	**1.19E-09**	**STAUROSPORINE**
**PKCnu/PRKD3**	93.97	82.60	**9.69E-10**	**STAUROSPORINE**
**PKCtheta**	107.78	105.62	**1.26E-09**	**STAUROSPORINE**

**PKCzeta**	96.41	95.76	**4.26E-08**	**STAUROSPORINE**
**PKD2/PRKD2**	102.75	101.19	**2.30E-09**	**STAUROSPORINE**
**PKG1a**	101.62	100.22	**1.15E-09**	**STAUROSPORINE**
**PKG1b**	97.19	96.02	**1.69E-09**	**STAUROSPORINE**
**PKG2/PRKG2**	102.01	97.70	**3.60E-09**	**STAUROSPORINE**
**PKMYT1**	104.70	100.50	**3.45E-09**	**STAUROSPORINE**
**PKN1/PRK1**	97.64	96.59	**8.60E-10**	**STAUROSPORINE**
**PKN2/PRK2**	100.00	97.41	**2.35E-09**	**STAUROSPORINE**
**PKN3/PRK3**	101.49	98.72	**2.43E-09**	**STAUROSPORINE**
**PLK1**	97.79	96.85	**1.63E-07**	**STAUROSPORINE**
**PLK2**	96.48	96.38	**2.29E-07**	**STAUROSPORINE**
**PLK3**	94.90	93.70	**5.94E-09**	**BI2536**
**PLK4/SAK**	93.37	91.84	**1.61E-08**	**STAUROSPORINE**
**PRKX**	88.20	87.89	**8.22E-10**	**STAUROSPORINE**
**PYK2**	84.80	84.18	**7.88E-09**	**STAUROSPORINE**
**RAF1**	97.87	95.92	**7.73E-09**	**GW5074**
**RET**	96.61	95.52	**2.35E-09**	**STAUROSPORINE**
**RIPK2**	117.61	117.14	**8.69E-08**	**STAUROSPORINE**
**RIPK3**	105.29	103.43	**1.62E-06**	**GW5074**
**RIPK4**	70.34	69.52	**8.40E-07**	**STAUROSPORINE**
**RIPK5**	85.13	84.51	**4.06E-08**	**STAUROSPORINE**
**ROCK1**	92.34	91.79	**3.45E-10**	**STAUROSPORINE**
**ROCK2**	120.31	116.26	**4.78E-10**	**STAUROSPORINE**
**RON/MST1R**	109.25	108.33	**1.70E-07**	**STAUROSPORINE**
**ROS/ROS1**	99.82	98.20	**4.80E-10**	**STAUROSPORINE**
**RSK1**	95.71	93.23	**3.36E-10**	**STAUROSPORINE**
**RSK2**	101.56	101.15	**4.17E-10**	**STAUROSPORINE**
**RSK3**	106.50	104.36	**9.81E-11**	**STAUROSPORINE**
**RSK4**	99.31	98.96	**9.30E-11**	**STAUROSPORINE**
**SBK1**	99.68	98.75	**6.88E-08**	**STAUROSPORINE**
**SGK1**	91.57	91.31	**8.44E-09**	**STAUROSPORINE**
**SGK2**	93.15	92.43	**1.60E-08**	**STAUROSPORINE**

**SGK3/SGKL**	86.71	85.16	**6.93E-08**	**STAUROSPORINE**
**SIK1**	96.68	95.93	**1.95E-09**	**STAUROSPORINE**
**SIK2**	94.68	94.13	**1.05E-09**	**STAUROSPORINE**
**SIK3**	99.05	98.02	**1.22E-09**	**STAUROSPORINE**
**SLK/STK2**	104.82	104.72	**1.44E-08**	**STAUROSPORINE**
**SNARK/NUAK2**	96.77	95.16	**1.60E-09**	**STAUROSPORINE**
**SNRK**	106.97	104.90	**2.53E-08**	**STAUROSPORINE**
**SRMS**	103.81	102.72	**7.19E-06**	**STAUROSPORINE**
**SRPK1**	96.94	94.20	**3.49E-08**	**STAUROSPORINE**
**SRPK2**	105.57	101.81	**1.54E-07**	**STAUROSPORINE**
**SSTK/TSSK6**	110.95	110.94	**2.28E-07**	**STAUROSPORINE**
**STK16**	95.06	94.27	**2.35E-07**	**STAUROSPORINE**
**STK21/CIT**	80.57	76.31	**1.04E-07**	**STAUROSPORINE**
**STK22D/TSSK1**	103.64	103.46	**6.09E-11**	**STAUROSPORINE**
**STK25/YSK1**	96.45	89.21	**2.66E-09**	**STAUROSPORINE**
**STK32B/YANK2**	80.64	77.49	**2.04E-08**	**STAUROSPORINE**
**STK32C/YANK3**	90.62	88.14	**2.14E-07**	**STAUROSPORINE**
**STK33**	94.50	89.34	**1.66E-08**	**STAUROSPORINE**
**STK38/NDR1**	94.04	93.69	**6.92E-10**	**STAUROSPORINE**
**STK38L/NDR2**	97.02	96.17	**1.05E-09**	**STAUROSPORINE**
**STK39/STLK3**	93.60	88.95	**9.71E-09**	**STAUROSPORINE**
**SYK**	112.00	109.35	**6.71E-10**	**STAUROSPORINE**
**TAK1**	102.80	99.65	**9.00E-08**	**STAUROSPORINE**
**TAOK1**	78.70	76.28	**8.23E-10**	**STAUROSPORINE**
**TAOK2/TAO1**	65.36	62.95	**4.98E-09**	**STAUROSPORINE**
**TAOK3/JIK**	74.27	70.80	**6.11E-10**	**STAUROSPORINE**
**TBK1**	90.61	89.47	**3.60E-09**	**STAUROSPORINE**
**TEC**	114.91	107.05	**5.77E-08**	**STAUROSPORINE**
**TESK1**	107.93	107.90	**1.84E-07**	**STAUROSPORINE**
**TESK2**	95.91	93.89	**2.09E-05**	**STAUROSPORINE**
**TGFBR2**	115.48	111.45	**1.27E-07**	**LDN193189**
**TIE2/TEK**	95.20	94.89	**5.90E-08**	**STAUROSPORINE**

**TLK1**	112.20	108.87	**4.10E-08**	**STAUROSPORINE**
**TLK2**	94.27	93.05	**2.37E-09**	**STAUROSPORINE**
**TNIK**	90.34	88.69	**4.32E-10**	**STAUROSPORINE**
**TNK1**	114.99	114.65	**4.88E-09**	**STAUROSPORINE**
**TRKA**	90.82	88.70	**1.81E-09**	**STAUROSPORINE**
**TRKB**	102.70	97.43	**1.55E-10**	**STAUROSPORINE**
**TRKC**	107.51	103.04	**2.07E-10**	**STAUROSPORINE**
**TSSK2**	103.09	100.39	**5.89E-09**	**STAUROSPORINE**
**TSSK3/STK22C**	93.23	93.04	**5.15E-09**	**STAUROSPORINE**
**TTBK1**	96.47	92.57	**2.17E-05**	**SB202190**
**TTBK2**	98.81	95.13	**4.17E-06**	**SB202190**
**TXK**	108.58	105.04	**2.44E-08**	**STAUROSPORINE**
**TYK1/LTK**	97.83	96.95	**3.73E-08**	**STAUROSPORINE**
**TYK2**	98.01	97.52	**2.59E-10**	**STAUROSPORINE**
**TYRO3/SKY**	99.65	95.46	**3.11E-09**	**STAUROSPORINE**
**ULK1**	84.15	81.75	**1.09E-08**	**STAUROSPORINE**
**ULK2**	99.79	96.94	**1.89E-09**	**STAUROSPORINE**
**ULK3**	98.00	97.67	**3.46E-09**	**STAUROSPORINE**
**VRK1**	82.32	79.92	**9.63E-07**	**RO-31-8220**
**VRK2**	90.93	89.99	**1.07E-05**	**RO-31-8220**
**WEE1**	122.03	120.44	**6.43E-08**	**WEE-1 INHIBITOR**
**WNK1**	94.54	92.59	**2.19E-05**	**STAUROSPORINE**
**WNK2**	97.99	97.01	**5.23E-06**	**STAUROSPORINE**
**WNK3**	99.04	98.89	**2.30E-06**	**WEE-1 INHIBITOR**
**YES/YES1**	103.76	102.94	**2.64E-09**	**STAUROSPORINE**
**YSK4/MAP3K19**	97.31	96.19	**1.54E-08**	**STAUROSPORINE**
**ZAK/MLTK**	100.59	96.18	**9.91E-07**	**GW5074**
**ZAP70**	97.96	96.49	**1.80E-08**	**STAUROSPORINE**
**ZIPK/DAPK3**	106.62	106.08	**4.46E-09**	**STAUROSPORINE**
**AMPK (A1/B2/G2)**	103.29	98.8	**1.16E-10**	**STAUROSPORINE**
**AMPK (A1/B2/G3)**	110.89	109.96	**6.84E-11**	**STAUROSPORINE**
**AMPK (A2/B1/G2)**	93.32	92.34	**6.25E-11**	**STAUROSPORINE**

AMPK (A2/B1/G3)	95.97	94.2	8.88E-11	STAUROSPORINE
AMPK(A1/B1/G1)	105.06	103.11	3.39E-11	STAUROSPORINE
AMPK(A1/B1/G2)	98.92	98.01	4.47E-11	STAUROSPORINE
AMPK(A1/B1/G3)	103.55	94.84	6.31E-11	STAUROSPORINE
AMPK(A1/B2/G1)	113.68	113.01	3.06E-11	STAUROSPORINE
AMPK(A2/B1/G1)	108.89	107.51	6.04E-11	STAUROSPORINE
AMPK(A2/B2/G1)	104.71	103.92	6.93E-11	STAUROSPORINE
AMPK(A2/B2/G2)	99.16	96.79	7.06E-11	STAUROSPORINE
AMPK(A2/B2/G3)	96.32	94.69	1.76E-10	STAUROSPORINE
DNA-PK	120.24	115.14	1.96E-08	PI-103
EEF2K	105.61	102.7	9.83E-06	NH125
EIF2AK1	99.91	85.44	1.65E-08	GSK-2606414
EIF2AK2	101.31	100.95	5.20E-08	STAUROSPORINE
EIF2AK3	101.22	96.53	1.67E-09	GSK-2606414
EIF2AK4	97.69	97.32	3.54E-07	STAUROSPORINE
MTOR/FRAP1	114.62	106.11	7.95E-08	PI-103
PDK1/PDHK1	95.83	91.97	1.37E-05	GW5074
PDK2/PDHK2	105.2	103.26	5.75E-06	GW5074
PDK3/PDHK3	117.34	109.38	1.70E-06	GW5074
PDK4/PDHK4	115.94	109.5	1.13E-06	GW5074
TRPM7/CHAK1	98.34	94.35	1.15E-05	NH125
ABL1 (E255K)	98.87	98.38	7.42E-08	STAUROSPORINE
ABL1 (E255V)	89.48	89.20	2.38E-08	STAUROSPORINE
ABL1 (F317I)	103.45	100.87	1.81E-07	STAUROSPORINE
ABL1 (F317L)	102.87	101.24	1.75E-07	STAUROSPORINE
ABL1 (G250E)	107.49	103.39	1.43E-08	STAUROSPORINE
ABL1 (H396P)	102.05	100.63	3.80E-08	STAUROSPORINE
ABL1 (M351T)	102.32	98.62	5.06E-08	STAUROSPORINE
ABL1 (Q252H)	112.50	106.33	2.83E-08	STAUROSPORINE
ABL1 (T315I)	108.51	100.60	1.20E-08	STAUROSPORINE
ABL1 (V299L)	97.88	96.12	9.07E-08	STAUROSPORINE
ABL1 (Y253F)	87.94	84.12	4.30E-08	STAUROSPORINE

**ABL1 (Y253H)**	109.67	107.49	**2.39E-08**	**STAUROSPORINE**
**AKT1 (E17K)**	131.35	111.38	**2.51E-09**	**STAUROSPORINE**
**AKT2 (E17K)**	117.18	104.74	**2.14E-09**	**STAUROSPORINE**
**AKT3 (E17K)**	98.50	91.50	**4.83E-09**	**STAUROSPORINE**
**AKT3 (G171R)**	98.53	96.84	**5.23E-09**	**STAUROSPORINE**
**ALK (C1156Y)**	94.87	93.28	**8.61E-10**	**STAUROSPORINE**
**ALK (F1174L)**	99.52	98.23	**1.40E-09**	**STAUROSPORINE**
**ALK (F1174L)-EML4**	111.85	107.95	**2.91E-09**	**STAUROSPORINE**
**ALK (F1174L)-NPM1**	88.98	88.47	**2.86E-09**	**STAUROSPORINE**
**ALK (F1174S)**	94.52	91.20	**1.95E-09**	**STAUROSPORINE**
**ALK (G1202R)**	106.73	105.79	**6.15E-09**	**STAUROSPORINE**
**ALK (G1269A)**	114.13	108.95	**5.43E-10**	**STAUROSPORINE**
**ALK (G1269S)**	94.77	93.23	**2.52E-09**	**STAUROSPORINE**
**ALK (L1152R)**	97.16	91.03	**1.58E-09**	**STAUROSPORINE**
**ALK (L1196M)**	110.05	107.34	**1.32E-09**	**STAUROSPORINE**
**ALK (R1275Q)**	103.00	101.55	**4.98E-09**	**STAUROSPORINE**
**ALK (S1206R)**	100.11	100.01	**1.79E-09**	**STAUROSPORINE**
**ALK (T1151-L1152insT)**	117.18	111.26	**2.61E-09**	**STAUROSPORINE**
**ALK (T1151M)**	105.39	104.81	**9.07E-10**	**STAUROSPORINE**
**ALK-KIF5B (Kex24Aex20)**	109.08	105.09	**5.79E-09**	**STAUROSPORINE**
**ALK-KLC1 (Kex8Aex20)**	99.05	98.30	**4.74E-09**	**STAUROSPORINE**
**ALK-NPM1**	104.89	95.67	**3.45E-09**	**STAUROSPORINE**
**ALK-TFG**	101.77	100.93	**2.80E-09**	**STAUROSPORINE**
**ALK-TFG (Tex4Aex20)**	88.72	88.46	**1.86E-09**	**STAUROSPORINE**
**ALK-TPM1**	106.46	103.76	**9.46E-10**	**STAUROSPORINE**
**ALK-TPM3**	95.62	93.40	**2.30E-09**	**STAUROSPORINE**
**ALK2 (Q207D)**	89.06	86.37	**1.19E-08**	**LDN193189**
**ALK2 (R206H)**	100.17	98.34	**8.90E-09**	**LDN193189**
**AXL (R499C)**	95.92	93.83	**2.11E-09**	**STAUROSPORINE**
**BRAF (d485-489/P490Y)**	102.67	102.18	**8.40E-08**	**GW5074**
**BRAF (G464V)**	88.67	84.96	**1.19E-07**	**GW5074**
**BRAF (G469A)**	94.29	91.97	**1.44E-08**	**GW5074**

**BRAF (K601E)**	93.59	91.68	**5.24E-09**	**GW5074**
**BRAF (L597V)**	93.25	90.62	**6.60E-09**	**GW5074**
**BRAF (R506_K507insVLR)**	92.89	89.09	**2.33E-08**	**GW5074**
**BRAF (T599_V600insT)**	98.67	96.75	**9.63E-09**	**GW5074**
**BRAF (V599E)**	103.42	103.16	**4.41E-09**	**GW5074**
**BRAF (V600A)**	103.76	100.41	**7.62E-09**	**GW5074**
**BRAF (V600D)**	104.13	103.87	**6.27E-09**	**GW5074**
**BRAF (V600K)**	93.25	92.52	**7.71E-09**	**GW5074**
**BRAF-FAM131B (Fex2Bex9)**	92.82	92.55	**7.68E-09**	**GW5074**
**BRAF-KIAA1549 (Kex15Bex9)**	88.06	87.14	**4.36E-09**	**GW5074**
**BRAF-KIAA1549 (Kex16Bex9)**	101.14	95.89	**2.69E-08**	**GW5074**
**BRAF-SRGAP3 (Sex12Bex9)**	103.96	103.64	**7.80E-09**	**GW5074**
**BTK (C481S)**	109.52	109.04	**2.88E-08**	**STAUROSPORINE**
**BTK (E41K)**	90.52	90.29	**3.44E-08**	**STAUROSPORINE**
**BTK (P190K)**	94.52	94.47	**2.37E-08**	**STAUROSPORINE**
**c-Kit (A829P)**	96.57	94.29	**2.88E-10**	**STAUROSPORINE**
**c-Kit (d557-558)**	100.63	99.10	**1.74E-10**	**STAUROSPORINE**
**c-Kit (D816E)**	106.44	103.48	**9.21E-11**	**STAUROSPORINE**
**c-Kit (D816F)**	98.53	97.54	**4.25E-10**	**STAUROSPORINE**
**c-Kit (D816H)**	111.20	110.39	**3.62E-10**	**STAUROSPORINE**
**c-Kit (D816I)**	106.41	103.69	**2.22E-10**	**STAUROSPORINE**
**c-Kit (D816V)**	110.72	110.04	**9.63E-11**	**STAUROSPORINE**
**c-Kit (D816Y)**	95.20	92.76	**3.07E-10**	**STAUROSPORINE**
**c-Kit (D820E)**	102.80	101.03	**5.06E-10**	**STAUROSPORINE**
**c-Kit (D820Y)**	112.50	110.05	**6.60E-10**	**STAUROSPORINE**
**c-Kit (K642E)**	93.71	92.53	**1.28E-09**	**STAUROSPORINE**
**c-Kit (T670I)**	104.65	98.09	**3.13E-09**	**STAUROSPORINE**
**c-Kit (V559A)**	110.55	109.89	**3.88E-10**	**STAUROSPORINE**
**c-Kit (V559D)**	110.47	101.76	**3.37E-10**	**STAUROSPORINE**
**c-Kit (V559D/T670I)**	98.57	98.31	**1.05E-09**	**STAUROSPORINE**
**c-Kit (V559D/V654A)**	104.44	102.00	**3.07E-09**	**STAUROSPORINE**
**c-Kit (V560G)**	86.97	86.03	**1.19E-09**	**STAUROSPORINE**

**c-Kit (V560G/D816V)**	99.38	99.31	**1.50E-10**	**STAUROSPORINE**
**c-Kit (V560G/N822K)**	97.80	96.67	**3.51E-10**	**STAUROSPORINE**
**c-Kit (V654A)**	105.59	105.24	**4.03E-08**	**STAUROSPORINE**
**c-Kit (Y823D)**	101.08	99.99	**1.65E-09**	**STAUROSPORINE**
**c-MER (A708S)**	91.24	87.51	**1.04E-08**	**STAUROSPORINE**
**c-MET (D1228H)**	99.24	94.21	**1.73E-07**	**STAUROSPORINE**
**c-MET (D1228N)**	105.09	98.86	**1.37E-07**	**STAUROSPORINE**
**c-MET (F1200I)**	101.69	101.46	**2.39E-07**	**STAUROSPORINE**
**c-MET (K1244R)**	100.50	98.18	**1.01E-07**	**STAUROSPORINE**
**c-MET (M1250I)**	110.78	107.06	**4.02E-07**	**STAUROSPORINE**
**c-MET (M1250T)**	115.03	109.06	**1.68E-07**	**STAUROSPORINE**
**c-MET (P991S)**	105.03	98.01	**1.11E-07**	**STAUROSPORINE**
**c-MET (T1173I)**	105.73	104.02	**3.11E-07**	**STAUROSPORINE**
**c-MET (T992I)**	106.28	104.94	**1.90E-07**	**STAUROSPORINE**
**c-MET (V1092I)**	113.52	106.86	**4.18E-07**	**STAUROSPORINE**
**c-MET (Y1230A)**	90.71	88.59	**1.20E-07**	**STAUROSPORINE**
**c-MET (Y1230C)**	96.21	91.04	**1.02E-07**	**STAUROSPORINE**
**c-MET (Y1230D)**	92.04	91.23	**5.34E-08**	**STAUROSPORINE**
**c-MET (Y1230H)**	93.17	89.55	**2.75E-07**	**STAUROSPORINE**
**c-MET (Y1235D)**	65.82	64.15	**7.49E-08**	**STAUROSPORINE**
**c-MET-TFG (Tex5Mex15)**	99.78	97.80	**8.57E-08**	**STAUROSPORINE**
**c-Src (T341M)**	103.48	101.79	**1.99E-10**	**STAUROSPORINE**
**CHK2 (I157T)**	97.61	97.30	**6.71E-09**	**STAUROSPORINE**
**CK1epsilon (R178C)**	96.97	95.92	**2.09E-06**	**STAUROSPORINE**
**DDR2 (N456S)**	109.92	108.41	**7.74E-10**	**STAUROSPORINE**
**DDR2 (T654M)**	117.03	114.92	**1.37E-09**	**STAUROSPORINE**
**EGFR (A763_Y764insFHEA)**	100.13	99.86	**1.29E-07**	**STAUROSPORINE**
**EGFR (C775S/T790M/L858R)**	99.47	89.59	**2.93E-09**	**STAUROSPORINE**
**EGFR (C797A)**	102.23	101.79	**5.04E-08**	**STAUROSPORINE**
**EGFR (C797S)**	111.22	110.69	**1.85E-07**	**STAUROSPORINE**
**EGFR (C797S/L858R)**	97.76	97.33	**2.04E-08**	**STAUROSPORINE**
**EGFR (d746)**	102.60	102.15	**3.25E-08**	**STAUROSPORINE**

**EGFR (d746-750)**	87.69	86.98	**2.19E-08**	**STAUROSPORINE**
**EGFR (d746-750/C775S/T790M/L858R)**	101.68	98.09	**4.24E-09**	**STAUROSPORINE**
**EGFR (d746-750/C797A)**	100.45	99.77	**2.31E-08**	**STAUROSPORINE**
**EGFR (d746-750/C797S)**	90.77	90.07	**7.89E-09**	**STAUROSPORINE**
**EGFR (d746-750/T790M)**	95.17	94.86	**2.10E-09**	**STAUROSPORINE**
**EGFR (d746-750/T790M/C797S)**	97.21	96.87	**3.78E-10**	**STAUROSPORINE**
**EGFR (d747-749)**	100.93	100.52	**3.19E-08**	**STAUROSPORINE**
**EGFR (d747-749/A750P)**	97.52	97.37	**1.95E-08**	**STAUROSPORINE**
**EGFR (d747-752/P753S)**	91.74	91.39	**7.69E-08**	**STAUROSPORINE**
**EGFR (d752-759)**	100.02	98.10	**3.81E-07**	**STAUROSPORINE**
**EGFR (D761Y)**	100.15	99.49	**8.06E-08**	**STAUROSPORINE**
**EGFR (D770GY)**	95.25	94.64	**9.04E-08**	**STAUROSPORINE**
**EGFR (D770_N771insNPG)**	104.02	102.48	**1.29E-07**	**STAUROSPORINE**
**EGFR (G719C)**	116.51	114.64	**3.00E-07**	**STAUROSPORINE**
**EGFR (G719D)**	103.17	103.15	**2.71E-07**	**STAUROSPORINE**
**EGFR (G719S)**	89.83	89.13	**1.24E-06**	**STAUROSPORINE**
**EGFR (L747S)**	114.41	109.47	**1.21E-07**	**STAUROSPORINE**
**EGFR (L858R)**	104.01	103.22	**4.34E-08**	**STAUROSPORINE**
**EGFR (L858R, T790M)**	105.93	104.49	**3.03E-09**	**STAUROSPORINE**
**EGFR (L861Q)**	97.28	97.00	**2.06E-07**	**STAUROSPORINE**
**EGFR (T790M)**	105.04	104.35	**6.86E-09**	**STAUROSPORINE**
**EGFR (T790M/C797S)**	116.60	110.56	**1.30E-08**	**STAUROSPORINE**
**EGFR (T790M/C797S/L858R)**	95.88	95.80	**1.56E-09**	**STAUROSPORINE**
**ERBB2 (D769H)**	102.22	96.98	**8.85E-08**	**STAUROSPORINE**
**ERBB2 (D769Y)**	99.33	93.88	**2.06E-08**	**STAUROSPORINE**
**ERBB2 (P780_Y781insGSP)**	99.54	98.53	**1.08E-08**	**STAUROSPORINE**
**ERBB2 (R896C)**	87.73	86.48	**6.32E-08**	**STAUROSPORINE**
**ERBB2 (V777L)**	100.47	100.41	**7.71E-08**	**STAUROSPORINE**
**ERBB2 (V777_G778insCG)**	89.43	86.05	**1.23E-07**	**STAUROSPORINE**
**FGFR1 (V561M)**	101.92	95.88	**1.31E-09**	**STAUROSPORINE**

**FGFR1OP-FGFR1**	126.14	125.27	**6.73E-09**	**STAUROSPORINE**
**FGFR2 (E565G)**	103.32	99.28	**1.92E-09**	**STAUROSPORINE**
**FGFR2 (K526E)**	95.11	89.50	**3.66E-10**	**STAUROSPORINE**
**FGFR2 (K641R)**	101.74	101.04	**2.94E-09**	**STAUROSPORINE**
**FGFR2 (K659N)**	97.11	96.49	**4.94E-10**	**STAUROSPORINE**
**FGFR2 (N549H)**	104.63	102.28	**1.49E-09**	**STAUROSPORINE**
**FGFR2 (R612T)**	92.55	89.61	**5.52E-10**	**STAUROSPORINE**
**FGFR2 (V564F)**	105.72	104.84	**9.97E-11**	**STAUROSPORINE**
**FGFR3 (G697C)**	105.64	100.62	**9.82E-09**	**STAUROSPORINE**
**FGFR3 (K650E)**	126.21	122.20	**1.15E-08**	**STAUROSPORINE**
**FGFR3 (K650M)**	103.82	103.68	**1.64E-08**	**STAUROSPORINE**
**FGFR3 (K650Q)**	110.45	109.92	**1.25E-08**	**STAUROSPORINE**
**FGFR3 (V555M)**	86.41	85.76	**4.96E-10**	**STAUROSPORINE**
**FGFR4 (N535K)**	107.44	105.99	**4.29E-06**	**STAUROSPORINE**
**FGFR4 (V550E)**	108.17	106.13	**2.10E-06**	**STAUROSPORINE**
**FGFR4 (V550L)**	110.16	104.25	**4.70E-08**	**STAUROSPORINE**
**FGFR4 (V550M)**	114.51	111.50	**2.70E-08**	**STAUROSPORINE**
**FLT3 (D835Y)**	92.80	91.58	**1.72E-10**	**STAUROSPORINE**
**FLT3 (F594_R595insR)**	97.60	96.23	**1.81E-09**	**STAUROSPORINE**
**FLT3 (F594_R595insREY)**	91.61	91.08	**3.07E-09**	**STAUROSPORINE**
**FLT3 (ITD)**	81.41	81.39	**1.10E-09**	**STAUROSPORINE**
**FLT3 (ITD)-NPOS**	97.91	91.78	**3.19E-09**	**STAUROSPORINE**
**FLT3 (ITD)-W51**	104.28	103.12	**2.84E-09**	**STAUROSPORINE**
**FLT3 (R595_E596insEY**	85.99	84.70	**2.53E-09**	**STAUROSPORINE**
**FLT3 (Y591-V592insVDFREYEYD)**	106.00	104.21	**3.58E-09**	**STAUROSPORINE**
**FYN (Y531F)**	96.19	94.50	**3.24E-09**	**STAUROSPORINE**
**JAK2 (V617F)**	111.61	110.56	**1.71E-09**	**STAUROSPORINE**
**KSR1 (A635F)**	102.55	101.71	**9.04E-06**	**STAUROSPORINE**
**KSR1 (L639F)**	94.50	94.17	**9.20E-06**	**STAUROSPORINE**
**KSR2 (R676S)**	105.86	100.89	**8.54E-06**	**STAUROSPORINE**
**LRRK2 (G2019S)**	99.10	98.91	**3.53E-09**	**STAUROSPORINE**

**LRRK2 (I2020T)**	93.86	93.41	**7.42E-09**	**STAUROSPORINE**
**LRRK2 (R1441C)**	101.85	98.72	**6.18E-09**	**STAUROSPORINE**
**MEK1 (P124L)**	103.11	102.60	**1.75E-08**	**STAUROSPORINE**
**MELK (T460M)**	106.99	101.11	**1.63E-09**	**STAUROSPORINE**
**P38a (T106M)**	98.59	98.16	**4.10E-06**	**STAUROSPORINE**
**PDGFRa (D842V)**	103.31	100.58	**8.99E-10**	**STAUROSPORINE**
**PDGFRa (T674I)**	103.30	101.82	**7.08E-10**	**STAUROSPORINE**
**PDGFRa (V561D)**	98.45	96.41	**4.14E-09**	**STAUROSPORINE**
**PDGFRa-FIP1L1**	102.79	101.08	**1.65E-09**	**STAUROSPORINE**
**PDGFRb-TPM3**	89.12	88.38	**9.94E-10**	**STAUROSPORINE**
**PKD2 (G870E)**	91.70	90.56	**1.79E-09**	**STAUROSPORINE**
**PKMzeta**	103.08	103.06	**5.96E-08**	**STAUROSPORINE**
**PKN1-TECR (Tex1Pex10)**	103.13	100.76	**8.86E-10**	**STAUROSPORINE**
**RET (A883F)**	102.63	101.18	**1.08E-08**	**STAUROSPORINE**
**RET (E762Q)**	98.08	96.63	**3.97E-09**	**STAUROSPORINE**
**RET (G691S)**	98.96	97.49	**5.54E-09**	**STAUROSPORINE**
**RET (L790F)**	102.33	100.94	**4.13E-09**	**STAUROSPORINE**
**RET (M918T)**	97.70	96.48	**2.33E-09**	**STAUROSPORINE**
**RET (R749T)**	99.69	99.03	**5.32E-09**	**STAUROSPORINE**
**RET (R813Q)**	104.31	102.72	**6.71E-09**	**STAUROSPORINE**
**RET (R912P)**	108.02	104.27	**1.96E-09**	**STAUROSPORINE**
**RET (S891A)**	104.73	104.52	**8.58E-10**	**STAUROSPORINE**
**RET (S904A)**	98.71	98.34	**2.76E-09**	**STAUROSPORINE**
**RET (S904F)**	98.71	97.74	**2.76E-09**	**STAUROSPORINE**
**RET (V778I)**	99.57	97.50	**2.30E-09**	**STAUROSPORINE**
**RET (V804E)**	97.43	96.23	**9.80E-09**	**STAUROSPORINE**
**RET (V804L)**	100.07	99.11	**1.37E-09**	**STAUROSPORINE**
**RET (V804M)**	106.82	106.75	**7.18E-09**	**STAUROSPORINE**
**RET (Y791F)**	100.20	100.00	**1.54E-09**	**STAUROSPORINE**
**RET (Y806H)**	112.70	110.31	**6.92E-09**	**STAUROSPORINE**
**RET-BCR**	102.71	101.04	**2.35E-09**	**STAUROSPORINE**
**RET-CCDC6 (PTC1)**	97.69	95.44	**1.66E-09**	**STAUROSPORINE**

**RET-NCOA4 (PTC3)**	101.24	101.06	**2.62E-09**	**STAUROSPORINE**
**RET-PRKAR1A (PTC2)**	100.11	98.08	**2.80E-09**	**STAUROSPORINE**
**ROS1 (G2032R)**	98.76	96.97	**1.60E-08**	**STAUROSPORINE**
**ROS1-GOPC**	107.49	105.42	**1.34E-10**	**STAUROSPORINE**
**ROS1-TPM3**	104.70	104.12	**3.90E-10**	**STAUROSPORINE**
**RSK2 (I416V)**	102.87	102.27	**9.81E-11**	**STAUROSPORINE**
**RSK2 (L608F)**	101.32	97.99	**8.41E-11**	**STAUROSPORINE**
**TIE2 (A1124V)**	108.89	107.76	**2.85E-08**	**STAUROSPORINE**
**TIE2 (P883A)**	94.34	85.45	**2.01E-08**	**STAUROSPORINE**
**TIE2 (R849W)**	85.29	82.67	**6.58E-08**	**STAUROSPORINE**
**TIE2 (Y1108F)**	99.66	99.05	**5.34E-08**	**STAUROSPORINE**
**TIE2 (Y897C)**	97.84	96.71	**7.87E-08**	**STAUROSPORINE**
**TIE2 (Y897S)**	106.09	104.85	**1.85E-07**	**STAUROSPORINE**
**TRKA (A608D)**	102.15	101.85	**9.52E-10**	**STAUROSPORINE**
**TRKA (F589L)**	78.35	78.30	**2.05E-10**	**STAUROSPORINE**
**TRKA (G595R)**	103.39	102.16	**9.96E-09**	**STAUROSPORINE**
**TRKA (G595R/A608D)**	91.04	89.51	**2.06E-09**	**STAUROSPORINE**
**TRKA (G595R/G667A)**	103.75	100.84	**8.87E-10**	**STAUROSPORINE**
**TRKA (G595R/G667C)**	87.08	86.64	**1.41E-09**	**STAUROSPORINE**
**TRKA (G595R/G667S)**	96.01	95.32	**9.12E-10**	**STAUROSPORINE**
**TRKA (G595R/L657M)**	99.44	94.86	**8.35E-09**	**STAUROSPORINE**
**TRKA (G667C)**	102.76	98.48	**7.38E-10**	**STAUROSPORINE**
**TRKA (L657M)**	111.76	105.63	**8.06E-10**	**STAUROSPORINE**
**TRKA-TFG (TRK-T3)**	96.34	94.55	**5.37E-10**	**STAUROSPORINE**
**TRKA-TPM3**	115.18	111.95	**8.79E-10**	**STAUROSPORINE**
**TRKA-TPR**	128.19	120.23	**8.01E-10**	**STAUROSPORINE**
**TRKC (G623E)**	103.49	100.34	**2.85E-08**	**STAUROSPORINE**
**TRKC (G623R)**	102.62	99.81	**1.47E-08**	**STAUROSPORINE**
**TRKC (G623R/L686M)**	104.79	104.47	**1.15E-07**	**STAUROSPORINE**
**TRKC (L686M)**	115.44	107.87	**9.40E-09**	**STAUROSPORINE**
**YES1 (T348I)**	116.40	115.05	**3.15E-09**	**STAUROSPORINE**
**ZAP70 (Y319F)**	114.82	112.31	**7.95E-08**	**STAUROSPORINE**

Figure S2: Compound 28 Kinome Map

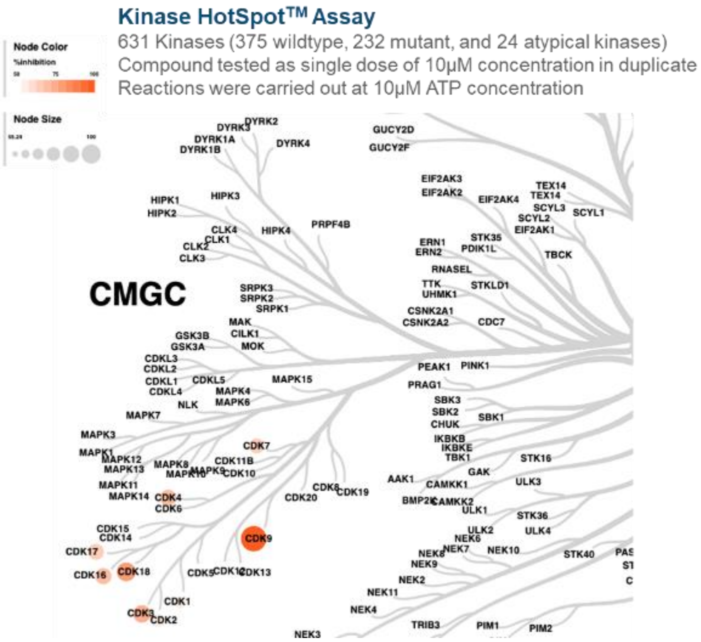

Compound **28** was tested against 631 kinases (375 wild type, 24 atypical and 232 mutant kinases). Compound was tested in single dose duplicate mode at a concentration of 10 µM. Control compound Staurosporine was tested in 10-dose IC50 mode with 3 or 4-fold serial dilution starting at 20 or 100 µM. Alternate control compounds were tested in 10-dose IC50 mode with 3 or 4-fold serial dilution starting at 10, 20, 50 or 100 µM. Reactions were carried out at 10 µM ATP. Data is represented as % Enzyme activity (relative to DMSO controls) and curve fits were performed where the control enzyme activities at the highest concentration of compounds were less than 65%.

Table S3. Stereochemical effect on CDK selectivity among analogues 28, 38, 39, and 40

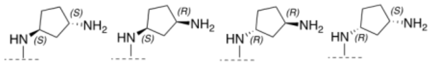

Comparison of relative IC_{50}

Compound	28	40	38	39
CDK9 IC_{50} (nM)	6	155	219	615
CDK1	703x	>65x	>46x	>16x
CDK2	1116x	>65x	>46x	>16x
CDK3	480x	>65x	>46x	>16x
CDK4	470x	>65x	>46x	>16x
CDK5	>1667x	>65x	>46x	>16x
CDK6	>1667x	>65x	>46x	>16x
CDK7	366x	>65x	>46x	>16x
CDK8	>1667x	>65x	>46x	>16x

CDK9/Cyclin T1, CDK1/Cyclin A, CDK2/Cyclin A, CDK3/Cyclin E, CDK4/CyclinD1, CDK5/P25, CDK6/Cyclin D1, CDK7/Cyclin H, CDK8/Cyclin C. [ATP] conducted at Km of CDK9 equal to 10μM. IC_{50} reported as geometric mean of duplicate runs. Blue = IC_{50} below top dose of 10μM; - = data not collected and/or assay not available.

Figure S3. Viability effects of 28 on TNBC cell lines

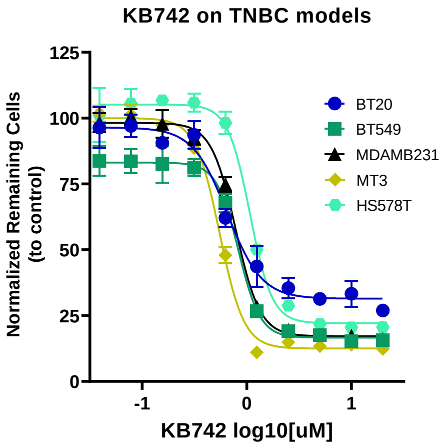

Table S4. OncoPanel[TM] Multiplexed Cytotoxicity Assay control compound in TNBC cell lines[a]

Cell line	Compound	Cell Count [b]EC_{50} (μM)	Cell Count [b]IC_{50} (μM)	Cell Count [b]GI_{50} (μM)	[b]Cell Count Activity Area	[c]Apoptosis 5X Fold Induction (μM)	[c]Apoptosis Emax	[d]G2/M cell cycle block (μM)	[d]G1/S cell cycle block (μM)
BT-20	Staurosporine	0.0316	0.0638	0.0236	2.58	N/A	4.6	N/A	0.00581
BT-549	Staurosporine	0.0102	0.0114	0.00556	4.39	0.115	6.9	N/A	0.00531
MDA-MB-231	Staurosporine	0.00118	0.00169	0.00124	5.26	0.00447	5.32	N/A	0.0122
MT-3	Staurosporine	0.0114	0.0135	0.00939	4.02	0.0607	10.32	N/A	0.0273
Hs 578T	Staurosporine	0.00164	0.00239	0.00145	4.99	0.00155	6.48	N/A	0.000753

[a]*BT-20, BT-549, MDA-MB-231, MT-3, and Hs 578T were treated with Staurosporine as an assay control in a serially dilution over 10 concentrations with a maximum of 0.1% DMSO over 72 hrs.* [b]*Cell proliferation was measured by the fluorescence intensity of an incorporated nuclear dye.* [c]*Apoptosis was measured by the fluorescence intensity of a fluorescently labeled antibody to activated caspase-3. The output is shown as a fold increase of apoptotic signal over vehicle background normalized to the relative cell count in each well. The concentration of test compound that caused a 5-fold induction in the caspase-3 signal is reported, indicating a significant apoptosis induction.* [d] *Cell cycle arrest was measured by labeling of mitotic cells with a fluorescently labeled antibody to phosphorylated histone H3.*

Figure S4. TNBC PDX models treated with 28

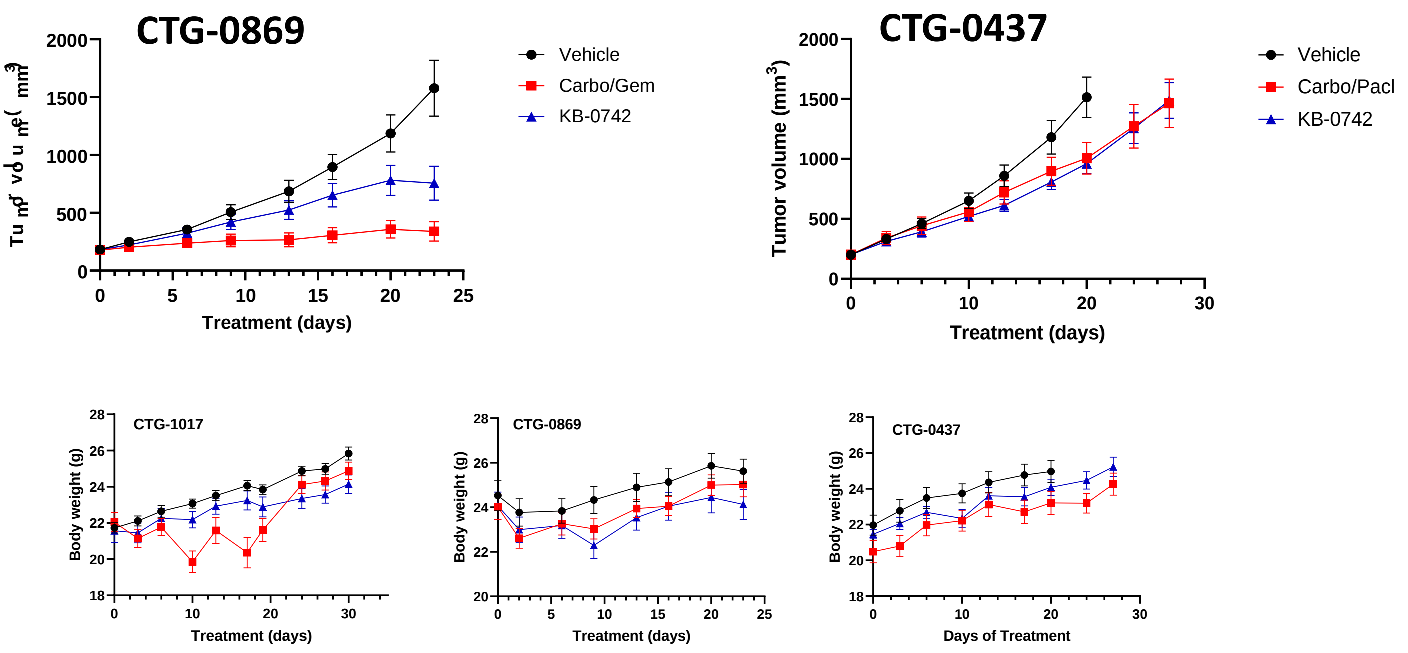

KB-0742 (28) inhibits tumor growth in 2 *MYC*-amplified PDX models of TNBC. Animals bearing established subcutaneous TNBC PDX models were treated with either vehicle (saline), KB-0742, or SOC chemotherapeutics. MYC CN and expression are indicated for each model. Vehicle and KB-0742 (CTG-0869 TGI = 59%, p = 0.0039; CTG-0437 TGI = 42%, p = 0.0172 versus vehicle) at 60 mg/kg were administered PO in all models using an intermittent dosing schedule of 3-days on, 4-days off for up to 4 weekly cycles. Models CTG-0869 received SOC carboplatin 40 mg/kg IP Q7D×3 + gemcitabine 100 mg/kg IP Q7D×3; and CTG-0437 received SOC carboplatin 50 mg/kg IP Q14D×2 + paclitaxel 10 mg/kg IV Q14D×2. Corresponding mean body weight over time graphs for each model shown.

Compound 28 NMR spectral characterization and HPLC purity

Figure S5. Variable temperature H[1] NMR 20 °C

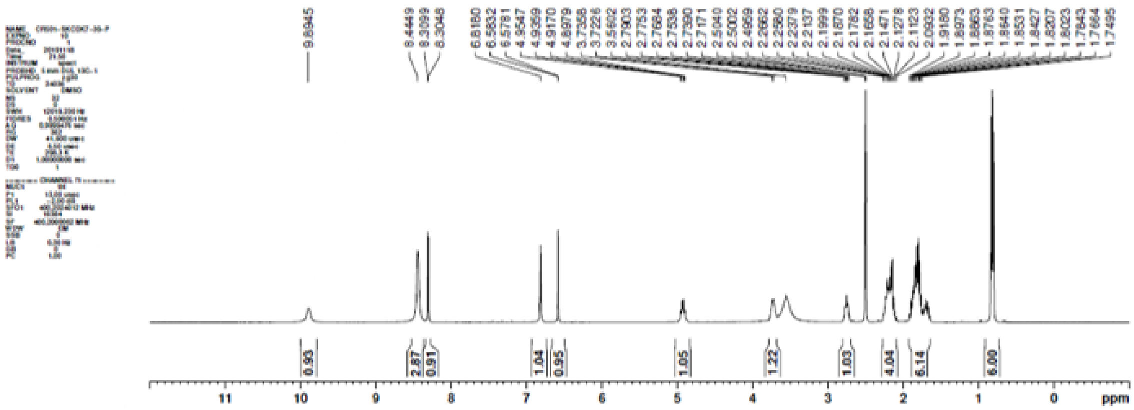

Figure S6. Variable temperature H[1] NMR 100 °C

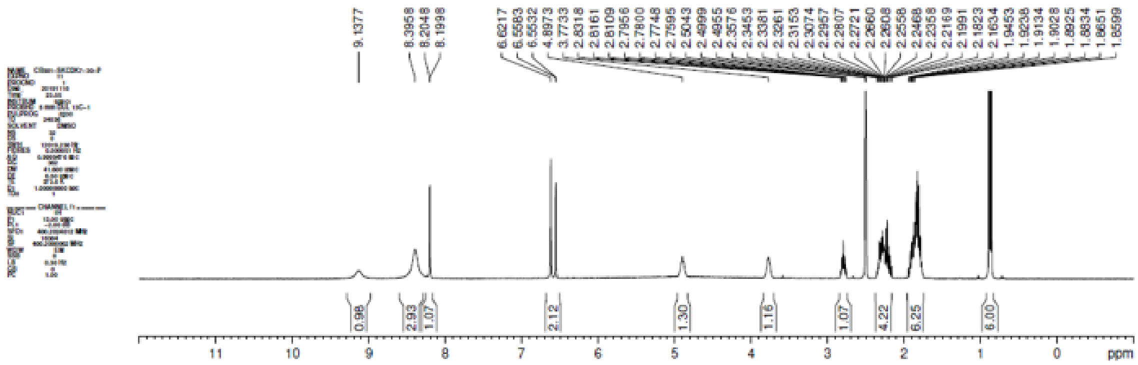

Figure S7. HPLC purity analysis

Sample ID: CR501-SKCDK7-30-P

Column: Gemini NX C-18 (100x 4.6 mm ; 5µl)

Diluent: DMSO

Mobile Phase: A: 10 mM NH4OAC in WATER, B: ACN

Inj. Vol.: 1 µl

Analysis Method : C:\CHEM32\1\METHODS\GEN_AB.M

Acq. Method : C:\Chem32\1\DATA\2019\NOV-19\18112019 2019-11-18 12-36-31\GEN_AB.M

Qualitative by HPLC (% by Area Normalization) at wavelength (λ_{210nm}): 99.32 %

Ref : / 18.11.19/1054

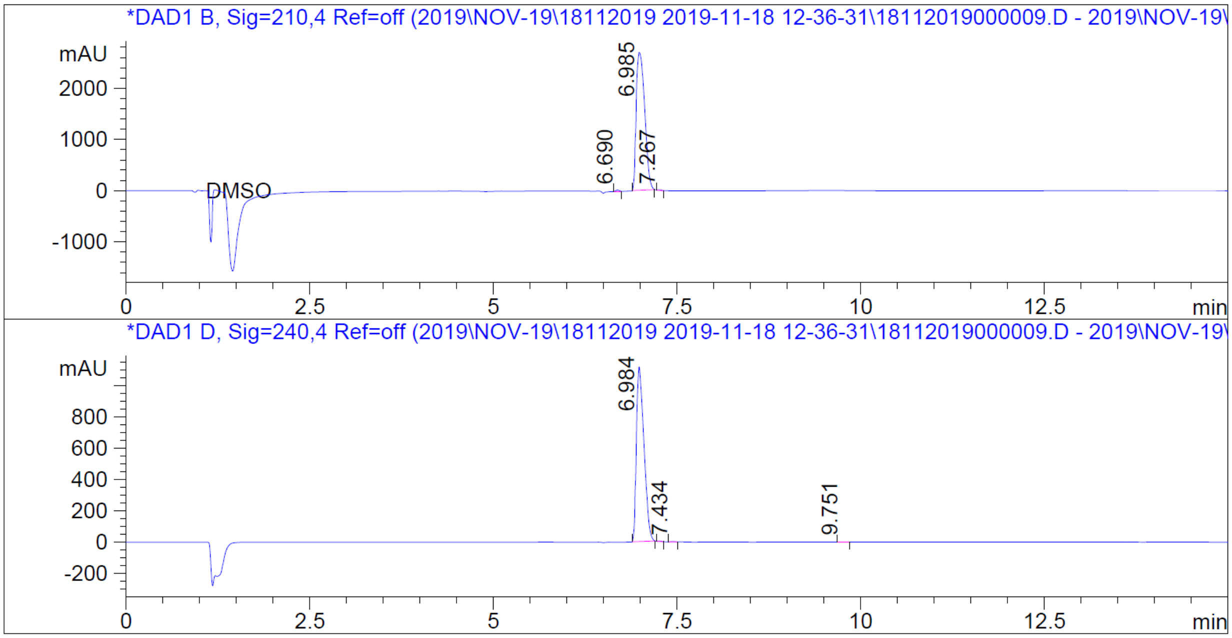

Signal 1 :DAD1 B, Sig=210,4 Ref=off

Peak #	RT [min]	Area	Area %
1	6.69	105.54	0.49
2	6.98	21560.90	99.32
3	7.27	42.92	0.20

Signal 2 :DAD1 D, Sig=240,4 Ref=off

Peak #	RT [min]	Area	Area %
1	6.98	8006.78	99.54
2	7.27	12.73	0.16
3	7.43	13.28	0.17
4	9.75	11.22	0.14

References

1. Barlaam, B.; Casella, R.; Cidado, J.; Cook. C. Discovery of AZD4573, a potent and selective inhibitor of CDK9 that enables short duration of target engagement for the treatment of hematological malignancies. **2020**. *J. Med. Chem.* 63: 15564-15590. doi: 10.1021/acs.jmedchem.0c01754.
2. Kabsch, W. XDS. *Acta. Crystallogr. D. Biol. Crystallogr.* **2010**. *66*(Pt 2): p. 125-32. doi: 10.1107/S0907444909047337.
3. Winn, M.D.; Ballard, C.C.; Cowtan, K.D.; Dodson, E.J.; Emsley, P.; Evans, P.R.; Keegan, R.M.; Krissinel, E.B.; Leslie, A.G.; McCoy, A.; McNicholas, S.J.; Murshudov, G.N.; Pannu, N.S.; Potterton, E.A.; Powell, H.R.; Read, R.J.; Vagin, A.; Wilson, K.S. Overview of the CCP4 suite and current developments. *Acta. Crystallogr. D. Biol. Crystallogr.* **2011**. *67*(Pt 4): p. 235-42. doi: 10.1107/S0907444910045749.
4. Emsley, P.; Lohkamp, B.; Scott, W.G.; Kowtan, K. Features and development of Coot. *Acta. Crystallogr. D. Biol. Crystallogr.* **2010**. *66*(Pt 4): p. 486-501. doi: 10.1107/S0907444910007493.
